# The experience of shared decision‐making for people with asthma: A systematic review and metasynthesis of qualitative studies

**DOI:** 10.1111/hex.14039

**Published:** 2024-04-13

**Authors:** Hui‐qi Kang, Yueming Pen, Yuanyuan He, Xiufen Yang, Jin Su, Qiaohong Yang, Weixiang Luo

**Affiliations:** ^1^ Jinan University, Tianhe District Guangzhou city Guangdong Province China; ^2^ Shenzhen People's Hospital (The First Affiliated Hospital, Southern University of Science and Technology), Luohu District Shenzhen city Guangdong China; ^3^ Department of Geriatric Shenzhen People's Hospital (The First Affiliated Hospital, Southern University of Science and Technology), Luohu District Shenzhen city Guangdong China

**Keywords:** adult, asthma, decision‐making, psycho‐oncology, qualitative research, shared

## Abstract

**Objectives:**

To identify, describe and synthesise the views and experiences of adults living with asthma regarding shared decision‐making (SDM) in the existing qualitative literature

**Methods:**

We conducted a comprehensive search of 10 databases (list databases) from inception until September 2023. Screening was performed according to inclusion criteria. Tools from the Joanna Briggs lnstitute were utilised for the purposes of data extraction and synthesis in this study. The data extraction process in this study employed the Capability, Opportunity and Motivation Model of Behaviour (COM‐B model) as a framework, and a pragmatic meta‐aggregative approach was employed to synthesise the collected results.

**Results:**

Nineteen studies were included in the metasynthesis. Three synthesised themes were identified: the capability of people living with asthma, the opportunities of people living with asthma in SDM, and the motivation of the people living with asthma in SDM.

**Conclusions:**

We have identified specific factors influencing people living with asthma engaging in SDM. The findings of this study can serve as a basis for the implementation of SDM in people living with asthma and provide insights for the development of their SDM training programs. The ConQual score for the synthesised findings was rated as low. To enhance confidence, future studies should address dependability and credibility factors.

**Practice Implications:**

This review contemplates the implementation of SDM from the perspective of people living with asthma, with the aim of providing patient‐centred services for them. The results of this review can benefit the implementation of SDM and facilitate information sharing. It offers guidance for SDM skills training among adults living with asthma, fosters a better doctor–patient relationship and facilitates consensus in treatment decisions, thereby enabling personalised and tailored medical care.

**Patient or Public Contribution:**

Three nursing graduate students participated in the data extraction and integration process, with two students having extensive clinical experience that provided valuable insights for the integration.

## 1 INTRODUCTION

Asthma is one of the most common and incurable chronic diseases, affecting approximately 241 million people worldwide.^1^ It is a respiratory inflammatory disease that is characterised by hyperresponsiveness of the respiratory tract^2^ and can cause dyspnoea and wheezing. Asthma morbidity and mortality rates have improved greatly over the past 15 years, however, undertreatment is still prevalent, and improving patients' and healthcare providers' understanding of when and how to adjust treatment is critical.^3^

Poor patient adherence is associated with outcomes such as reduced asthma control, intensified symptoms, increased healthcare expenditures and reduced quality of life for people living asthma.^4^ Shared decision‐making (SDM) refers to the involvement of both physicians and patients in the exchange of information, with clinicians providing evidence‐based information about treatment options and balanced information about the benefits, harms and uncertainties of each option, patients expressing treatment preferences and treatment plans being mutually agreed between the patient and the clinician.^5^ SDM has received increasing attention as patient self‐awareness has continued to increase together with their evidence‐based knowledge concerning diagnoses, complex treatment options, risk communication and value assessment increases.^6^ Studies have shown that SDM can improve treatment adherence, help asthma patients manage their condition and improve treatment outcomes.^7^ However, limited progress has been made in implementing SDM into clinical practice.^8^ To promote the development of SDM, Coulter et al.^9^ propose that patients need to be more strident in demanding a role in decision making.

Since SDM involves both patients and clinicians, and patient‐centred SDM should have an in‐depth understanding of patient preferences. Accordingly, it is crucial to determine the factors that influence decision‐making among people with asthma to provide targeted care guidance. Moreover, SDM that leads to the best clinical outcome must be consistent with a patient's own preferences and values. Upon review of the literature, it was found that the number of qualitative studies on the decision‐making experience of people living with asthma has increased, but original qualitative studies are not sufficient to provide a thorough understanding of patients' experience on SDM. Therefore, to synthesise qualitative studies on people living with asthma who have undergone SDM and explore the underlying reasons behind their high or low willingness to participate in SDM is important. The primary objective of this review was to assess the literature pertaining to SDM for people living with asthma as perceived by them using a metasynthesis methodology.

## 2 METHODS

### 2.1 Research design

The review protocol was registered with PROSPERO (CRD42023402509). The utilisation of this rigorous approach involved a comprehensive examination and interpretation of qualitative research findings pertaining to how asthma patients think about SDM. It is widely acknowledged that by employing this method, a novel and comprehensive interpretation of the collected data can be achieved, surpassing the mere summation of individual research studies, thereby yielding more profound and significant insights.^10^

### 2.2 Search strategy

We conducted a comprehensive search of 10 databases from February 2023 to August 2023. The search included 10 international databases: PubMed, Web of Science, MEDLINE, SSCI, Embase, ProQuest, CINAHL, Cochrane Library, Psychology and Behavioral Science Collection, APA PsycINFO. The following search terms were used to search by Boolean operators, adapted to syntax and subject headings of each database. And a set of keywords were identified and used to scope the literature. Finally, the references of the retrieved studies were manually examined to identify any supplementary studies that were not encompassed in the outcomes of the database search. Key initial terms encompassed: Asthma, Asthma*, Decision Making, Shared, Decision Making, Decision support techniques, Patient preference, Patient‐Centred Care, Patient Participation, Patient Education as Topic, Consumer Health Information and Pamphlets. Because some literature includes studies of both children and adults with asthma, and some mixed studies, such as randomised controlled trials, also contain qualitative studies, the search method is not limited by age and methodology. The full search strategy is detailed in Appendix S1.

### 2.3 Inclusion and exclusion criteria

#### 2.3.1 Inclusion criteria

(1) Study types: studies that used a qualitative methodology were included, and mixed‐methods studies were also included if their qualitative results were reported separately. (2) Participant types: We defined adults living with asthma as participants because we focused on the literature on adult experiences since children's experiences of asthma are reported primarily through their parents, and the experiences of adolescents are markedly different from those of adults or children. (3) Outcome type: we focused on asthma SDM. (4) Language type: the search was conducted in the English language only due to a lack of resources for translation. This review considered studies that focused on qualitative data or included a qualitative aspect, including but not limited to designs such as phenomenology, grounded theory, ethnography, illness narrative and action research.

#### 2.3.2 Exclusion criteria

(1) Study types: studies were excluded if they involved quantitative methods or literature or systematic reviews lacking any original research findings. (2) Participant types: studies of asthma SDM specifically among people with cognitive impairment were excluded, as such interventions may have a different focus.

### 2.4 Selection of studies

**Figure 1:**
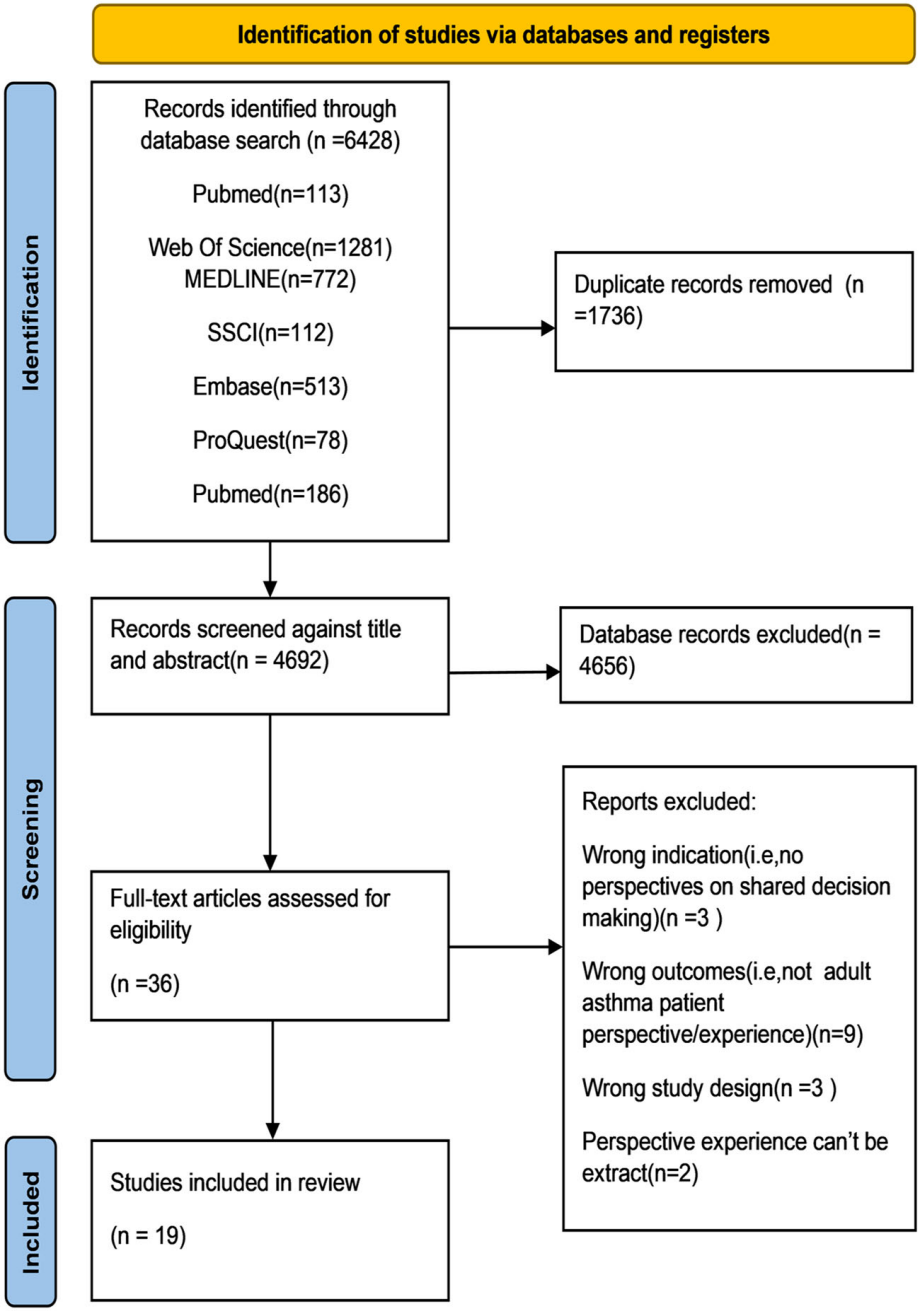
Flowchart of the search process (PRISMA).

A total of 6428 publications were identified through the search and imported into Endnote X20 software. After removing duplicates using Endnote X20 and manual searching, 1736 duplicates were eliminated, resulting in 4692 studies assessed for relevance based on title and abstract. Following screening, 36 studies met the selection criteria, and their full texts were retrieved for further evaluation. After carefully reviewing the full text, 19 studies were deemed eligible for quality appraisal. The PRISMA diagram presents the outcomes of the search (Figure 1). Two reviewers (K. H. Q. and H. Y. Y.) independently screened the literature with the assistance of a third party in case of disagreement. The data extraction form was cross‐checked for accuracy.

### 2.5 Appraisal of methodological quality

The research methodology rigour of each publication was critically appraised using the Joanna Briggs Institute Qualitative Assessment and Review Instrument.^11^ Questions that answered ‘yes’ were scored 1 point, and studies with an overall score of 5 or less were considered low‐quality and excluded from the synthesis of previous similar studies.^11^, ^12^ Due to insufficient information available for assessing eligibility criteria, 17 were excluded from the analysis as a result of the quality appraisal process (Appendix S2). A total of 19 studies were selected for inclusion in the systematic review. Two reviewers (K. H. Q. and H. Y. Y.) independently conducted critical appraisals of each selected study. Any disagreements between the reviewers were resolved through discussion or with the assistance of a third reviewer within the team to achieve consensus. Table 1 provides a quality assessment of the studies included in this review.

**Table 1:** Quality assessment of the included studies.

Author (year)	Q1	Q2	Q3	Q4	Q5	Q6	Q7	Q8	Q9	Q10	Total score
Alzayer (2002)	Y	Y	Y	Y	Y	Y	Y	N	Y	Y	9
Canny (2023)	Y	Y	Y	Y	Y	Y	Y	N	Y	Y	9
Caress (2002)	Y	Y	Y	Y	Y	Y	Y	Y	Y	Y	10
Eassey (2019)	Y	Y	Y	Y	Y	N	Y	Y	Y	Y	9
Gagné (2022)	Y	Y	Y	Y	Y	N	N	Y	Y	Y	8
George (2015)	Y	Y	Y	Y	Y	Y	U	Y	U	Y	8
George (2019)	Y	Y	Y	Y	Y	U	N	Y	Y	Y	8
George (2020)	Y	Y	Y	Y	Y	Y	Y	Y	Y	Y	10
Hannane (2019)	Y	Y	Y	Y	Y	N	N	N	Y	Y	7
Hoskins (2016)	Y	Y	Y	Y	Y	N	Y	Y	Y	Y	9
Kopnina (2010)	Y	Y	Y	Y	Y	N	N	Y	Y	Y	8
Lee (2023)	Y	Y	Y	Y	U	Y	Y	U	Y	Y	8
Melton (2014)	N	Y	Y	Y	Y	N	Y	Y	Y	Y	8
Mowrer (2015)	Y	Y	Y	Y	Y	N	Y	Y	Y	Y	9
Newcomb (2010)	Y	Y	Y	Y	Y	N	Y	Y	N	Y	9
Norful (2020)	Y	Y	Y	Y	Y	Y	N	Y	Y	Y	9
Tapp (2014)	Y	Y	Y	Y	Y	U	Y	Y	Y	Y	9
Tapp (2017)	Y	Y	Y	Y	Y	N	U	Y	Y	Y	8
Young (2011)	Y	Y	Y	Y	Y	U	Y	Y	Y	Y	9

*Note*: Q1: Is there congruity between the stated philosophical perspective and the research methodology?

Q2: Is there congruity between the research methodology and the research question or objectives?

Q3: Is there congruity between the research methodology and the methods used to collect data?

Q4: Is there congruity between the research methodology and the representation and analysis of data?

Q5: Is there congruity between the research methodology and the interpretation of results?

Q6: Is there a statement locating the researcher culturally or theoretically?

Q7: Is the influence of the researcher on the research, and vice versa, addressed?

Q8: Are participants, and their voices, adequately represented?

Q9: Is the research ethical according to current criteria or, for recent studies, is there evidence of ethical approval by an appropriate body?

Q10: Do the conclusions drawn in the research report flow from the analysis, or interpretation, of the data?

Abbreviations: N, no; U, unclear; Y, yes.

### 2.6 Data extraction and synthesis

Meta‐aggregation, a prominent method of metasynthesis, was employed in this systematic review to amalgamate the findings derived from the included studies.^13^ Specifically, the first author extracted the following information: author and year, methodology, methods, platform and length of interview, Country, population, patient gender, aim of the selected studies, place of data collection and result theme (Table 2).

**Table 2:** Consort diagram of the identification of studies.

Author (first)	Methodology	Methods	Platform/length of interview	Country	Population	Gender of patients (number of male/female)	Aim	Place of data collection	Result theme (circle represent hemes and bracket represent subthemes)
Alzayer et al. (2002)^14^	Q	Qualitative approach and a semi‐structured interviews.	Face‐to‐face interviews/25 min	Saudi Arabia	Asthma	*N* = 23 (4/19)	The aim of this study was to explore the experience of Saudi participants in managing their asthma and their perspectives about using future pharmacy‐based services for asthma management.	At clinic or community pharmacy or a café in the selected shopping malls	① **Participants experience of asthma**. ②**Participants' beliefs and perceptions about health and medicines**: (1) Asthma literacy and information needs. (2) Beliefs in alternative medicine systems.
Canny et al. (2023)^14^	Q	Qualitative approach and a semi‐structured interviews.	One‐on‐one telephone interviews/30–45 min	England	Asthma	*N* = 17 (5/12)	To explore patient experiences relating to their asthma diagnosis and to understand how a clinical decision support system (CDSS) could be used to improve the diagnostic process for patients.	Telephone	**①Diagnosis: The patient experience**. (1) Knowledge and understanding of asthma (2) Communication (3) Receiving and retaining information (4) Self‐management **② CDSS: Patient experience and views** (1) Patient experiences of screen sharing (2) Online health information use (3) Patient views on an asthma CDSS (4) Barriers and facilitators to a CDSS being used
Caress et al. (2002)^15^	Q	Tape‐recorded focused‐conversation style interviews. Interview topic guide derived from the literature. Sort cards employed to provide the focus for the exploration of role preferences.	Face‐to‐face interviews/NA	England	Asthma	*N* = 32 (17/15)	To explore preferred treatment decision‐making roles, and rationales for role preference, and to identify perceived facilitators to and barriers from attaining preferred role.	At home	**① Rationales for role preference**: (1) Patient's level of knowledge (2) Trust in health professionals and in efficacy of treatment (3) Length of time with condition (4) Severity of condition at decisional juncture (5) Lifelong nature of asthma (6) Perception that ‘It is my body’ (7) Characteristics of the individual (8) Patient's response to health professionals **② Other considerations**: (9) Specialism versus generalism (10) The role of health professionals other than clinicians
Eassey et al. (2019)^16^	Q	In‐depth semi‐structured interviews that were video‐ and/or audio‐recorded. and transcribed. Qualitative interview approach.	Face‐to‐face interviews/1.5–4 h	Australia	Severe Asthma	*N* = 29 (14/15)	To explore the role of autonomy in patients' narratives about their experiences of living with and managing severe asthma.	In the respondents' homes, or elsewhere	**① The desire to live an ‘unconstrained’ life** (1) Health‐care interactions (2) Employment **② Preservation of self‐identity** (1) Maintaining valued roles (2) Searching for normality
Gagné et al. (2022)^17^	M	A list of 15 critical issues were identified in focus groups and interviews. Verbatim transcripts were imported into MAXQDA 2020. MG read and coded transcripts line by line. Qualitative content analysis.	Virtual interviews/NA	Canada	Mild asthma	*N* = 21 (9/12)	To develop an electronic decision aid to guide discussions about the pros and cons of treatment, and to identify and integrate user preferences.	Online	① Content preferences ② Format preferences ③ Preferences for process
George et al. (2015)^18^	Q	Qualitative approach and semi‐structured open‐ended interviews.	Face‐to‐face interviews/20–40 min	America	Asthma	*N* = 35 (10/15)	To identify urban adults' perceptions of facilitators and barriers to asthma control, including the role of self‐care, medications, environmental trigger remediation and primary care.	At home	① Monitoring and responding to deteriorating control ② Beliefs about inhaled corticosteroid (ICS) and short‐acting beta‐agonist ③ Triggers avoidance/remediation ④ Role of primary care
George et al. (2016)^19^	Q	A qualitative analysis of transcripts from 33 audio‐recorded primary care visits using conventional content analysis techniques.	Face‐to‐face interviews/9–50 min	America	Asthma	*N* = 33 (1/32)	To explore whether patients' personal beliefs about ICS and integrative medicine (IM) are discussed at routine primary care visits for asthma.	At clinic	① Negative ICS beliefs ② IM use for asthma ③ Decision‐making ④ Healthy lifestyles
George et al. (2020)^20^	Q	Qualitative descriptive methodology that guided the design and the conduct of focus groups.	Focus group interviews/2.5–3 h	America	Asthma	*N* = 32 (NA)	To understand how ICS nonadherence could be addressed from the perspective of African American(AA) adults with asthma, their family and friends.	Two urban federally qualified health centres	① To be heard and respected ② Wish to receive patient‑centred care ③ Underscore the risk of ICS ④ Nonadherence
Hannane et al. (2019)^21^	Q	Unstructured interviews. Grounded‐theory approach. Qualitative content analysis.	Face‐to‐face interviews/12–60 min	France	Asthma	*N* = 30 (12/18)	To explore the perceptions of French adult asthma patients regarding their care pathway	In GP clinic	① The stakeholders of patients ② Patient relationships with healthcare professionals ③ Interprofessional collaboration
Hoskins et al. (2016)^22^	M	Individual semistructured interviews, analysed following the guidelines for thematic framework analysis.	Face‐to‐face interviews (telephone interviews were offered as an option)/30–40 min	England	Active asthma	*N* = 14 (7/7)	To explore (1) the experience, acceptability and perceived usefulness of the GOAL tool and goal‐setting process; (2) the perceived impact of the intervention on self‐management, quality of life and clinical practice; (3) the perceived change in professional‐ patient communication; and (4) experiences and accept‐ability of all elements of the trial.	NA	① Coherence: Meaning and sense making by participants ② Cognitive participation: Commitment and engagement by participants ③ Collective action: The work participants do to make the intervention function ④ Reflexive monitoring: Participants reflect on or appraise the intervention ④ Implementation
Kopnina (2010)^23^	Q	Semi‐structured interviews and focus groups conducted.	Focus group interviews/NA	New Zealand	Asthma	*N* = 19 (7/12)	The study examined the causes of patient noncompliance with the prescribed medical regime.	NA	① Perception of illness and own identity ② Encounters with medical practitioners ③ Encounter with print and online information on asthma and medical treatments ④ Encounter with patients' social groups (patient organisations, family, peers, etc.)
Lee (2023)^24^	M	A randomised controlled t rial (RCT)	Virtual focus groups/	America	Asthma	*N* = 9 (NA)	To assess the usability, acceptability and preliminary effectiveness of an electronic SDM application, the ACTION (Active Conversation in asthma Treatment shared decision‐making) app, that addressed medication, nonmedication and COVID‐19 concerns for asthma.	Online	① The ACTION app is an insightful communication tool about asthma ② The ACTION app and efficiency in the office ③ The logistics of the ACTION app in asthma clinic visits
Melton et al. (2014)^25^	M	Semi‐structured interviews. Interview data were analysed using interpretative phenomenological analysis.	Face‐to‐face interviews/45–60 min	America	Asthma	*N* = 4 (0/4)	To use patients' experiences of managing asthma to better understand the relationship between health literacy and health outcomes.	NA	① Information desired versus information received ② Trial and error ③ Expectations of the patient–provider relationship
Mowrer et al. (2015)^26^	Q	Focus groups held every 6 months for 3 years. Qualitative content analysis.	Face‐to face interviews/15–60 min	America	Asthma	*N* = 200 (NA)	To further explore patient and provider perceptions of asthma and asthma care as part of a larger Asthma Comparative Effectiveness Study.	At the clinic	① Impact on the care home staff and concerns for the care sector
Newcomb et al. (2010)^27^	Q	Semistructured interviews. One conversation unit was randomly selected from each subject and coded by two investigators separately.	Face‐to‐face interviews/20–45 min	America	Asthma	*N* = 104 (9/95)	To describe what adult patients with asthma report about their experiences with their own self‐management behaviour and working with their clinicians to control asthma.	In private examination room	① Personal constraints ② Communication failures ③ Social constraints ⑤ Medication Issues
Norful (2020)^28^	Q	Clinical visits for uncontrolled asthma were audio recorded and inductively analysed using methods adapted from grounded theory methodology.	Face to face interview/8–28 min	America	Asthma	*N* = 83 (17/66)	To explore how Black adults with uncontrolled asthma and their primary care providers communicated about ICS nonadherence and used shared decision‐making to identify strategies to increase ICS use.	At clinic	① ICS misuse and lack of knowledge ② External influences yielding personal misconceptions ③ Patient–provider communication to individualise plan of care
Tapp et al. (2014)^29^	M	The RE‐AIM framework and qualitative analysis. A facilitator from outside the group led a discussion using questions from a previously developed participatory evaluative focus group guide.	Focus group interviews/20–30 min	America	Asthma	*N* = 125 (NA)	This study describes the participatory approach used to adapt and implement an evidence‐based asthma SDM intervention into primary care practices.	NA	**① Intervention implementation**: (1) Intervention sustainability (2) Productivity (3) Tailoring (4) Stakeholder identification (5) Intervention training **② Participatory process**: (1) Inclusion (2) Knowledge exchange (3) Open communication (4) Investment (5) Productivity
Tapp et al. (2017)^30^	Q	Description of a case study of patient engagement in outcomes research and examination of the variety of roles patients are engaged in and the associated impact on the study.	Telephone conference/NA	America	Asthma	*N* = 16 (NA)	To describe various patient roles and impact within these large outcomes research study.	NA	① Lived experience patients ② Caregiver advocates ③ Research participants ④ Patient advisory board
Young et al. (2011)^31^	M	RCT, interviews were conducted with a randomly selected sample of 15 intervention group participants after all 3‐month postintervention follow‐up surveys were completed. An interviewer used a standardised guide to conduct confidential, one‐on‐one telephone interviews.	One‐on‐one telephone interviews/NA	America	Asthma	*N* = 15 (NA)	To assess the feasibility, acceptability and preliminary impact of a telepharmacy intervention in an underserved, rural asthma patient population.	Telephone	① Positive and very helpful ② Improve self‐management ③ Questions and immediate feedback ④ Time

Abbreviations: COVID‐19, coronavirus disease 2019; GP, general practitioner; M, mixed study; PPE, personal personal equipment; Q, mualitative study; SDM, shared decision‐making.

Next, the reviewers conducted a meticulous examination of the included studies, identified and extracted pertinent findings from the studies, which encompassed direct quotations, observations, or statements that effectively illustrate or substantiate the outcomes derived from the primary investigations. Subsequently, all extracted findings and illustrative data were evaluated independently by two reviewers (K. H. Q. and Y. X. F.) who individually assigned a level of credibility to each one. Regarding qualitative evidence, three levels of credibility were considered: ‘Unequivocal (U)’ denoted evidence beyond a reasonable doubt, typically factual, directly reported or observed findings not open to challenge; ‘Credible (C)’ pertained to plausible findings based on interpretations in light of the available data and via the theoretical framework; ‘Unsupported (Un)’ indicated findings lacking support by the data.^32^ No discrepancies arose regarding these levels of credibility.

**Figure 2:**
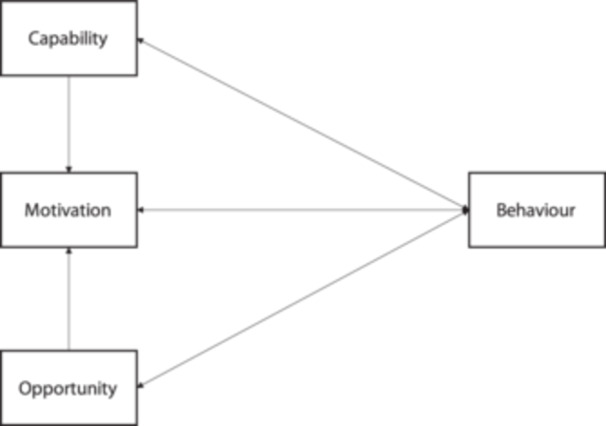
The Capability, Opportunity and Motivation Model of Behaviour model—a framework for understanding behaviour.

All data were extracted by employing the Capability, Opportunity and Motivation Model of Behaviour (COM‐B model) as a guiding framework. The engagement of people living with asthma in SDM is basically considered a behaviour, the COM‐B model emphasises that behaviour is influenced by the interaction of an individual's capability, opportunity and motivation. Only when these three elements are present can a particular behaviour be facilitated.^33^ It therefore offers valuable insights into the behaviour of people living with asthma when engaging in SDM. Capability refers to the skills, knowledge and abilities required for individuals to engage in a particular behaviour. Opportunity encompasses the various factors within the environment (such as social, cultural and institutional) that either facilitate or hinder individual behaviour. Motivation concerns the internal psychological factors that drive individuals to choose specific behaviours based on their motivations and goals.^34^ To effectively employ and extend the COM‐B model, as illustrated in Figure 2, we explored how capability, opportunity and motivation interact to determine the behaviour of people living with asthma in SDM. The unidirectional and bidirectional arrows in Figure 2 represent the potential influences between components within the system. For instance, opportunities can affect motivations, as can abilities; formulation of behaviour can alter abilities, motivations and opportunities.

We categorised the findings according to semantic similarities and subsequently subjecting these categories to a metasynthesis that yielded synthesised outcomes through meta‐aggregation.^35^ Meta‐aggregation involves a three‐step thematic analysis. In the first step, the first author performed meticulous line‐by‐line coding of patients' quotations to identify all important and relevant themes and concepts contained therein (Appendix S3). In the second step, two authors (K. H. Q. and H. Y. Y.) organised these codes into descriptive themes, aiming to group to best represent their underlying similarities and shared characteristics (Appendix S4). Finally, in the third step, two authors (K. H. Q. and H. Y. Y.) developed analytical themes that better illuminate the underlying patterns and trends across the focal texts (Appendix S5). The entire process involved the systematic engagement of two authors (K. H. Q. and H. Y. Y.), both individually and collaboratively, to provide a novel interpretation transcending the selected studies. This analysis also entailed ongoing dialogue and introspection between these two authors. Finally, we used the ConQual tool to assess the level of confidence regarding to the synthesised findings.^36^

## 3 RESULTS

### 3.1 Characteristic of the studies

Below, the PRISMA diagram is presented in Figure 1. In addition, Table 2 describes the characteristics of the selected studies. Most of the studies were conducted in America (*n* = 11),^18^, ^24^, ^25^, ^26^, ^27^, ^28^, ^29^, ^30^, ^31^, ^37^, ^38^ England (*n* = 3),^15^, ^22^, ^39^ Australia (*n* = 1),^16^ France (*n* = 1),^21^ New Zealand (*n* = 1),^23^ Canada (*n* = 1),^17^ and Saudi Arabia(*n* = 1).^14^ Quality assessment revealed that most of these studies met most Joanna Briggs Institute criteria (Table 1).

### 3.2 Metasynthesis of qualitative data

Data extraction yielded 250 quotes from the 19 selected studies (Appendix S4), then 250 quotes were organised into descriptive themes (Appendix S5). According to metasynthesis, three themes were identified: (1) The capability of people living with asthma, (2) The opportunities of people living with asthma in SDM and (3) The motivation of the people living with asthma in SDM (Appendix S5).

### 3.3 Synthesised finding 1: The capability of people living with asthma in SDM

As we know, the concept of capability pertains to an individual's cognitive and physical prowess to partake in various activities, enshrining the essential knowledge and competencies required for such engagement. It is important to recognise that the ability of adults with asthma to join SDM has a profound impact on the participant experience.

#### 3.3.1 Attitude towards asthma

First, people living asthma have diverse views regarding participation in SDM, which are classified as being positive, neutral, or negative.^15^, ^16^, ^18^, ^21^, ^22^, ^24^, ^26^, ^29^, ^30^, ^37^, ^39^, ^40^ The majority of them, who have a positive attitude, respect asthma care and actively seek professional advice (most of whom are women); as a result, they are eager for medical institutions to impart expert information about asthma.

Never give up … If you think there's something wrong, that someone's not saying, speak up. You've seen something [health‐related issue that her healthcare providers didn't pick up] obviously but they didn't take the time to see … speak up. (Jane, 51‐year‐old woman)^16^

Second, some people living with asthma were neutral about participation in decision‐making, stating that they could participate in decision‐making communication, but once faced with a critical moment in decision‐making, they tended to shift the responsibility for the decision to the physician because they felt that the decision was beyond their scope and therefore avoided participation. This preference also reflects their recognition of physicians' expertise.

But I think when it comes to that kind of decision, it's got to come from a qualified (person). (PC07)^15^

Furthermore, negative attitudes and behaviours are displayed when the people living with asthma lacks decision‐making capability. They didn't want to take part in SDM.

I've got the disease or ailment, whatever you call it—I'm not the man with the knowledge—the doctor's the man with the medical knowledge, not me. (PC02)^15^

#### 3.3.2 Asthma knowledge acquisition, understanding and processing

The lack of knowledge acquisition, understanding and processing capability can affect the experience and motivation of people living with asthma to participate in SDM to some extent.^17^, ^19^, ^21^, ^23^, ^25^, ^26^, ^30^ Health care institutions, the Internet and patients' social networks are the three primary sources of information transfer, which is a crucial step in the decision‐making process. For people living with asthma, having access to specialised information on their condition is a crucial tool for decision‐making, and information of varying quality can have an impact. They are often informed about this intricate process in accordance with their level of receptivity; yet, they frequently have trouble understanding medical language that is highly technical.

Some of the wording might be very hard to comprehend … So maybe simpler words, for people who are not that bright so to speak. (Patient 4, FG1)^40^

As information transmitters, healthcare providers have limits when it comes to educating patients about health‐related topics such as inhaler usage education, the use of decision aids and the way to get and recognise right information, according to qualitative research. Additionally, when the people living with asthma who are the recipients of information do not know how to use their inhalers, this can result in the ineffective daily management of asthma as well as incorrect asthma decision‐making behaviours. For instance, some of them do not know the difference between asthma‐control inhalers and rescue inhalers, use the two interchangeably, or even use only the inhaler that works for them.^26^ As a result, information transmitters (healthcare professionals) and information receivers (people living with asthma) play crucial roles in the pathway for the dissemination of asthma information, and the absence of a path for information to be disseminated and received by either party can have a negative impact on decision‐making in the context of asthma.

I think your message gets a little bit lost in all the other information that's given like it's maybe too detailed […]. (Patient 1, FG1)^40^

[The decision aid] is a very text‐based. […] I'm not positive on that. (Patient 15, FG4)^40^

People living with asthma can get information from sources other than their healthcare professionals through the Internet and their own social networks, giving them more information to help them make decisions about their asthma. It is more difficult to find information from the Internet that is helpful for making decisions about asthma because it is very large and complex, the quality of this information varies, and people living with asthma are typically confused by it. As a result, it is important for people living with asthma to decide whether asthma information on the Internet can be used. Information on asthma that is collected through social networks is frequently based on family, friends and patient experiences. Usually this kind of asthma information is empirical and individualised. People living with asthma frequently test this empirical knowledge obtained from social networks on themselves to determine whether it improves their asthma.

The advice people give (online) … some say ‘use it’, and others say ‘no, never, it's dangerous!’ … from what I could gather, it was clear that there is no agreed‐upon opinion ….^30^

Yeah, I turn the fan on. I sleep with the fan on. If there's a breeze blowing through the window, I don't need the fan. Then, I'm okay, but it does help. And my mom used to always tell me that coffee helped [control asthma symptoms].^19^

I tried taking a supplement I'd heard about, some grape seed extract, aloe; it seemed to help.^26^

It is important to clarify that increasing indoor ventilation by turning on a fan can reduce certain allergens.^41^ For example, when there are individuals smoking or cooking indoors, turning on an exhaust fan can reduce indoor particulate matter concentration. In such situations, the exhaust fan should be activated. However, whether fans are beneficial for asthma control remains uncertain. According to a study, grape seed extract has demonstrated potential as an antiobesity agent and a beneficial therapeutic agent with potential applications in mitigating lung tissue damage.^42^

### 3.4 Synthesised finding 2: The opportunities of people living with asthma in SDM

It is important to provide people living asthma with opportunities to participate in SDM. In the decision‐making context, healthcare professionals and medical systems are two main external determinants which can provide opportunity for asthma patients to participant SDM. This includes offering choices tailored to the needs of the patient and institutional problems in the medical system.

#### 3.4.1 Offering choices tailored to the needs of the patients

For people living with asthma, it is crucial to engage in thorough discussions to determine the pros and cons of various decisions as a prerequisite for decision‐making. Unfortunately, many patients expressed a lack of agency in decision‐making, as health care professionals did not proactively offer different choices for patients to choose and initiate thought‐provoking discussions.^15^, ^21^, ^25^, ^38^, ^39^

You sometimes feel that people are just giving you a decision but not explaining it in enough detail. [….] Even like when my mum's been there with me, it's just been, kind of […] like none of us have fully understood how I have asthma. (P/young person/2, female, 16–30, site 2)^39^

… more or less as if you're like an animal really. I mean you take an animal to the vet and he does all the work and that's what happened there(at the hospital). They do it and they never asked me what I wanted they just said ‘Oh, you've got asthma we'll cure you in fortnight’. And that was it. (PC02)^15^

Furthermore, the involvement of people living with asthma in SDM or the healthcare process is significantly constrained by their social environment, encompassing aspects such as time availability and accessibility to medical services. These factors may result in a limited opportunities for them to engage in SDM or even make it harder for them to seek medical attention, thereby exerting profound implications on their health outcomes.

I never took as much sick leave as I should have unfortunately. I left the bank with over a year's worth of sick leave accumulated. Being manager of the department, I felt I had to be there as often as possible and I know I should not have been there certain days. (Dylan, 65‐year‐old man)^16^

My daughter takes me to all my appointments. If she can't go, I can't go.^28^

#### 3.4.2 Institutional problems in the medical system

Some medical systems may lack sufficient support for SDM,^16^, ^25^, ^26^, ^27^, ^28^, ^37^, ^39^ potentially due to constraints in terms of medical insurance and medical resources patient needed. These constraints can limit the diversity of making choices. Therefore, the opportunity to provide SDM to patients is not provided.

Not a lot. I like to use generic medicines and not spend a whole lot of money. Let's start with the most reasonably priced, most effective. There are not a lot of choices.^26^

[…] how much does it cost is a valid criteria for anybody's decision‐making. (Patient 16, FG4)^40^

Sylvia ‘was to get allergy shots if insurance paid for them—the clinic was to call back with information regarding insurance approval, but hasn't’. The clinic still had not called with this information 12 months later, and the patient never had an allergy consultation.^27^

### 3.5 Synthesised finding 3: The motivation of people living with asthma in SDM

Because motivation is a cognitive process within the brain that encompasses all incentives and guiding factors behind behaviour, including goals and conscious decision‐making, we have found that factors inherent to the patients themselves may hinder their engagement in SDM.

#### 3.5.1 Emotions: Pleasant versus unpleasant experience

During face‐to‐face visits versus telephonic encounters, asthma patients described various situations they encountered with SDM.^14^, ^15^, ^16^, ^18^, ^21^, ^23^, ^25^, ^27^, ^28^, ^31^, ^37^, ^38^, ^39^ Most studies show that decision‐making experience is mainly reflected in the feelings patients experience when they communicate with medical and health professionals, including feelings regarding communication methods, attitudes and equality of decision‐making status between patients and health professionals. One of the main feeling patients have concern whether they are treated with respect by healthcare professionals. Patients judge whether they are respected by doctors' words and deeds and attach importance to the experience of ‘respect’ in decision‐making. When the experience is not good, such as when they are confronted with an arrogant attitude, misunderstanding, negation and even humiliation by doctors, patients usually choose to change doctors, interrupt medical treatment, or even respond with negative behaviours.

I'm comfortable to the point where I don't have a problem taking my medicines. If I'm not comfortable with my doctor, I'm not going to take the medicine. (Participant 1)^25^

However, when patients perceive the respect and attention of doctors, they show their trust in them in the communication process, and they are more willing to describe their asthma problems and participate in the discussion of asthma treatment, which results in a harmonious relationship between doctors and patients. Patients have a good decision‐making experience in this situation.

All doctors aren't good doctors. All doctors don't need to be doctors. But the good doctor will go in and say, okay, I know this preventative should be working. Let's go to the next step and see why it's not. Then they'll go into, are you living with pets? Do you have a dog? Are you around people that smoke? And that's your personal stuff that makes you who you are, the way you live, and some doctors—I know my doctor, let me tell you. She is awesome. (Patient Focus group #1)^38^

#### 3.5.2 Different lifestyle

Asthma, as a chronic condition, necessitates the importance of establishing harmony with the disease throughout affliction. This notion of harmony encompasses various factors such as patients' lifestyle habits, personal characteristics and pursuit of self‐worth, all of which significantly influence their treatment choices and preferences in SDM.^16^, ^25^, ^27^, ^28^

I started when I was 15, so. Uh, just farmed all me life … I'm doing what I love to do, and, uh, the alternative to that, take it away from me, and I definitely will die. So, there's no way I'm going to give up what I'm doing. (Phil, 74‐year‐old, man)^16^

#### 3.5.3 Self‐efficiency

Self‐efficiency is conceptualised as individual's belief in their capacity to organise and execute action plans to effectively manage specific situations.^43^ Self‐efficacy related to SDM encompasses not only the patients' satisfaction with their own successful asthma management but also their participation in group sharing and in sharing their successful experiences with other asthma patients, which contributes to a sense of achievement by helping others manage their asthma.^15^, ^21^, ^26^, ^30^

Erm and I like to take responsibility for my own asthma. Anyway, so I think that is important. I think more people could do that if they had the information or they knew more and it makes you feel more responsible for your life.^15^

To assure the viability of the generated evidence, the ConQual methodology^36^ was employed to evaluate the confidence level in the amalgamated results. The comprehensive ConQual summary of these findings is provided in Appendix S6.

## 4 DISCUSSION AND CONCLUSION

This is the first study to systematically identify, review and synthesise 19 studies (13 qualitative studies and six mixed studies) to explore SDM experiences among adults living with asthma. We used COM‐B model to synthesise and obtain three themes: adult asthma patients' capability, opportunities of the adult asthma patients with SDM experience, motivation of the adult asthma patients with SDM experience. Based on these themes, we have found that patient‐centred SDM can be challenging to implement. Moreover, exploring patient experiences and perspectives on SDM can provide recommendations that ensure patients are at the centre of decision‐making and inform clinical practice.

### 4.1 Discussion

This is the first systematic review and qualitative synthesis to explore the experience of adults with asthma participating in SDM. Nineteen studies of asthmatic participation in SDM were included. Three aspects provide new insights. These topics indicate the factors that promote or hinder participation in SDM, most of which can be overcome. In addition, understanding patients' experiences with SDM can help healthcare providers better design, train and implement SDM programs that are more appropriate for adults with asthma. At the same time, improve the SDM skills of healthcare professionals to help patients make better decisions in line with their preferences, so as to better manage their diseases.

#### 4.1.1 The capability of people living with asthma is the basis for implementing SDM

In our selected studies, many adult asthma patients especially female patients were likely to explicitly express their willingness to participate in decision‐making, a result that aligns with prior research findings.^44^ While some part of them were neutral or negative about participant SDM.^45^ For patients who exhibited reluctance in making decisions, our recommendation for clinicians need to maintain patient‐centred care by acknowledging and honouring the patient's preferences, and striving to offer guidance that aligns with the patient's goals and values. Because SDM does not require every patient to make a decision but rather ensures that patients are aware of the treatment they are developing with their clinician and that they have the opportunity to participate in this process.^46^ In this regard, apart from encouraging people spontaneous participate in SDM，some countries that prioritise SDM development, such as the United Kingdom, are committed to summarise the experience of various patients participating in SDM. For example, they conduct surveys on the experiences of patients participating in SDM nationwide. They established a survey website that includes various types of patients (such as pregnant women, children, etc.) participating in SDM experiences, while integrating patient experiences to prepare for the creation and optimisation of SDM information standards.^47^, ^48^ However, so far, these surveys have not conducted targeted research on people living asthma. Therefore, to enhance the intrinsic ability of people living with asthma to engage in SDM, it is highly necessary to conduct surveys on individuals living with asthma who participate in SDM, and use the information obtained to develop patient‐centred educational guidelines for SDM. Our review discovered that individual capabilities can influence asthma patients' attitudes and experiences towards SDM. The ability of participants to participate in SDM is notably correlated with their level of comprehension, assuming no intellectual impairments. A study showed that higher levels of education among patients are positively correlated with a greater inclination to actively seek information regarding their health care.^44^ The use of decision aid can help patients improve their capability to participate in SDM.^17^ In our review, many people living with asthma expressed their views on decision aid, suggesting that the design of decision support tools needs to be more concise and straightforward. Meanwhile, the types and depth of information needed by asthmatic patients are highly diverse.^49^ Diversified information creates a dilemma for patients. To accommodate all people living with asthma, it is important to enhance their' capability to assess complex information in patient SDM training.

#### 4.1.2 The most significant factor affecting patients' participation in SDM is whether asthma patients have the opportunity to participate in SDM

Most studies have indicated that patients have not been given the opportunity to engage in SDM, attributing this deficiency primarily to healthcare professionals and medical systems. Our review found that asthma patients complained about the limited communication with their clinicians and their clinicians' lack of knowledge of how to provide patients with available decision options.^15^, ^21^, ^25^, ^38^, ^39^ This perspective has been frequently mentioned as a barrier to implementing SDM for asthma care. Besides, Sapir et al.^50^ found that the primary reason for clinicians' lack of active engagement in SDM is their adherence to the traditional provider‐centric perspective. Clinicians cannot only prioritise their understanding of patients' medical conditions and history over their financial status, asthma medication adherence, lifestyle and workplace conditions. Cause these circumstances can to some extent deprive patients of their right to make certain choices, particularly in terms of economic considerations.^51^ They need to consider the aspects patient care about. Clinicians' proactive involvement in SDM should be promoted to thereby empower patients with opportunities to participate in SDM. SDM training for clinicians should mainly emphasise the positive results of SDM practice to ensure that clinicians they realise that SDM is beneficial. Meanwhile, clinicians should be directed to explain the basic principles supporting SDM, receive practical and timely feedback from their colleagues when carrying out SDM practices, and individualised training adjustments should be made according to their deficiencies to meet the specific learning requirements of these individuals.^52^

In the broader context of decision‐making, the healthcare system may also influence patients' opportunities to participate in SDM. These include a lack of continuity of treatment, short visit times and difficulty scheduling multiple visits to allow for multiple conversations.^53^ To overcome medical system barriers can enhance the patient‐centric nature of SDM, particularly since many clinicians have limited time to implement SDM, team collaboration is of paramount importance. In our review, a portion of patients expressed that they had longer interaction times with nurses.^15^, ^22^, ^39^ Lenzen et al.^54^ suggest that nurses can serve as suitable decision coaches in facilitating SDM. They can bring a complementary set of skills to those of physicians. Shade et al.^55^ found that research nurses effectively implemented and disseminated SDM as practice facilitators. Actually, since nurse is the medical staff who spends more time than clinicians with asthma patients, their participation is more conducive to the sustainable development of SDM.

#### 4.1.3 The motivation of asthma patients is the driving force that affects patient participation

Our study has revealed that self‐efficacy is a crucial influencing factor for patients' engagement in SDM and maintenance of self‐management. In this review, we found that patients with high self‐efficacy greatly enjoy the process of actively participating with their health care providers in maintaining a healthier state and that they are enthusiastic about sharing their experiences in controlling asthma with others. Patients with higher self‐perceived efficacy have a more positive attitude toward SDM, and they demonstrate increased confidence in making informed decisions.^56^ Self‐efficacy thus plays a significant role in motivating patients to participate in SDM. Therefore, when engaging with adult asthma patients in SDM, it is crucial to focus on fostering their self‐efficacy. For example, a study found that repeated acceptance of nurse education can increase self‐efficacy in patients with severe asthma.^57^

In addition to self‐efficacy, we found that the experience of asthma patients participating in SDM is influenced by their relationship with their clinician, emotional state and understanding of asthma (e.g., awareness of the side effects of drugs).^53^ Those who support SDM believe that health care decisions should respect patient values to ensure they comply with those that most people hold dear, namely, respect, trust and agency.^58^ Many studies have emphasised that good and repeated clinical interactions can accelerate the acceptance and understanding of this view among both clinicians and patients; can help people form, maintain or rebuild their self‐identity; can increase patients' trust in nurses and clinicians; and can help reduce emotional load and social stigma.^4^, ^59^, ^60^

In this study, we found that the motivations that influence patients' engagement in SDM vary and include external factors, such as unpleasant health care experiences, as well as internal factors that are amenable to intervention, such as self‐efficacy and patients' understanding of asthma. Moreover, there are situations that are difficult to intervene in, such as when patients are aware that their unhealthy lifestyle choices may adversely affect their asthma but still choose not to make any changes. A tailored approach that respects patients' preferences and addresses their specific needs should therefore be adopted after a detailed analysis of each case and must include patient‐centred communication that respects patients' autonomy while identifying and resolving any issues affecting their participation in SDM.

Metasynthesis involves the reinterpretation of research results and has many advantages and limitations. The strict inclusion criteria of this study have ensured the salience and reliability of the focal studies. However, this study does have several limitations: (1) Most of the research originated in Western countries. Thus, extrapolating these results to Eastern populations is questionable. (2) Gray literature may have been omitted from this review. (3) The data are mainly drawn from adults with asthma rather than families or health care providers.

### 4.2 Conclusion

This review has provided an in‐depth metaintegration of qualitative studies on the SDM experience of adults with asthma. To our knowledge, this is the first systematic review of the qualitative literature regarding the perception of SDM concerning people with asthma among patients. Our results have elucidated the restrictions of SDM among adults with asthma—most notably, their communication barriers with clinicians. Our findings also highlight the importance of individual differences among asthma patients and their main sources of asthma‐related information. We hope that health care providers interested in participating in SDM for asthma patients can use this work to determine what is truly needed for these patients and to facilitate the implementation and dissemination of patient‐centred care to improve patient health and treatment outcomes.

### 4.3 Practice implications

The results of this review can foster the implementation of SDM. They can help adults with asthma and enable clinicians to promote information‐sharing while emphasising patient values and preferences, to provide advice for adult asthma patients' SDM skills training, to maintain a stable cooperative relationship between themselves and their asthma patients, and help clinicians and patients reach consensus on treatment decisions to provide more personalised and tailored medical care.

## AUTHOR CONTRIBUTIONS

**Yuanyuan He**: Data curation; visualisation; software. **Xiufen Yang**: Data curation. **Jin Su**: Supervision. **Weixiang Luo**: Supervision; project administration; validation; writing—review and editing. **Qiaohong Yang**: Validation; supervision.

## CONFLICT OF INTEREST STATEMENT

The authors declare no conflict of interest.

## Supporting information

Supporting information.

Supporting information.

Supporting information.

Supporting information.

Supporting information.

Supporting information.

Supporting information.

## Data Availability

The data that supports the findings of this study are available in the Supporting Information material of this article.
