# Supplementary material for: The experience of shared decision‐making for people with asthma: A systematic review and metasynthesis of qualitative studies
Source: Health Expect. 2024 Apr 13;27(2):e14039. doi: 10.1111/hex.14039 (PMC11015866; doi:10.1111/hex.14039)
Supplement: Supplementary file 1 — Supporting information. [file HEX-27-e14039-s005.docx]

**Pubmed**

| **Searches** | **Search strategy** |  |
| --- | --- | --- |
| 1 | "Asthma"[Mesh] | 142,734 |
| 2 | Asthma[Title/Abstract] | 171,981 |
| 3 | Asthma*[Title/Abstract] | 182,481 |
| 4 | Bronchial Asthma[Title/Abstract] | 19,537 |
| 5 | #1 OR #2 OR #3 OR #4 | 202,508 |
| 6 | "Decision Making"[Mesh] | 233,054 |
| 7 | "Decision Support Techniques"[Mesh] | 82,264 |
| 8 | Decision making[Title/Abstract] | 198,010 |
| 9 | Decision support[Title/Abstract] | 22,843 |
| 10 | #6 OR #7 OR #8 OR #9 | 471,143 |
| 11 | "Professional-Patient Relations"[Mesh] | 148,495 |
| 12 | "Patient-Centered Care"[Mesh] | 24,217 |
| 13 | "Patient Participation"[Mesh] | 29,495 |
| 14 | Patient participation[Title/Abstract] | 3,847 |
| 15 | Patient engagement[Title/Abstract] | 5,001 |
| 16 | Patient involvement[Title/Abstract] | 3,682 |
| 17 | Client participation[Title/Abstract] | 138 |
| 18 | Client engagement[Title/Abstract] | 245 |
| 19 | Client involvement[Title/Abstract] | 98 |
| 20 | Patient relation*[Title/Abstract] | 27,224 |
| 21 | Patient preference*[Title/Abstract] | 12,125 |
| 22 | Patient centered[Title/Abstract] | 24,804 |
| 23 | Patient centred[Title/Abstract] | 8,941 |
| 24 | #11 OR #12 OR #13 OR #14 OR #15 OR #16 OR #17 OR #18 OR #19 OR #20 OR #21 OR #22 OR #23 | 243,040 |
| 25 | aid*[Title/Abstract] | 211,556 |
| 26 | tool*[Title/Abstract] | 990,397 |
| 27 | box*[Title/Abstract] | 97,170 |
| 28 | "Decision Making, Shared"[Mesh] | 1,972 |
| 29 | Shared decision*[Title/Abstract] | 15,028 |
| 30 | Informed decision*[Title/Abstract] | 10,771 |
| 31 | Informed choice*[Title/Abstract] | 3,171 |
| 32 | Collaborative decision*[Title/Abstract] | 488 |
| 33 | Issue card*[Title/Abstract] | 6 |
| 34 | Patient material*[Title/Abstract] | 1,714 |
| 35 | Patient education material*[Title/Abstract] | 1,176 |
| 36 | Patient tool*[Title/Abstract] | 55 |
| 37 | Patient handout*[Title/Abstract] | 101 |
| 38 | Question prompt list*[Title/Abstract] | 189 |
| 39 | Pamphlet*[Title/Abstract] | 2,401 |
| 40 | Handout*[Title/Abstract] | 1,507 |
| 41 | Educational material*[Title/Abstract] | 5,543 |
| 42 | Patient Education as Topic | 106,612 |
| 43 | Patient Education Handout[Title/Abstract] | 76 |
| 45 | "Consumer Health Information"[Mesh] | 13,447 |
| 46 | "Pamphlets"[Mesh] | 4,130 |
| 47 | #25 OR #26 OR #27 OR #28 OR #29 OR #30 OR #31 OR #32 OR #33 OR #34 OR #35 OR #36 OR #37 OR #38 OR #39 OR #40 OR #41 OR #42 OR #43 OR #44 OR #45 OR #46 | 1,414,718 |
| 48 | "Qualitative Research"[Mesh] | 82,973 |
| 49 | qualitative research[Title/Abstract] | 35,176 |
| 50 | interview*[Title/Abstract] | 458,531 |
| 51 | experience*[Title/Abstract] | 1,364,802 |
| 52 | qualitative*[Title/Abstract] | 382,623 |
| 53 | phenomenon*[Title/Abstract] | 251,469 |
| 54 | narrative[Title/Abstract] | 64,163 |
| 55 | ground theory[Title/Abstract] | 46 |
| 56 | Ethnograph*[Title/Abstract] | 14,123 |
| 57 | Narration*[Title/Abstract] | 1,175 |
| 58 | #48 OR #49 OR #50 OR #51 OR #52 OR #55 OR #56 OR #57 | 2,204,942 |
| 60 | #5 AND #10 AND #24 AND #47 AND #58 | 113 |

**Web Of Science**

| **Searches** | **Search strategy** |  |
| --- | --- | --- |
| 1 | TS=(Asthma) | 310163 |
| 2 | TS=(Asthma*) | 318262 |
| 3 | TS=(Bronchial Asthma) | 62946 |
| 4 | #1 OR #2 OR #3 | 318262 |
| 5 | TS=(Decision Making) | 1112081 |
| 6 | TS=(Decision support techniques) | 136644 |
| 7 | TS=(Decision support) | 419915 |
| 8 | #7 OR #6 OR #5 | 1289402 |
| 9 | TS=(Patient preference) | 89547 |
| 10 | TS=(Patient-Centered Care) | 48647 |
| 11 | TS=(Patient Participation) | 111107 |
| 13 | TS=(Patient engagement) | 42423 |
| 14 | TS=(Patient involvement) | 317942 |
| 15 | TS=(Client participation) | 8233 |
| 16 | TS=(Client engagement) | 6154 |
| 17 | TS=(Client involvement) | 5124 |
| 18 | TS=(Patient relation*) | 1181236 |
| 19 | TS=(Patient preference*) | 89562 |
| 20 | TS=(Patient centered) | 886981 |
| 21 | TS=(Patient centred) | 886981 |
| 22 | TS=(Decision) | 1879540 |
| 23 | #9 OR #10 OR #11 OR #12 OR #13 OR #14 OR #15 OR #16 OR #17 OR #18 OR #19 OR #20 OR #21 OR #22 | 4140048 |
| 24 | TS=(aid*) | 1376183 |
| 25 | TS=(tool*) | 2908387 |
| 26 | TS=(box*) | 386234 |
| 27 | TS=(Decision Making, Shared) | 77142 |
| 28 | TS=(Shared decision*) | 109413 |
| 29 | TS=(Informed decision*) | 122368 |
| 30 | TS=(Informed choice*) | 40431 |
| 31 | TS=( Collaborative decision*) | 27020 |
| 32 | TS=(Issue card*) | 78602 |
| 33 | TS=(Patient material*) | 1090482 |
| 34 | TS=(Patient education material*) | 31207 |
| 35 | TS=(Patient tool*) | 465208 |
| 36 | TS=( Patient handout*) | 1156 |
| 37 | TS=(Question prompt list*) | 953 |
| 38 | TS=(Brochure*) | 6127 |
| 39 | TS=(Pamphlet*) | 11801 |
| 40 | TS=(Handout*) | 3250 |
| 41 | TS=( Educational material*) | 81133 |
| 42 | TS=(Patient Education as Topic) | 117084 |
| 43 | TS=(Patient Education Handout) | 781 |
| 44 | TS=(Consumer Health Information) | 38356 |
| 45 | TS=(Pamphlets) | 11527 |
| 46 | #24 OR #25 OR #26 OR #27 OR #28 OR #29 OR #30 OR #31 OR #32 OR #33 OR #34 OR #35 OR #36 OR #37 OR #38 OR #39 OR #40 OR #41 OR #42 OR #43 OR #44 OR #45 | 5935282 |
| 47 | #4 AND #8 AND #23 AND #46 | 1281 |

*TS=Theme subject

**MEDLINE**

| **Searches** | **Search strategy** |  |
| --- | --- | --- |
| 1 | TS=(Asthma) | 198811 |
| 2 | TS=(Asthma*) | 203420 |
| 3 | TS=(Bronchial Asthma) | 40573 |
| 4 | #1 OR #2 OR #3 | 203420 |
| 5 | TS=(Decision Making) | 358509 |
| 6 | TS=(Decision support techniques) | 31725 |
| 7 | TS=(Decision support) | 132377 |
| 8 | #7 OR #6 OR #5 | 417228 |
| 9 | TS=(Patient preference) | 63655 |
| 10 | TS=(Patient-Centered Care) | 40965 |
| 11 | TS=(Patient Participation) | 85833 |
| 13 | TS=(Patient engagement) | 30790 |
| 14 | TS=(Patient involvement) | 236967 |
| 15 | TS=(Client participation) | 3243 |
| 16 | TS=(Client engagement) | 2197 |
| 17 | TS=(Client involvement) | 1801 |
| 18 | TS=(Patient relation*) | 908028 |
| 19 | TS=(Patient preference*) | 63664 |
| 20 | TS=(Patient centered) | 678261 |
| 21 | TS=(Patient centred) | 678261 |
| 22 | TS=(Decision) | 586999 |
| 23 | #9 OR #10 OR #11 OR #12 OR #13 OR #14 OR #15 OR #16 OR #17 OR #18 OR #19 OR #20 OR #21 OR #22 | 2311248 |
| 24 | TS=(aid*) | 499413 |
| 25 | TS=(tool*) | 990469 |
| 26 | TS=(box*) | 136596 |
| 27 | TS=(Decision Making, Shared) | 27162 |
| 28 | TS=(Shared decision*) | 33079 |
| 29 | TS=(Informed decision*) | 17631 |
| 30 | TS=(Informed choice*) | 17631 |
| 31 | TS=( Collaborative decision*) | 6104 |
| 32 | TS=(Issue card*) | 34912 |
| 33 | TS=(Patient material*) | 530915 |
| 34 | TS=(Patient education material*) | 21979 |
| 35 | TS=(Patient tool*) | 324214 |
| 36 | TS=( Patient handout*) | 834 |
| 37 | TS=(Question prompt list*) | 416 |
| 38 | TS=(Brochure*) | 2805 |
| 39 | TS=(Pamphlet*) | 6013 |
| 40 | TS=(Handout*) | 1444 |
| 41 | TS=( Educational material*) | 24138 |
| 42 | TS=(Patient Education as Topic) | 111384 |
| 43 | TS=(Patient Education Handout) | 556 |
| 44 | TS=(Consumer Health Information) | 17752 |
| 45 | TS=(Pamphlets) | 6005 |
| 46 | #24 OR #25 OR #26 OR #27 OR #28 OR #29 OR #30 OR #31 OR #32 OR #33 OR #34 OR #35 OR #36 OR #37 OR #38 OR #39 OR #40 OR #41 OR #42 OR #43 OR #44 OR #45 | 2311783 |
| 47 | #4 AND #8 AND #23 AND #46 | 772 |

*TS=Theme subject

**SSCI**

| **Searches** | **Search strategy** |  |
| --- | --- | --- |
| 1 | TS=(Asthma) | 16476 |
| 2 | TS=(Asthma*) | 16951 |
| 3 | TS=(Bronchial Asthma) | 589 |
| 4 | #1 OR #2 OR #3 | 16951 |
| 5 | TS=(Decision Making) | 265094 |
| 6 | TS=(Decision support techniques) | 6138 |
| 7 | TS=(Decision support) | 95060 |
| 8 | #7 OR #6 OR #5 | 302577 |
| 9 | TS=(Patient preference) | 22867 |
| 10 | TS=(Patient-Centered Care) | 13351 |
| 11 | TS=(Patient Participation) | 22427 |
| 13 | TS=(Patient engagement) | 15014 |
| 14 | TS=(Patient involvement) | 18608 |
| 15 | TS=(Client participation) | 3128 |
| 16 | TS=(Client engagement) | 3168 |
| 17 | TS=(Client involvement) | 2055 |
| 18 | TS=(Patient relation*) | 113239 |
| 19 | TS=(Patient preference*) | 22870 |
| 20 | TS=(Patient centered) | 76555 |
| 21 | TS=(Patient centred) | 76555 |
| 22 | #9 OR #10 OR #11 OR #12 OR #13 OR #14 OR #15 OR #16 OR #17 OR #18 OR #19 OR #20 | 229747 |
| 23 | TS=(aid*) | 126147 |
| 24 | TS=(tool*) | 273400 |
| 25 | TS=(box*) | 16956 |
| 26 | TS=(Decision Making, Shared) | 22787 |
| 27 | TS=(Shared decision*) | 32796 |
| 28 | TS=(Informed decision*) | 35860 |
| 29 | TS=(Informed choice*) | 12164 |
| 30 | TS=( Collaborative decision*) | 6898 |
| 31 | TS=(Issue card*) | 5702 |
| 32 | TS=(Patient education material*) | 3987 |
| 33 | TS=(Patient tool*) | 48961 |
| 34 | TS=( Patient handout*) | 270 |
| 35 | TS=(Question prompt list*) | 330 |
| 36 | TS=(Brochure*) | 1623 |
| 37 | TS=(Pamphlet*) | 1404 |
| 38 | TS=(Handout*) | 745 |
| 39 | TS=( Educational material*) | 9847 |
| 40 | TS=(Patient Education as Topic) | 1930 |
| 41 | TS=(Patient Education Handout) | 161 |
| 42 | TS=(Consumer Health Information) | 8482 |
| 43 | TS=(Pamphlets) | 1374 |
| 44 | #22 OR #23 OR #24 OR #25 OR #26 OR #27 OR #28 OR #29 OR #30 OR #31 OR #32 OR #33 OR #34 OR #35 OR #36 OR #37 OR #38 OR #39 OR #40 OR #41 OR #42 | 495498 |
| 45 | #4 AND #8 AND #22 AND #43 | 112 |

*TS=Theme subject

**Embase**

| **Searches** | **Search strategy** |  |
| --- | --- | --- |
| 1 | 'asthma'/exp | 323214 |
| 2 | 'asthma*':ab,ti | 278134 |
| 3 | 'bronchial asthma':ab,ti | 30128 |
| 4 | #1 OR #2 OR #3 | 360772 |
| 5 | 'decision making'/exp | 472492 |
| 6 | 'decision support techniques':ab,ti | 25 |
| 7 | 'decision making':ab,ti | 2258606 |
| 8 | 'decision support':ab,ti | 26608 |
| 9 | #5 OR #6 OR #7 OR #8 | 578612 |
| 10 | 'patient preference'/exp | 26427 |
| 11 | 'patient participation'/exp | 35567 |
| 13 | 'patient-centered':ab,ti | 30981 |
| 14 | 'patient preference*':ab,ti | 17583 |
| 15 | 'patient centered':ab,ti | 30971 |
| 16 | 'patient centred':ab,ti | 11389 |
| 17 | 'decision':ab,ti | 515429 |
| 18 | 'patient participation':ab,ti | 3879 |
| 19 | 'patient engagement':ab,ti | 6149 |
| 20 | 'patient involvement':ab,ti | 4397 |
| 21 | 'client participation':ab,ti | 175 |
| 22 | 'client engagement':ab,ti | 291 |
| 23 | 'client involvement':ab,ti | 104 |
| 24 | 'patient relation*':ab,ti | 13165 |
| 25 | #10 OR #11 OR #12 OR #13 OR #14 OR #15 OR #16 OR #17 OR #18 OR #19 OR #20 OR #21 | 612258 |
| 26 | 'decision making, shared'/exp | 14884 |
| 27 | 'patient education'/exp | 125196 |
| 28 | 'consumer health information'/exp | 4266 |
| 29 | 'pamphlets'/exp | 202288 |
| 30 | 'shared decision*':ab,ti | 19754 |
| 31 | 'informed decision*':ab,ti | 14255 |
| 32 | 'informed choice*':ab,ti | 3982 |
| 33 | 'collaborative decision*':ab,ti | 614 |
| 34 | 'patient education material':ab,ti | 309 |
| 35 | 'educational material*':ab,ti | 8876 |
| 36 | 'patient education handout':ab,ti | 45 |
| 37 | 'question prompt list*':ab,ti | 256 |
| 38 | 'pamphlet*':ab,ti | 3400 |
| 39 | 'handout*':ab,ti | 3103 |
| 40 | 'brochure*':ab,ti | 4767 |
| 41 | 'patient handout*':ab,ti | 218 |
| 42 | 'patient tool*':ab,ti | 99 |
| 43 | 'patient material*':ab,ti | 3891 |
| 44 | 'issue card*':ab,ti | 62 |
| 45 | 'aid*':ab,ti | 560291 |
| 46 | 'tool*':ab,ti | 1314491 |
| 47 | 'box*':ab,ti | 136630 |
| 48 | #25 OR #26 OR #27 OR #28 OR #29 OR #30 OR #31 OR #32 OR #33 OR #34 OR #35 OR #36 OR #37 OR #38 OR #39 OR #40 OR #41 OR #42 OR #43 OR #44 | 939006 |
| 49 | #4 AND #9 AND #24 AND #47 | 513 |

*exp=explode, ab,ti=Article title, Abstract

**ProQuest**

| **Searches** | **Search strategy** |  |
| --- | --- | --- |
| 1 | TS=(Asthma) | 7005 |
| 2 | TS=(Asthma*) | 7411 |
| 3 | TS=(Bronchial Asthma) | 836 |
| 4 | #1 OR #2 OR #3 | 7411 |
| 5 | TS=(Decision Making) | 173374 |
| 6 | TS=(Decision support techniques) | 8316 |
| 7 | TS=(Decision support) | 66930 |
| 8 | #7 OR #6 OR #5 | 194793 |
| 9 | TS=(Patient preference) | 3362 |
| 10 | TS=(Patient-Centered Care) | 1751 |
| 11 | TS=(Patient Participation) | 4404 |
| 13 | TS=(Patient engagement) | 2657 |
| 14 | TS=(Patient involvement) | 6262 |
| 15 | TS=(Client participation) | 2009 |
| 16 | TS=(Client engagement) | 1403 |
| 17 | TS=(Client involvement) | 1496 |
| 18 | TS=(Patient relation*) | 47389 |
| 19 | TS=(Patient preference*) | 3363 |
| 20 | TS=(Patient centered) | 19289 |
| 21 | TS=(Patient centred) | 19289 |
| 22 | TS=(Decision) | 261844 |
| 23 | #9 OR #10 OR #11 OR #12 OR #13 OR #14 OR #15 OR #16 OR #17 OR #18 OR #19 OR #20 OR #21 OR #22 | 330045 |
| 24 | TS=(aid*) | 127832 |
| 25 | TS=(tool*) | 300369 |
| 26 | TS=(box*) | 25574 |
| 27 | TS=(Decision Making, Shared) | 16582 |
| 28 | TS=(Shared decision*) | 22924 |
| 29 | TS=(Informed decision*) | 19197 |
| 30 | TS=(Informed choice*) | 7086 |
| 31 | TS=( Collaborative decision*) | 5004 |
| 32 | TS=(Issue card*) | 4625 |
| 33 | TS=(Patient material*) | 12252 |
| 34 | TS=(Patient education material*) | 1245 |
| 35 | TS=(Patient tool*) | 19264 |
| 36 | TS=( Patient handout*) | 133 |
| 37 | TS=(Question prompt list*) | 280 |
| 38 | TS=(Brochure*) | 1323 |
| 39 | TS=(Pamphlet*) | 2932 |
| 40 | TS=(Handout*) | 821 |
| 41 | TS=( Educational material*) | 821 |
| 42 | TS=(Patient Education as Topic) | 738 |
| 43 | TS=(Patient Education Handout) | 102 |
| 44 | TS=(Consumer Health Information) | 3151 |
| 45 | TS=(Pamphlets) | 2800 |
| 46 | #24 OR #25 OR #26 OR #27 OR #28 OR #29 OR #30 OR #31 OR #32 OR #33 OR #34 OR #35 OR #36 OR #37 OR #38 OR #39 OR #40 OR #41 OR #42 OR #43 OR #44 OR #45 | 510254 |
| 47 | #4 AND #8 AND #23 AND #46 | 78 |

*TS=Theme subject

**CINAHL**

| **Searches** | **Search strategy** |  |
| --- | --- | --- |
| S1 | SU Asthma OR TX Asthma OR TX Asthma* OR TX Bronchial Asthma | 91801 |
| S2 | SU Decision Making OR TX Decision support techniques OR TX Decision making OR TX Decision support OR TX Decision making | 423869 |
| S3 | SU Patient preference OR TX Patient-Centered Care OR TX Patient preference* OR TX Patient centered OR TX Patient centred OR TX Decision | 687468 |
| S4 | SU Patient Participation OR TX Patient participation OR TX Patient engagement OR TX Patient involvement OR TX Client participation OR TX Client engagement OR TX Client involvement OR TX Patient relation* | 214633 |
| S5 | S3 OR S4 | 831939 |
| S6 | SU Decision Making, Shared OR TX Shared decision* OR TX Informed decision* OR TX Informed choice* OR TX Collaborative decision* | 51196 |
| S7 | SU Patient Education as Topic OR TX Patient education material OR TX Educational material* OR TX Patient Education Handout OR TX Question prompt list* | 16546 |
| S8 | SU Consumer Health Information OR SU Pamphlets OR TX Pamphlet* OR TX Handout* OR TX Brochure* OR TX Patient handout* OR TX Patient tool* OR TX Patient material* OR TX Issue card* OR TX aid* OR TX tool* OR TX box* | 1102841 |
| S9 | S6 OR S7 OR S8 | 1131131 |
| S10 | S1 AND S2 AND S5 AND S9 | 1200 |

*SU=Subject unit, TX=All Text

**The Cochrane library**

| **Searches** | **Search strategy** |  |
| --- | --- | --- |
| #1 | MeSH descriptor: [Asthma] | 15019 |
| #2 | (Asthma):ti,ab, | 35075 |
| #3 | (Asthma*):ti,ab, | 37432 |
| #4 | (Bronchial Asthma):ti,ab, | 6233 |
| #5 | #1 OR #2 OR #3 OR $4 | 967930 |
| #6 | MeSH descriptor: [Decision Making] | 6758 |
| #7 | MeSH descriptor: [Decision Support Techniques] | 3983 |
| #8 | (Decision making):ti,ab, | 21517 |
| #9 | (Decision makingDecision support):ti,ab, | 2 |
| #10 | #6 OR #7 OR #8 OR #9 | 27331 |
| #11 | MeSH descriptor: [Patient Preference] | 1217 |
| #12 | MeSH descriptor: [Patient-Centered Care] | 1042 |
| #13 | MeSH descriptor: [Patient Participation] | 2063 |
| #14 | (Patient engagement):ti,ab, | 12559 |
| #15 | (Patient involvement):ti,ab, | 66740 |
| #16 | (Client participation):ti,ab, | 3820 |
| #17 | (Client engagement):ti,ab, | 886 |
| #18 | (Client involvement):ti,ab, | 932 |
| #19 | (Patient relation*):ti,ab, | 82008 |
| #20 | (Patient preference*):ti,ab, | 13669 |
| #21 | (Patient centered):ti,ab, | 123846 |
| #22 | (Patient centred):ti,ab, | 123846 |
| #23 | (Decision):ti,ab, | 40127 |
| #24 | #11 OR #12 OR #13 OR #14 OR #15 OR #16 OR #17 OR #18 OR #19 OR #20 OR #21 OR #22 OR #23 | 294886 |
| #25 | (aid*):ti,ab, | 28104 |
| #26 | (tool*):ti,ab, | 47582 |
| #27 | (box*):ti,ab, | 5101 |
| #28 | MeSH descriptor: [Decision Making, Shared] | 144 |
| #29 | (Shared decision*):ti,ab, | 3151 |
| #30 | (Informed decision*):ti,ab, | 13824 |
| #31 | (Informed choice*):ti,ab, | 6888 |
| #32 | (Collaborative decision*):ti,ab, | 1381 |
| #33 | (Issue card*):ti,ab, | 3764 |
| #34 | (Patient material*):ti,ab, | 73224 |
| #35 | (Patient education material*):ti,ab, | 5520 |
| #36 | (Patient tool*):ti,ab, | 30129 |
| #37 | (Patient handout*):ti,ab, | 537 |
| #38 | (Question prompt list*):ti,ab, | 292 |
| #39 | (Brochure*):ti,ab, | 2025 |
| #40 | (Pamphlet*):ti,ab, | 2879 |
| #41 | (Handout*):ti,ab, | 934 |
| #42 | MeSH descriptor: [Patient Education as Topic] | 10090 |
| #43 | (Patient Education Handout):ti,ab, | 308 |
| #44 | MeSH descriptor: [Consumer Health Information] | 884 |
| #45 | MeSH descriptor: [Pamphlets] | 987 |
| #46 | #25 OR #26 #27 OR #28 OR #29 OR #30 OR #31 OR #31 OR #32 OR #33 OR #34 OR #35 OR #36 OR #37 OR #38 OR #39 OR #40 OR #41 OR #42 OR #43 OR #44 OR #45 | 156888 |
| #47 | #5 AND #10 AND # 24 AND #46 | 1578 |

*Ti,ab=Title OR Abstract

**Psychology and Behavioral Science Collection**

| **Searches** | **Search strategy** |  |
| --- | --- | --- |
| S1 | SU Asthma OR TX Asthma OR TX Asthma* OR TX Bronchial Asthma | 22204 |
| S2 | SU Decision Making OR SU Decision support techniques OR TX Decision making OR TX Decision support | 121944 |
| S3 | SU Patient preference OR SU Patient-Centered Care OR TX Patient preference* OR TX Patient centered OR TX Patient centred OR Decision | 59276 |
| S4 | SU Patient Participation OR TX Patient participation OR TX Patient engagement OR TX Patient involvement OR TX Client participation OR TX Client engagement OR TX Client involvement OR TX Patient relation* | 52683 |
| S5 | S3 OR S4 | 102893 |
| S6 | SU Decision Making, Shared OR TX Shared decision* OR TX Informed decision* OR TX Informed choice* OR TX Collaborative decision* | 19025 |
| S7 | SU Patient Education as Topic OR TX Patient education material OR TX Educational material* OR TX Patient Education Handout OR TX Question prompt list* | 6210 |
| S8 | SU Consumer Health Information OR SU Pamphlets OR TX Pamphlet* OR TX Handout* OR TX Brochure* OR TX Patient handout* OR TX Patient tool* OR TX Patient material* OR TX Issue card* OR TX aid* OR tool* OR box* | 224578 |
| S9 | S6 OR S7 OR S8 | 238545 |
| S10 | S1 AND S2 AND S5 AND S9 | 802 |

*SU=Subject unit, TX=All Text

**APA PsycINFO**

| **Searches** | **Search strategy** |  |
| --- | --- | --- |
| S1 | SU Asthma OR TX Asthma OR TX Asthma* OR TX Bronchial Asthma | 9167 |
| S2 | SU Decision Making OR SU Decision support techniques OR TX Decision making OR TX Decision support | 168246 |
| S3 | SU Patient preference OR SU Patient-Centered Care OR TX Patient preference* OR TX Patient centered OR TX Patient centred OR TX Decision | 293322 |
| S4 | SU Patient Participation OR TX Patient participation OR TX Patient engagement OR TX Patient involvement OR TX Client participation OR TX Client engagement OR TX Client involvement OR TX Patient relation* | 68881 |
| S5 | S3 OR S4 | 350813 |
| S6 | SU Decision Making, Shared OR TX Shared decision* OR TX Informed decision* OR TX Informed choice* OR TX Collaborative decision* | 12650 |
| S7 | SU Patient Education as Topic OR TX Patient education material* OR TX Question prompt list* OR TX Educational material* OR TX Patient Education Handout | 13995 |
| S8 | SU Consumer Health Information OR SU Pamphlets OR TX Pamphlet* OR TX Handout* OR TX Brochure* OR TX Patient handout* OR TX Patient tool* OR TX Patient material* OR TX Issue card* OR TX aid* OR TX tool* OR TX box* | 521770 |
| S9 | S6 OR S7 OR S8 OR S9 | 541169 |
| S10 | (S6 OR S7 OR S8 OR S9) AND (S1 AND S2 AND S5 AND S10) | 91 |

*SU=Subject unit, TX=All Text
