# Supplementary material for: The experience of shared decision‐making for people with asthma: A systematic review and metasynthesis of qualitative studies
Source: Health Expect. 2024 Apr 13;27(2):e14039. doi: 10.1111/hex.14039 (PMC11015866; doi:10.1111/hex.14039)
Supplement: Supplementary file 2 — Supporting information. [file HEX-27-e14039-s004.docx]

**Appendix Ⅱ：List of excluded studies**

|  | **Study** | **Reasons for exclusion** |
| --- | --- | --- |
| 1 | Jahedi, Downie et al. 2017. Inhaler Technique in Asthma: How Does It Relate to Patients' Preferences and Attitudes Toward Their Inhalers? | This research content just a little part about communication between patients and doctors |
| 2 | Salandi, Vu-Eickmann et al. 2023. Implementing the Patient Needs in Asthma Treatment (NEAT) questionnaire in routine care: a qualitative study among patients and health professionals | The article does not contain any information related to shared-decision making. |
| 3 | O'Connell, McCarthy et al. 2021.The preferences of people with asthma or chronic obstructive pulmonary disease for self-management support: A qualitative descriptive study | This research content just a little part about communication between patients and doctors |
| 4 | Lenzen, Daniëls et al. 2018. What makes it so difficult for nurses to coach patients in shared decision making? A process evaluation | The object of this research is about nurses |
| 5 | Canny,Donaghy et al. 2023. Long-term effects of negotiated treatment plans on self-management behaviors and satisfaction with care among women with asthma. | The research does not contain any qualitative study. |
| 6 | Ludden, Shade et al. 2019. Asthma dissemination around patient-centered treatments in North Carolina (ADAPT-NC): a cluster randomized control trial evaluating dissemination of an evidence-based shared decision-making intervention for asthma management | The research does not contain any qualitative study. |
| 7 | Gagné , Légaré et al. 2016.  Development of a patient decision aid on inhaled corticosteroids use for adults with asthma | The reserch aim is about the development of the decision aid, there is some qualitative information but is not available. |
| 8 | Reddel, Correll et al. 2019 Patient decision-making around use of reliever inhalers in mild asthma | Just patient decision-making, it doesn’t mention about doctor、nurse and so on. |
| 9 | Shade , Ludden  et al. 2021 Development of a patient decision aid on inhaled corticosteroids use for adults with asthma | Just simple dialogue, inconsistent with qualitative research methods. |
| 10 | Davis，Tudball et al.2019 "You've got to breathe, you know" - asthma patients and carers' perceptions around purchase and use of asthma preventer medicines. | The data includes adults and carers for children with asthma. But the relative part doesn’t include adult patients’ opinions. |
| 11 | George M, Pantalon MV, Sommers MLS, Glanz K, Jia H, Chung A, Norful AA, Poghosyan L, Coleman D, Bruzzese JM. Shared decision-making in the BREATHE asthma intervention trial: A research protocol. | The randomized controlled trial contain qualitative part about shared-decision making,but the detail of the information  is unavailable  is |
| 12 | Jørgensen V, Launsø L. Patients' choice of asthma and allergy treatments. | It is an explorative study with semi-structured interviews, but just a few sentences said by asthma patients. |
| 13 | Kielmann T, Huby G, Powell A, Sheikh A, Price D, Williams S, Pinnock H. From awareness to involvement? A qualitative study of respiratory patients' awareness of health service change. | The population was people with respiratory diseases, there were words that included people with asthma, but there was no element of shared decision-making |
| 14 | Lu, E, Solovyeva, K.Hebert, Z. Kietzer, L. Griffiths, S. Antoun, Z. E. Keeley, T. Alfonso-Cristancho, R. Shared decision making in severe asthma therapy: qualitative study of physician-patient communication | This is an abstract of the meeting. There is no way to extract the qualitative research |
| 15 | Upton J, Fletcher M, Madoc-Sutton H, Sheikh A, Caress AL, Walker S. Shared decision making or paternalism in nursing consultations? A qualitative study of primary care asthma nurses' views on sharing decisions with patients regarding inhaler device selection. | There was only a perception of nurses making shared decisions about patients, not patients |
| 16 | Palmsten K, Bredesen D, JaKa MM, Kumar PC, Ziegenfuss JY, Kharbanda EO. "I know my body better than you:" patient focus groups to inform a decision aid on oral corticosteroid use during pregnancy. Pharmacoepidemiol Drug Saf. 2021 Apr;30(4):451-461. | Considering the significant differences between the pregnant population and adults, we have decided to exclude this article. |
| 17 | Tapp H, Derkowski D, Calvert M, Welch M, Spencer S. Patient perspectives on engagement in shared decision-making for asthma care. Fam Pract. 2017 Jun 1;34 (3):353-357. | The patient means child not adult, so it is out of place in our review. |
