# Supplementary material for: The experience of shared decision‐making for people with asthma: A systematic review and metasynthesis of qualitative studies
Source: Health Expect. 2024 Apr 13;27(2):e14039. doi: 10.1111/hex.14039 (PMC11015866; doi:10.1111/hex.14039)
Supplement: Supplementary file 3 — Supporting information. [file HEX-27-e14039-s007.docx]

**Appendix Ⅲ: QARI data extraction of included studies**

| **Author (First)** | **Methodology** | **Methods** | **Platform/Length of interview** | **Country** | **Population** | **Gender of patients (N. male/female)** | **Aim** | **Place of data collection** | - **Result Theme（Circle represent hemes and bracket represent subthemes）** |
| --- | --- | --- | --- | --- | --- | --- | --- | --- | --- |
| Alzayer et al. 2002[1] | Q | Qualitative approach and a  semi-structured interviews | Face-to-face interviews/25 min | Saudi Arabia | Asthma | N=23(4/19) | The aim of this study was to explore the experience of  Saudi participants in managing their asthma and their perspectives  about using future pharmacy-based services for asthma  management. | At clinic or community pharmacy or a café in the selected shopping malls | ①**Participants experience of asthma;**  ②**Participants’ beliefs and perceptions about health and medicines:**   1. Asthma literacy and information needs; 2. Beliefs in alternative medicine systems |
| Canny et al. 2023[2] | Q | Qualitative approach and a semi-structured interviews | One-on-one telephone interviews/30-45 min | England | Asthma | N=17(5/12) | To explore patient experiences relating to their asthma diagnosis and to understand how a CDSS could be used to improve the diagnostic process for patients. | Telephone | **①Diagnosis: The patient experience;**   1. Knowledge and understanding of asthma 2. Communication 3. Receiving and retaining information 4. Self‐management   **②CDSS: Patient experience and views**   1. Patient experiences of screen sharing 2. Online health information use 3. Patient views on an asthma CDSS 4. Barriers and facilitators to a CDSS being used |
| Caress et al. 2002 [3] | Q | Tape-recorded focused-conversation style interviews.  Interview topic guide derived from the literature. Sort cards  employed to provide the focus for exploration of role preferences. | Face-to-face interviews/NA | England | Asthma | N=32(17/15) | To explore preferred treatment decision-making roles, and rationales for role preference, and to identify perceived facilitators to and barriers from attaining preferred role. | At home | **①Rationales for role preference:**  (1)Patient’s level of knowledge；  (2)Trust in health professionals and in efficacy of treatment；  (3)Length of time with condition；  (4)Severity of condition at decisional juncture；  (5)Lifelong nature of asthma；  (6)Perception that “It is my body”；  (7)Characteristics of the individual；  (8)Patient’s response to health professionals；  **②Other considerations:**  (9) Specialism vs. generalism  (10) The role of health professionals other than clinicians |
| Eassey et al. 2019 [4] | Q | In‐depth semi‐structured interviews that were video‐ and/or audio‐recorded.  and transcribed. Qualitative interview approach | Face-to-face interviews/1.5-4 h | Australia | Severe Asthma | N=29(14/15) | To explore the role of autonomy in patients’ narratives about their experiences of living with and managing severe asthma. | In the respondents’  homes, or elsewhere. | **①The desire to live an “unconstrained” life**   1. Health‐care interactions 2. Employment   **②Preservation of self‐identity**   1. Maintaining valued roles 2. Searching for normality |
| Gagné et al. 2022[5] | M | A list of 15 critical issues were identified in focus groups and interviews.  Verbatim transcripts were imported into MAXQDA 2020. MG read and coded transcripts line by line. Qualitative content analysis | Virtual interviews/NA | Canada | Mild asthma | N=21(9/12) | To develop an electronic decision aid to guide discussions about the pros and cons of treatment, and to identify and integrate user preferences. | Online | 1. Content Preferences 2. Format Preferences 3. Preferences for Process |
| George et al. 2015[6] | Q | Qualitative approach and semi-structured open-ended interviews | Face-to-face interviews/20-40 min | America | Asthma | N=35(10/15) | To identify urban adults’ perceptions of facilitators and barriers to asthma control,  including the role of self-care, medications, environmental trigger remediation, and primary  care. | At home | ①Monitoring and responding to deteriorating control  ②Beliefs about ICS and SABAs  ③Triggers avoidance/remediation  ④Role of primary care |
| George et al. 2016[7] | Q | A qualitative analysis of transcripts from 33 audio-recorded primary care visits using conventional content analysis techniques. | Face-to-face interviews/9-50 min | America | Asthma | N=33(1/32) | To explore whether patients’ personal beliefs about inhaled corticosteroid (ICS) and integrative medicine (IM) are discussed at routine primary care visits for asthma. | At clinic | ①Negative ICS beliefs  ②IM use for asthma  ③Decision-making  ④Healthy lifestyles |
| George et al. 2020 [8] | Q | Qualitative descriptive methodology that guided the design and the conduct of focus groups | Focus group interviews/2.5-3 h | America | Asthma | N=32(NA) | To understand how ICS nonadherence could be addressed from the perspective of AA adults with asthma, their family, and friends | Two urban federally qualified health centers | ①To be Heard and Respected  ②Wish to Receive Patient‑Centered Care  ③Underscore the Risk of Inhaled Corticosteroid  ④Non‑Adherence |
| Hannane et al. 2019 [9] | Q | Unstructured interviews. Grounded-theory approach. Qualitative content analysis | Face-to-face interviews/12-60 min | France | Asthma | N=30(12/18) | To explore the perceptions of French adult asthma patients regarding their care pathway | In GP clinic | ①The stakeholders of patients  ②Patient relationships with healthcare professionals  ③Interprofessional collaboration |
| Hoskins et al. 2016 [10] | M | Individual semistructured interviews, analyzed following the guidelines for thematic framework analysis. | Face-to-face interviews (telephone interviews were offered as an option)/30-40 min | England | Active asthma | N=14(7/7) | To explore (1)  the experience, acceptability, and perceived usefulness of the GOAL tool and goal-setting process; (2) the perceived impact  of the intervention on self-management, quality of life and  clinical practice; (3) the perceived change in professional-  patient communication; and (4) experiences and accept-ability of all elements of the trial. | NA | ①Coherence: Meaning and  sense making by participants.  ②Cognitive participation:  Commitment and  engagement by participants  ③Collective action: The work participants do to make the intervention function  ④Reflexive monitoring:  Participants reflect on or  appraise the intervention.   1. Implementation |
| Kopnina 2010 [11] | Q | Semi-structured interviews and focus groups conducted | Focus group interviews/NA | New Zealand | Asthma | N=19(7/12) | The study examined the causes of patient noncompliance with the prescribed medical regime. | NA | ①Perception of illness and own identity  ②Encounters with medical practitioners  ③Encounter with print and online information on asthma and medical treatments  ④Encounter with patients’ social groups (patient organizations, family, peers, etc.) |
| Lee, 2023[12] | M | A Randomized  Controlled Trial | Virtual focus groups / | America | Asthma | N=9(NA) | To assess the usability, acceptability, and preliminary effectiveness of an electronic SDM application, the  ACTION (Active Conversation in asthma Treatment shared  decision-making) app, that addressed medication, non-  medication, and COVID-19 concerns for asthma. | Online | ①The ACTION app is an insightful communication tool about asthma  ②The ACTION app and efficiency in the office  ③The logistics of the ACTION app in asthma clinic visits |
| Melton et al. 2014 [13] | M | Semi-structured  interviews. Interview data were analyzed using interpretative phenomenological analysis. | Face-to-face interviews/45-60 min | America | Asthma | N=4(0/4) | To use patients’ experiences of managing asthma to better understand the relationship between health literacy and health outcomes. | NA | ①Information desired versus information received  ②Trial and error  ③Expectations of the patient–provider relationship |
| Mowrer et al. 2015 [14] | Q | Focus groups held every 6 months for 3 years.  Qualitative content analysis | Face-to face interviews/15-60 min | America | Asthma | N=200 (NA) | To further explore patient and provider perceptions of asthma and asthma care as part of a larger Asthma  Comparative Effectiveness Study | At the clinic | ①Impact on the care home staff and concerns for the care sector |
| Newcomb  et al. 2010 [15] | Q | Semistructured interviews.  One conversation unit was randomly selected from each subject and coded by two investigators separately. | Face-to-face interviews/20-45 min | America | Asthma | N=104(9/95) | To describe what adult patients with asthma report about their experiences with their own self-management behavior and working with their clinicians to control asthma. | In private examination room | ①Personal constraints  ②Communication Failures  ③Social constraints   1. Medication Issues |
| Norful, 2020[16] | Q | Clinical visits for uncontrolled asthma were audio recorded and inductively analyzed using methods  adapted from grounded theory methodology | Face to face interview/8-28 min | America | Asthma | N=83(17/66) | To explore how Black adults with uncontrolled asthma and their primary care providers communicated about  ICS non-adherence and used shared decision-making to identify strategies to increase ICS use. | At clinic | ① ICS misuse and lack of knowledge  ② External influences yielding personal misconceptions  ③ Patient-provider communication to  individualize plan of care |
| Tapp et al. 2014 [17] | M | The RE-AIM framework and qualitative analysis. A facilitator from outside the group led a discussion using questions from a previously developed participatory evaluative focus group guide. | Focus group interviews/20-30 min | America | Asthma | N=125(NA) | This paper describes the participatory approach used to adapt and implement an evidence-based asthma SDM intervention into primary care practices. | NA | **①Intervention implementation:**  (1) Intervention sustainability  (2) Productivity  (3) Tailoring  (4) Stakeholder identification  (5) Intervention training  **②Participatory process:**  (1) Inclusion  (2) Knowledge exchange  (3) Open communication  (4) Investment  (5) Productivity |
| Tapp et al. 2017 [18] | Q | Description of a case study of patient engagement in outcomes research and examination of the variety of roles patients are engaged in and the associated impact on the study. | Telephone conference/NA | America | Asthma | N=16(NA) | To describe various patient roles  and impact within these large outcomes research study. | NA | ①Lived experience patients  ②Caregiver advocates  ③Research participants  ④Patient advisory board |
| Young et al. 2011 [19] | M | RCT, interviews were conducted with a randomly selected sample of 15 intervention group participants after all 3-month post-intervention follow-up surveys were completed. An interviewer used a standardized guide to conduct confidential, one-on-one telephone interviews. | One-on-one telephone interviews/NA | America | Asthma | N=15(NA) | To assess the feasibility, acceptability, and preliminary impact of a telepharmacy intervention in an underserved, rural asthma patient population. | Telephone | ①Positive and very helpful  ②Improve self-management  ③Questions and immediate feedback  ④Time |

***Note:*** Q= Mualitative syudy; M= Mixed study; PPE= Personal personal equipment.

[1] R. Alzayer, H.A. Almansour, I. Basheti, B. Chaar, N. Al Aloola, B. Saini, Asthma patients in Saudi Arabia – preferences, health beliefs and experiences that shape asthma management, Ethnicity & Health 27(4) (2022) 877-893.

[2] A. Canny, E. Donaghy, V. Murray, L. Campbell, C. Stonham, A. Bush, B. McKinstry, H. Milne, H. Pinnock, L. Daines, Patient views on asthma diagnosis and how a clinical decision support system could help: A qualitative study, Health Expect 26(1) (2023) 307-317.

[3] A.L. Caress, K. Luker, A. Woodcock, K. Beaver, A qualitative exploration of treatment decision-making role preference in adult asthma patients, Health Expect 5(3) (2002) 223-35.

[4] D. Eassey, H.K. Reddel, K. Ryan, L. Smith, The impact of severe asthma on patients' autonomy: A qualitative study, Health Expect 22(3) (2019) 528-536.

[5] M. Gagné, J. Lam Shin Cheung, A. Kouri, J.M. FitzGerald, P.M. O'Byrne, L.P. Boulet, A. Grill, S. Gupta, A patient decision aid for mild asthma: Navigating a new asthma treatment paradigm, Respir Med 201 (2022) 106568.

[6] M. George, S. Keddem, F.K. Barg, S. Green, K. Glanz, Urban adults' perceptions of factors influencing asthma control, J Asthma 52(1) (2015) 98-104.

[7] M. George, S. Abboud, M.V. Pantalon, M.L. Sommers, J. Mao, C. Rand, Changes in clinical conversations when providers are informed of asthma patients' beliefs about medication use and integrative medical therapies, Heart Lung 45(1) (2016) 70-8.

[8] M. George, A. Arcia, A. Chung, D. Coleman, J.M. Bruzzese, African Americans Want a Focus on Shared Decision-Making in Asthma Adherence Interventions, Patient 13(1) (2020) 71-81.

[9] A. Hannane, L. Misane, G. Devouassoux, C. Colin, L. Letrilliart, Asthma patients' perception on their care pathway: a qualitative study, NPJ Prim Care Respir Med 29(1) (2019) 9.

[10] G. Hoskins, B. Williams, P. Abhyankar, P. Donnan, E. Duncan, H. Pinnock, M. van der Pol, P. Rauchhaus, A. Taylor, A. Sheikh, Achieving Good Outcomes for Asthma Living (GOAL): mixed methods feasibility and pilot cluster randomised controlled trial of a practical intervention for eliciting, setting and achieving goals for adults with asthma, Trials 17(1) (2016) 584.

[11] H. Kopnina, J. Haafkens, Necessary alternatives: patients' views of asthma treatment, Patient Prefer Adherence 4 (2010) 207-17.

[12] D.L. Lee, J.W. Hammond, K. Finkel, D.D. Gardner, B. Nelson, A.P. Baptist, An electronic shared decision-making app to improve asthma outcomes: a randomized controlled trial, J Allergy Clin Immunol Pract (2023).

[13] C. Melton, C. Graff, G.N. Holmes, L. Brown, J. Bailey, Health literacy and asthma management among African-American adults: an interpretative phenomenological analysis, J Asthma 51(7) (2014) 703-13.

[14] J.L. Mowrer, H. Tapp, T. Ludden, L. Kuhn, Y. Taylor, C. Courtlandt, T. Alkhazraji, K. Reeves, M. Steuerwald, M. Andrew, M. Dulin, Patients' and providers' perceptions of asthma and asthma care: a qualitative study, J Asthma 52(9) (2015) 949-56.

[15] P.A. Newcomb, K.W. McGrath, J.K. Covington, S.C. Lazarus, S.L. Janson, Barriers to patient-clinician collaboration in asthma management: the patient experience, J Asthma 47(2) (2010) 192-7.

[16] A.A. Norful, A. Bilazarian, A. Chung, M. George, Real-world Drivers Behind Communication, Medication Adherence, and Shared Decision Making In Minority Adults with Asthma, Journal of Primary Care & Community Health (2020) 1-7.

[17] H. Tapp, L. Kuhn, T. Alkhazraji, M. Steuerwald, T. Ludden, S. Wilson, L. Mowrer, S. Mohanan, M.F. Dulin, Adapting community based participatory research (CBPR) methods to the implementation of an asthma shared decision making intervention in ambulatory practices, J Asthma 51(4) (2014) 380-90.

[18] H. Tapp, D. Derkowski, M. Calvert, M. Welch, S. Spencer, Patient perspectives on engagement in shared decision-making for asthma care, Fam Pract 34(3) (2017) 353-357.

[19] H.N. Young, S.N. Havican, S. Griesbach, J.M. Thorpe, B.A. Chewning, C.A. Sorkness, Patient and phaRmacist telephonic encounters (PARTE) in an underserved rural patient population with asthma: results of a pilot study, Telemed J E Health 18(6) (2012) 427-33.
