# Supplementary material for: The experience of shared decision‐making for people with asthma: A systematic review and metasynthesis of qualitative studies
Source: Health Expect. 2024 Apr 13;27(2):e14039. doi: 10.1111/hex.14039 (PMC11015866; doi:10.1111/hex.14039)
Supplement: Supplementary file 4 — Supporting information. [file HEX-27-e14039-s002.docx]

Alzayer R, Almansour HA, Basheti I, Chaar B, Al Aloola N, Saini B. Asthma patients in Saudi Arabia - preferences, health beliefs and experiences that shape asthma management. Ethn Health. 2022 May;27(4):877-893.

| **Finding** | **Sub-findings** | **Illustration from study** | **Evidence** |
| --- | --- | --- | --- |
| **Participants’ experience of asthma** | Participants’ experience of asthma | …When I went to see a doctor, she did not do anything for diagnosis like peak flow, I was  not satisfied about the way of diagnosis, it may be better to change her to another specialist  (Female, 35 years old, university level, partial-controlled asthma, ACT™ score < 19). (Pt#1)  I have fears and anxiety because of the need of going to hospital for nebulizer from time to  time (Male, 23 years old, university level, partial-controlled asthma, ACT™ score < 19). (Pt#  13)  It’s embarrassing to use them in front of others, when I have to use them every 6 hours and if  I am in a gathering with others, people start looking and feel worried and ask me what’s  wrong, it is not a comfortable feeling (Female, 38 years old, university level, well-controlled  asthma, ACT™ score > 20). (Pt# 5)  I hope this will never happen to be by myself in case of emergency, I would go to our neigh-  bours, I don’t have emergency contact numbers… (Female, 48 years old, university level,  partially-controlled asthma, ACT™ score < 19). (Pt# 12) | Unequivocal |
| **Participants’ beliefs and perceptions about health and medicines** | Asthma literacy and information needs | I have 3 types of puffers but I have no idea how they work, the only thing I know they relieve  asthma … they are effective sometimes, and sometimes not… (Male, 31 years old, school  level, poorly-controlled asthma, ACT™ score < 15). (Pt# 18)  I wish that healthcare centres make educational programs regularly like every 2 weeks for  mothers who are uneducated, they need programs in a simple way because they can’t under-  stand everything, or they forget the instruction. Those centres are always crowded of people  so it is a suitable place for such programs. (Female, 39 years old, school level, partially-con-  trolled asthma, ACT™ score < 19). (Pt# 10) | Unequivocal |
|  | Beliefs in alternative medicine systems | …In Saudi, we care about using herbal medicines to improve breathing, I only use things  that my mother or grandmother recommended to me … like fenugreek and chamomile,  I’m concerned to use medications from doctor or pharmacists because they are chemicals  …bone pain and weight gain because of cortisone… I sometimes need to use it 3–5 days  every month and it causes side effects (Female, 38 years old, university level, well-controlled  asthma ACT™ score > 20). (Pt# 5) | Unequivocal |

Canny A, Donaghy E, Murray V, Campbell L, Stonham C, Bush A, McKinstry B, Milne H, Pinnock H, Daines L. Patient views on asthma diagnosis and how a clinical decision support system could help: A qualitative study. Health Expect. 2023 Feb;26(1):307-317.

| **Finding** | **Sub-findings** | **Illustration from study** | **Evidence** |
| --- | --- | --- | --- |
| Diagnosis: The patient experience | Knowledge and understanding of asthma | It was a bit weird 'cause I'd never had it before and [obviously it] was … like, I thought it was quite late. I thought it was one of those things you just had as a kid and then, like, you had it from the beginning and that was that. (P/young person/1, female, 16–30, site 5)  Somehow you associate it (asthma) with really sick people. I don't know. I didn't sort of think of it as a kind of a manageable issue. Sort of, these people who had maybe asbestos poisoning to their lungs or something like that. A very dramatic thing. (P/adult/6, male, 41–50, site 1)  We bought a house which we then discovered had a lot of hidden mould issues and I think that's been a contributor to all of this [….] The asthma diagnosis really helped. (P/adult/6, male, 41–50, site 1)  I was actually diagnosed accidentally, but I was glad I was diagnosed at the time. I was actually meant to go about my toe because I was arguing with my son when he was about five years old and I got my toe jammed under the door and it was bruised. So anyway, I went to the doctor about it and he noticed that I was a bit wheezy so he decided that he would do a test. And he turned round and said, yeah, you're asthmatic. (P/adult/4, female, 41–50, site 2) | Unequivocal |
|  | Communication | Cause all along they're like, oh there's no official test so this might not be, so you'll just need to try this and try that and see if it works or not. So, it's quite a … like, unsure and quite a long process sometimes. (P/young person/1, female, 16–30, site 5)  There was never any concrete diagnosis, so I don't know whether I have a pre‐existing condition now or not. (P/adult/9, male, 41–50, site 4)  ‘It took a while. It was sort of an ongoing thing over … well, I'd had sort of recurring colds and kind of persistent things like that, with kind of wheezing and coughing and so on. And […] my GP, I think was quite methodical about this so there were various tests and eliminations and so on. So, it had come over a couple of years’. Yeah, but I think it's the right way to do it. I was very happy with it. (P/adult/6, male, 41–50, site 1)  You sometimes feel that people are just giving you a decision but not explaining it in enough detail. [….] Even like when my mum's been there with me, it's just been, kind of […] like none of us have fully understood how I have asthma. (P/young person/2, female, 16–30, site 2)  I think the thing about the nurses are … especially the asthma nurses, they, kind of, already […] I mean, they only see you once a year but they know you a bit better so they can, kind of, explain things in a bit more user‐friendly way, I guess. (P/adult/2, female, 21–30, site 1) | Unequivocal |
|  | Receiving and retaining information | I think the thing about the nurses are … especially the asthma nurses, they, kind of, already […] I mean, they only see you once a year but they know you a bit better so they can, kind of, explain things in a bit more user‐friendly way, I guess. (P/adult/2, female, 21–30, site 1)  I probably got a leaflet or something like that, that had two or three pages in it, then, and then, well, a leaflet, you put it down and then it disappears. (P/adult/7, female, 51–60, site 1)  I'm a digital person. I hate bits of paper, 'cause I lose bits of paper. (P/adult/10, male, 61–70, site 4) | Unequivocal |
|  | Self‐management | The only problem, if it could be said to be a problem, was I didn't know how to use the inhalers correctly. I don't believe, I don't remember being told how to use an inhaler. (P/adult/3, male, 61–70, site 1)  I know everybody talks about their asthma plan, but mine is not like … I've not got any asthma plan written down, but I mean […] I know myself and I've got an oximeter in the house as well now that I will test on these various things. (P/adult/13, female, 41–50 site 4) | Unequivocal |
| Patient experience and views | Patient experiences of screen sharing | Well, I don't really think like that is a nice thing to do … Aye, I'm just thinking that (screen) was a bit private, you know, would that not be a bit private to them? (P/adult/12, male, age 51–60, site 6)  Certainly, in the hospital in most sessions. I'm quite curious as an individual anyway, and dangerous because I have a little bit of knowledge, so I've been looking at the numbers they were copying down. I think in the consultant conversation he was definitely pivoting the monitor so we could look at it. I can't remember what was on it, but I do remember that seating arrangement to both look at it. (P/adult/9, male, 41–50, site 4) | Unequivocal |
|  | Online health information use | I think I'm of the generation that what the doctor tells me I believe him. I tend not to look up illnesses myself. (P/adult/11, female, 61–70, site 4)  I don't go online so much […] because I work for a health organisation. And I know that doctors get annoyed with, sort of, patients looking up symptoms online before actually going to see them; and then thinking they've got something when they've not actually got it. So, that's maybe one of the reasons I don't tend to sort of go online to look out for health problems and things like that. (P/adult/1, female, 41–50, site 1)  I looked it up, which I never normally do, asthma symptoms. And it's because I was still coughing and I'm … the thing I says to my brother and sister, I'm not convinced I've got asthma. I think it's a chest infection […] So … what I read on the Internet, sadly […] confirmed what I was feeling [was asthma]. (P/adult/8, male, 61–70, site 3)  Everything seems to be online, and everybody seems to have an opinion and so easily accessible […] information that you need, and you know, you've got your asthma, you know, groups online. (P/adult/13, female, 51–60, site 4) | Unequivocal |
|  | Patient views on an asthma CDSS | And he could say, I don't know, let's say there's various fields on your screen, if five out of these ten fields are ticked, the chances are, that you've got asthma or whatever disease and as you can see you've got seven of them ticked; you know, something like that. A visual representation. (P/adult/10, male, 61–70, site 4)  If there's a simulation or something like that, ‘Here's how it looks when it's really bad’ and ‘Here's how, what’, ‘Here's how an inhaler, what it does to your lungs’, ‘Here's what specific medication does’ and stuff like, yeah, I think that would be very interesting […] just looking at it on a piece of paper, is not the best. I think seeing some kind of simulation would be much more helpful. (P/adult/7, female, 51–60, site 1)  I think it's a good idea. I think it would help quite a lot 'cause the big problem I had was that I wasn't using my inhaler correctly and then I wasn't seeing an improvement on … kind of, on my, like, lung capacity essentially. So, I think if I'd, kind of, had that understanding earlier on then I would have been more dedicated to using my inhaler the way that I'm meant to. (P/adult/2, female, 21–30, site 1)  I mean, I have to say that on these indicators alone, my family history was ‘no none’. At that point I didn't really have any allergies, they have come on since. Also, my coughing had, ironically, stopped by the time … after the first episode my coughing had stopped because of the operation. Also, I didn't have a wheeze. (P/adult/13, female, 51–60, site 4) | Unequivocal |
|  | Barriers and facilitators to a CDSS being used | So, I think this system would be good but if it's just the system and then a very overworked GP that doesn't make eye contact, it's not really going to work. It would be, kind of … you know, you'd have to have the right person who was interacting in … on it with you. (P/adult/2, female, 21–30, site 1)  Between yourself and the health professionals, this might be a little bit of a focal point for the conversation. So, I think that's likely to work well. (P/adult/6, male, 41–50, site 1) | Unequivocal |

Caress AL, Luker K, Woodcock A, Beaver K. A qualitative exploration of treatment decision-making role preference in adult asthma patients. Health Expect.

| **Finding** | **Sub-findings** | **Illustration from study** | **Evidence** |
| --- | --- | --- | --- |
| **Rationales for role preference** | Patient’s level of knowledge | I’ve got the disease or ailment, whatever you call it – I’m not the man with the knowledge – the doctor’s the man with the medical knowledge, not me.  **(PC02)**  Well I think still the doctor because he knows what he’s talking about more than, I mean I’m not  medically trained so I don’t really know what I’m talking about. He’s studied it, he knows more than I do about all the drugs and everything so it should be up to him.  **(PC13)**  Erm well because I think it is important to understand your own asthma and your treatment, what is being offered to you and why. Erm and I like to take responsibility for my own asthma.  Anyway, so I think that is important. I think more people could do that if they had the information or they knew more and it makes you feel more responsible for your life. I don't feel completely.  dependent upon the doctor, which some people do when they suffer from asthma because they have got to go to the doctor all the time. But when I need to see a doctor then I see the doctor and I take his advice as well and I am happy to take his advice or his opinion but I do think it is important to be able to share it (the decision).  **(SC14)** | Unequivocal |
|  | Trust in health professionals and in efficacy  of treatment | I think it’s just trust, trust in the doctor. You know, I don’t think I can say any more than that  really … Even if I’m just a little bit, er, or if I’ve got a bit of a cough he’ll say, oh come on, we’ll have a listen … And he tells me exactly what’s going on … and er, he always seems to get things right for me.’  **(SC09)** | Unequivocal |
|  | Length of time with condition | I certainly think erm you have got to have experience of your own asthma to be able to make  the decision because I wouldn’t have made the decisions I make now 15 years ago. I didn’t know  enough about it, I did not know about my own asthma and it is only experience of that that lets  you know really what to do and why. I don’t think anybody that is newly diagnosed with  asthma could really know when to actually take their own drugs and be able to manage it that  way.  **(SC14)** | Unequivocal |
|  | Severity of condition at decisional juncture | I think in an emergency situation in the majority of cases the decision about what medicine is used is more a medical decision than a patient decision. Any decision about not using a particular medicine has to be taken out of the context of the actual situation where the person is in dire need. **(SC01)** | Unequivocal |
|  | Lifelong nature of asthma | It’s me that has to deal with the medication, with the treatment the doctor doesn’t. He doesn’t have to live with it 24 h a day, I do. He hasn’t got the problem. I’ve got to consider how it’s going to affect my quality of life. **(SC02)** | Unequivocal |
|  | Perception that ‘It is my body’ | Well it gives the patient some sort of decision about how their life is run and that’s what the  person should have … because it is your body. **(SC01)**  It’s your body, your life, what’s going to happen to you is, is up to you. **(PC10)\**  … more or less as if you’re like an animal really. I mean you take an animal to the vet and he does all the work and that’s what happened there(at the hospital). They do it and they never asked me what I wanted they just said ‘‘Oh, you’ve got asthma we’ll cure you in fortnight’’. And that was it. **(PC02)** | Unequivocal |
|  | Characteristics of the individual | But I think it’s going to vary very much from patient to patient, except for reasonably intelligent  people that can manage it themselves, where perhaps like some less fortunate people would prob-  ably have to be told what to do**. (PC04)**  You know if the sufferer, if the asthmatic is not prepared to go out and try and find it, the GPis  not psychic, he can't know what to tell you. So it is a two-way thing, you as in the medical side  need to tell us make us more aware, make others more aware, make us aware of what’s going on,  the new issues and we, if we are concerned about our own health, need to come out and start  asking the questions and getting the answers. **(SC19)**  The way he talks, the way he treats patients, his manner, his listening ability, his supportiveness,  maybe his sympathy sometimes, his tolerance, his acceptance of, er, his acceptance of the importance of the patient, of what a thing might be, as againstthe importance he sees it – which I think is very important. I just think to communicate again. **(PC07)**  We changed doctors to this one’cos the other one, he was always – he never made us feel as though he wanted us there. And you have to feel as though you can discuss something with your doctor, and if he doesn’t want you there he’s like trying to get you out of there so he can see somebody else. And he didn’t seem to put all the effort into it or prescribe you such and such a thing. And his attitude in general the whole family thought ‘‘get lost’’. (PC09). | Unequivocal |
|  | Patient’s response to health professionals | He has got expertise and at the end of the day, you know, he is the one that is going to help me, yes, so I just feel that, because I do have faith in the doctor, and I know that he knows what he is talking about.  **(SC04)**  I am quite happy if the doctor says X, then that should be what happens. All the treatments I have  ever had, which isn't particularly many are formed that way. (SC08)  I mean, if your car breaks down you don't go to a plumber with it do you?  **(PC02)**  … (it’s) a form of idol worship, I suppose really,which I just don’t believe in, but a lot of people do because a lot of people think if you’ve got a degree you’re something special to begin, where I don’t. **(PC07)** | Unequivocal |
| **Facilitators to and bars from participating**  **in treatment decisions** | Perceived facilitators to patient participation in treatment decision-making | Respondents highlighted a number of factors which could facilitate their participation in treatment decisions:Possession of sufficient information.Health professionals being willing to listen.Good provider–patient relationship.Continuity of care.Assertiveness on the part of the patient.Length of time with asthma. | unsupported |
|  | Perceived hindrances to patient participation in  treatment decision-making | Lack of knowledge regarding the condition and its treatment.Health professionals with poor inter-personal skills. Lack of time.Personal characteristics of the patient.Health professionals’ unwillingness to listen or accept patients’ expertise. Being in a life-threatening situation. | Unsupported |
| **Other considerations** | Specialism vs. generalism | You know I wouldn’t expect him (GP) to be a specialist in just the things I suffer because the next patient is going to have something completely, completely different. So he can’t be a master of all trades in that respect. But so long as he appreciates that, not just asthma but anything,  he could put you through somebody else in his practice or there is someone else that he can send the patient that he understands more, then he should have the decency to say that. **(PC03)**  Erm my GP has got a limited knowledge whereas somebody like Dr X has specialised in it. He is the most important one definitely. If I had to cut out one it would be the GP ... For something as serious as asthma yes definitely. If it was eczema, it’s a different situation, it’s not life threatening, but asthma is and it can affect me so badly you know that sometimes I cannot even get out of bed, I can’t brush my teeth, I can’t breathe, that badly. So yes definitely it is important that I have got that specialist knowledge.  **(SC19)** | Unsupported |
|  | The role of health professionals other  than doctors | I think they(nurses) should(be involved in treatment decisions), to be honest with you the answer is yes. But I don’t think it’s acceptable at the present stage, because I don’t think the public have enough respect for them which they should have ... But I think the final word has got to come from the doctor or from the consultant. And the nurse is very good for saying ‘‘Dr So would do this or Dr X advises that’’. They(patient) would listen to that quite healthily, but I don’t think they(nurses) should tell you what to do. Although they might know better more so than the doctor ‘cos they’re doing more of it. I mean the perfect example, is nurses with injections in the arm or the hand. Well the nurse  s can do it just like that, and consultant maybe one a month and he’s faffing and faffing. But some people want the big man to do it, but the big man can’t do it whereas the little girl can do it. But I think when it comes to that kind of decision(i.e. re treatment), it’s got to come from a qualified (person). **(PC07)** | Unsupported |

Eassey D, Reddel HK, Ryan K, Smith L. The impact of severe asthma on patients' autonomy: A qualitative study. Health Expect. 2019 Jun;22 (3):528-536.

| **Finding** | **Sub-findings** | **Illustration from study** | **Evidence** |
| --- | --- | --- | --- |
| **The desire to live an “unconstrained” life** | Health‐care interactions | Jane reported: Never give up…If you think there's something wrong, that someone's not saying, speak up. You've seen something [health‐related issue that her healthcare providers didn't pick up] obviously but they didn't take the time to see … speak up. **(Jane, 51‐year‐old woman).**  …the same doctor asked me the same questions on three of my admissions, ‘what do you need?’ And it was like, I need this, this and this… It makes me powerful. I think when you go to hospital you basically give away all your power a lot of the time. Not because you want to but because you’re too sick to bother. So, when you get given it back it’s like you’ve got your power back now, … you’re in charge. …one’s empowering and the other one’s totally disempowering. It sort of means, I’m relying on the fact that you [doctor] know what you’re doing and if you don’t, then I don’t have a say anyway and sometimes I feel like, well, I think we need to discuss what the side effects are and why I’m taking it.  **(Sarah, 59‐year‐old woman)**  Being unable to breathe was described as fear‐provoking, challenging their sense of personal control: “…it's more fear than anything else. Like oh this is bad… yeah that's not a good feeling at all. Especially when you know that like the medication I've taken and that wasn't working.” **(Ben, 60‐year‐old man).**  Chris reported that he would keep a six‐month supply of his medication as this helped him feel like he was in control of his body; he saw not having plenty of medications as a potential threat to his sense of personal control:“I’m out of control, I start to worry. But if you’ve got everything there you need to control it, you don’t worry…nothing worse than not being in control of your body, not being able to breathe… ” **(Chris, 66‐year‐old man)**  Access and adherence to prescribed medications were perceived as enablers to gaining a sense of control over one's life. For example, Jane had a system which provided reassurance that she could carry out her daily activities:“I call it lock and load [a term originally used in relation to firearms, later popularised by a John Wayne movie set in World War II] and I normally have one puffer in here [in one pocket] and one puffer in there [in the other pocket]. ” **(Jane, 51‐year‐old woman)**  Exercising autonomy, however, in some cases could also symbolize the ultimate loss of personal control and choice.  Phil reported that it took him time to come to terms with the diagnosis. He did not understand the severity of his condition, and despite his HCP's advice to not push himself physically on the farm as this may trigger an asthma attack, he exercised autonomy, and as a result of this, he was hospitalized:“Just couldn't‐couldn't believe it [being diagnosed with severe asthma], I was just super human. I just didn't think, that could ever happen to me…We have a fair few grapes, they're virtually picked at night‐time…[and] it started to get really, really bad. So, I… barely got home and went into the hospital, and, they put me in intensive care. ” **(Phil, 74‐year‐old, man)**  Whilst avoiding triggers and adhering to medications helped to provide a sense of control over severe asthma for some participants, for others different aspects of their lives were more highly valued. Asserting autonomy to preserve these, despite the potential health problems that might arise as a result, was evident. For example, Grace reported that her specialist told her not to keep her pet budgerigar (native Australian bird). However, for her this was not an option, as the budgie was part of her family:“I was told that I was to not have budgies because they carry bird fancier’s disease. Yeah but his cage gets kept clean and he runs the house. So, what my specialist doesn’t know won’t hurt me.”**(Grace, 41‐year‐old woman)**  My first specialist said ‘oh your condition is bad’, and his reaction was to stick me on cortisone medication straight away and…I went back to my GP and said look I wasn’t really comfortable with him and I asked to be referred to another one.  **(Dylan, 65‐year‐old man)**  I’m training him [general practitioner]. Um, certainly not an equal partnership no but…‐‐ I‐I tell people I'm trying to train him and to be quite honest I think I am. I think there is a lot of things he doesn’t understand…they don't like anyone that is an expert over them…we are the ones that live with it.  **(Mel, 66‐year‐old woman)** | Unequivocal |
|  | Employment | I never took as much sick leave as I should have unfortunately. I left the bank with over a year’s worth of sick leave accumulated. Being manager of the department, I felt I had to be there as often as possible and I know I should not have been there certain days. **(Dylan, 65‐year‐old man)**  I am a very hardworking person. Normally even when I am sick, next morning I’ll try to do my job, because I’m a very responsible person…. I feel if I stay at home and do nothing, I feel worse, even my body becomes worse.**(Joseph, 70‐year‐old man)**  Jane reported that work (physiotherapist) was a big part of her life to the point that despite regular hospitalizations she had her uniform in the car and would still go to work. It was getting incredibly incredibly difficult… [and I had] to reduce the types of patients [I] was seeing, she had to turn down patients who came in sick, so work was much more of a risk. Eventually, she had to stop working and life became very reduced. **(Jane, 51‐year‐old woman )**  Hugh (chef) reported that his HCP told him to give up work, but he felt that this advice was kind of belittling…it was kind of insulting when he said, you know, you can't do this, you need to be more realistic. Despite acknowledging that he lives with an unpredictable condition and recognizing his specialist's recommendations, he was not prepared to give up his profession and responded with: I'll take my chance. Acting autonomously, even when that involved resisting controlling efforts of expert others, and the possibility of poor health, enabled this young person to have a sense of purpose.**(Hugh, 19‐year‐old man)** | Unequivocal |
| **Preservation of self‐identity** | Maintaining valued roles | You’ve got kids that want to play with you, and you can’t. And the worst thing has been the amount of times I have been in hospital when it’s my kids’ birthdays or I’ve just been too sick… we try and make up for it, you know, I buy way too much stuff to try and make up for it…our big purchase was we bought them a trampoline, and that cost a fortune. Thank you Credit card.**(Donna, 39‐year‐old‐woman)**  Another participant, Phil, a third‐generation farmer who owned eight farms, valued the ability to preserve a multigenerational family legacy whilst living with severe asthma. He compared his HCPs as experts in their field to him being the expert in farming …they're the professionals, they're the equivalent of Phil of farming, I know anything in farming…. He spoke at length about how he would always preserve his identity of being a farmer and would not let his condition get in the way: I started when I was 15, so. Uh, just farmed all me life… I'm doing what I love to do, and, uh, the alternative to that, take it away from me, and I definitely will die. So, there's no way I'm going to give up what I'm doing.  **(Phil, 74‐year‐old, man).** | Unequivocal |
|  | Searching for normality | Jane described that, prior to being diagnosed with severe asthma, she defined having a “normal” life as being an active person, enjoying the outdoors and being independent: I was living like normal…. I used to dance. I used to pick fruit. I used to mow lawns. I used to be able to take care of myself. ince being diagnosed with severe asthma, she described struggling to define her identity. However, one way of exercising her autonomy was by being realistic and, rather than surrendering to the illness, preserving normality by doing things at a slower pace:“I’ll be realistic …being able to travel, to be able to go fishing, being able to cook around the campfire, being able to look after myself, still being able to help people that’s been a big thing for me and I've lost that…I've got to do something whether it be you know I might try to cook dinner and fail at it but if I don’t try, what am I going to do? Sit here and mourn away.” **(Jane, 51‐year‐old woman )**  A normal life would be being…able to work in a normal job for the rest of your life, being able to afford to pay your mortgage, being able to afford to do all those things that money comes with. I’ve still got as normal a life as I can. I've got friends, I've travelled, I've got my home, which I'm still paying… I had to beat it to still be here. I've still got it [severe asthma], but it's like we've reached an agreement.  **(Richard, 54‐year‐old, man)**  I just want to be left alone. I just want to sit in the corner of the couch and, and I’ll sit there for a week…I helped the guy next door…he didn’t know how to build a little retaining wall…I was just sopping sweat and he goes, ‘what’s wrong with you?” And like I was just dripping wet and couldn’t breathe and just using the, the puffers. And it wasn’t a, a big job to do. But… so you’re just embarrassed…  **(Archie, 58‐year‐old, man)** | Unequivocal |

Gagné M, Lam Shin Cheung J, Kouri A, FitzGerald JM, O'Byrne PM, Boulet LP, Grill A, Gupta S. A patient decision aid for mild asthma: Navigating a new asthma treatment paradigm. Respir Med. 2022 Sep; 201:106568.

| **Finding** | **Sub-findings** | **Illustration from study** | **Evidence** |
| --- | --- | --- | --- |
| **Content Preferences** | Importance of accessible language | 1. **Use of lay terms and simple language**   “Some of the wording might be very hard to comprehend. […] So maybe simpler words, for people who are not that bright to speak.” **(Patient 4, FG1)**  “[…] people come from very wide range of life backgrounds, ages, even education levels, and so on. […] I think a lot of people would not be able to get through the text, would not be able to understand it.]” **(Patient 15, FG4)**   1. **Availability in multiple languages**   “Canada is a very diverse country, people speak different languages and so we have to take into consideration who is looking at this material and they're getting it before they see their doctor.”  **(Patient 8, FG2)** | Unequivocal |
|  | Priorities for comparisons between therapeutic options | 1. **Convenience of regimen**   “A is good ‘cause you wanna know how often you're gonna take this.” **(Patient 13, FG3)**   1. **Cumulative drug exposure**   “I liked the piece where it tells me that the amount of medication that's actually coming into my lungs on the three different levels of treatment.” **(Patient 11, FG3)**   1. **Biological effects (when linked to a patient-relevant impact)**   “When the decision aid asks me to rank my priorities, the swelling in my airways is not something I care about, because I can't map that swelling in my airways to something that affects me in a day-to-day basis. The question seems very irrelevant. But when I look at this [outcome comparison] page, I see that the different medications will change the swelling in my airway, which therefore changes my day-to-day activities, and then I find this page informative.”  **(Patient 2, FG1)**   1. **Effects on symptom control and prevention**   “[…] something that's important is addressing that you might be able to live a normal lifestyle. Even if you think you're okay, if it's affecting you a couple times a day in a week then maybe it's not something you need to live with. […]” **(Patient 2, FG1)**   1. **Effects on need for urgent healthcare or death**   “[…] to know that option two in fact does even better than a daily steroid in number E − how many people will have a severe asthma flare up over a year, that's a good sellin’ point.” **(Patient 20, FG5)**  “We should know [how many people will die from asthma] because a lot of people think that no one really dies from asthma.” **(Patient 4, FG1)**   1. **Medication costs**   “[…] how much does it cost is a valid criteria for anybody's decision-making.”  **(Patient 16, FG4)**   1. **Side-effect profile**   “I think that you should know the side-effect of each medication so you can make a concise decision on which one is your preference. We need to know […] because the side-effect might be greater than the disease itself.”  **(Patient 4, FG1)** | Unequivocal |
|  | Directly addressing commonly held pre-existing beliefs about each therapy | “[…] there's a section on myths with corticosteroids. That's useful. I grew up hearing people asking me, ‘aren't you worried about taking steroids?’ without any reason or rationalization.”  **(Patient 2, FG1)** | Unequivocal |
| **Format Preferences** | Minimizing text content | “I think your message gets a little bit lost in all the other information that's given like it's maybe too detailed […]” **(Patient 1, FG1)**  “[The decision aid] is a very text-based. […] I'm not positive on that.”  **(Patient 15, FG4)** | Unequivocal |
|  | Importance of images | To reduce overall cognitive load  “I think the one visual thing that I always found helpful, was the diagram of showing what is asthma, what it is doing to your lungs when you have an asthma attack, that constriction and that the medication helps to expand it.” **(Patient 15, FG4)**  To convey complex numerical comparisons  “I think that it might be quite helpful for people to see the amount of medication actually coming into the lungs, represented in the forms of inhalers, and then from there they can gauge whether they want to have a lot in at once or just a little bit as they need it.”  **(Patient 11, FG3)** | Unequivocal |
|  | Importance of minimizing overall tool length | “I think the decision aid is way too long. […] It's a couple things: the number of pages, some of the words are redundant” **(Patient 4, FG1)**  “The one pager is a nice quick synopsis, it's a little bit easier to navigate and understand and follow.”  **(Patient 14, FG4)** | Unequivoca |
| **Preferences for Process** | Features required to enable completing of the decision aid prior to the clinician visit | 1. Pre-visit prompt for completion   “An email reminder will be good: ‘When you're coming to see the doctor please bring this decision aid, remind people to fill it out, maybe a week before or something like that because usually they get a reminder anyway of the appointment, right?”  **(Patient 13, FG1)**  “I would do it for sure if I was asked to, a hundred percent. I personally would likely do it […] if it was just emailed to me.”  **(Patient 14, FG4)**   1. Need for clear eligibility criteria before entering the decision aid   “I think that the page “Who Is This Decision Aid For?’ needs to be first, before you enter the decision aid.”  **(Patient 11, FG3)**  “Instead, if it said the decision aid is for you if one, two, three, or the decision aid is not for you if one, two, three …”  **(Patient 6, FG2)** | Unequivocal |
|  | Need for clinician interaction to reach final therapeutic decision (e.g. use of conversation aid for final decision with clinician) | “I'm not sure whether it's our decision to pick [option] two or three. Asthma is a medical condition that needs a medical professional and a medical professional to make a decision, so as a patient, I would go to my doctor and say what do you think I should be doing. I'm not sure how much of a decision-making tool it can be for a patient.”  **(Patient 6, FG2)**  “I think [the conversation aid] is likely to be much more useful than this [decision aid]. […] I think your greatest opportunity for providing this education would be at the appointment itself or when the patients are in the clinic.”  **(Physician 4)** | Unequivocal |
|  | Little scope of action | **[NA6]:** So we just had a certain wheel. Leave for vacation days or days off that had already been planned in the past were not given during this time. But there needed to be as little contact as possible.  **(P2499)** | Unequivocal |
|  | Decision making | **[N1]:** If you send someone to the hospital, just because the resident falls, they have to be in quarantine for 14 days. And then you think about it five times. And, therefore, you need experience. You have to stand up and say, (…), I have now done this and that. There is no danger right now in delaying, and the resident should stay here for now. And then you have to be able to deal with this decision, because it means that the resident stays at the institution.  **(P2500)** | Unequivocal |

George M, Keddem S, Barg FK, Green S, Glanz K. Urban adults' perceptions of factors influencing asthma control. J Asthma. 2015 Feb;52(1):98-104.

| **Finding** | **Sub-findings** | **Illustration from study** | **Evidence** |
| --- | --- | --- | --- |
| **Monitoring and responding to deteriorating control** | Monitoring and responding to deteriorating control | **[Participants with controlled asthma]:** ‘What I was doing that was wrong. I took my albuterol, and I kept usingthe albuterol hoping that it was gonna’get better. And really Iwas doingmore damage to myself by keep using the albuterol.”  **[Participants with uncontrolled asthma]:** “I go to the doctors on a regular basis to have myself checked out. So maybe I didn't do all those things before. I wasn't so proactive before about it, because - like after the last hospitalization it was like okay, I think you got a wake-up call. Like this is real, it ain't playing.’’  **[Participants with uncontrolled asthma]:** “Sometimes I wasn't taking the inhaler (CS), because like I'm fine now, you see what I'm saying? So yeah, I'll be honest: I don't take it when I'm fine. I don't take it every daylike I'm supposed to. So I'm admitting it. And of course they get on me about it. But I be truthful. Like now, I don’t have acold. I'm fine. So I didn't take it.” | Unequivocal |
| **Beliefs about ICS and SABAs** | Beliefs about ICS and SABAs | **[Participants with controlled asthma]:** It's the one thing I try to do, I take my (brand name ICS), because that's maintenance, and it keeps me really open.’ However, one subject did describe her ICS as a band aid’ that caused weight gain and “didn't solve the problem.  **[Participants with uncontrolled asthma]:** Many participants were skeptical that asthma could be controlled and offered alternatives to ICS such as drinking water and getting adequate rest. Others commented that ICS could be helpful in achieving asthma control but that forgetting doses was common. | Credible |
| **Triggers avoidance/remediation** | Triggers avoidance/remediation | **[Participants with controlled asthma]:** I could get rid of this cat that's here for the mice, because I'm allergic to cat and dog dander, but I need a cat because we have mice. They're in the corner house, and you know there's all these little holes somewhere and they do get in here, I ain't even gonna' lie. And this cat has to be here and we just got this cat. I came home from the hospital there was a cat here. So if we didn't have to have a cat that would be a lot better as well. He's starting to grow on me.  **[Participants with uncontrolled asthma]:** My dog went away for the summer; she went to my daughter's house, because I went to Florida. Then I missed her...but then when she came back it affected it (allergies)then, because I guess I wasn't used to her hair anymore andthen I was constantly sneezing, constantly sneezing and coughing and gagging for air. So I had to take and bathe her, because see I'll wash her every three days with baby wipes. But I love my dog, she's my baby. I have a cat too...the reason why I have the cat is because my youngest granddaughter has asthma - the one that lives in the house with me has asthma - and they have to be - I don't know what it is with them, when there's not an animal in the house their asthma is worse. It triggers. I guess because the dog, they're always - the dog is always licking them in the face. I don't know. But long as the dog is there she's okay...when he came back she was having like sneezing sneezing, sneezing for a little while, but then it went away. | Unequivocal |
| **Role of primary care** | Role of primary care | **[Participants with controlled asthma]:** One of the things is to be upfront with your doctor. And if you feel that your doctor is not giving you the right treatment or he's not listening to you, change doctors.”  **[Participants with uncontrolled asthma]:** “See the doctor...when-ever they want to see you’’ and another said Do what you got to do, do what you supposed to do. If you diagnosed with asthma, they (providers) know what they talking about. Don't think they don't know.”  **[Participants with uncontrolled asthma]:** The first person who's gonna’ prescribe you something is your regular (primary care provider). He's the first person you're gonna' have to see before you can get to the pulmonologist. Okay? So he needs to be better trained on how to prescribe the proper medicine. In other words, don'tgo first to steroids, okay, because sometimes that's not what we need.  **[Participants with uncontrolled asthma]:** And I did go see a nurse practitioner and she was like Yeah, you're just crackling everywhere. Here's some prednisone.’I was like, no, that's okay. Let's start with antibiotics, and then we'll see. And I did take antibiotics and it did clear itself up within two days.  **[Participants with uncontrolled asthma]:** If you don't feel any better then I guess you have to go seea doctor. But usually my treatments work and I don't have to go see my doctor, because all they gonna' do is give you some more prednisone, which in turn is not really good for you. | Unequivocal |

George M, Abboud S, Pantalon MV, Sommers ML, Mao J, Rand C. Changes in clinical conversations when providers are informed of asthma patients' beliefs about medication use and integrative medical therapies. Heart Lung. 2016 Jan-Feb;45 (1):70-8.

| **Finding** | **Sub-findings** | **Illustration from study** | **Evidence** |
| --- | --- | --- | --- |
| **We felt**  **Themes identified when the provider had knowledge of**  **patient’s beliefs** | Negative ICS beliefs | **When providers were informed that the patients had negative ICS beliefs, they focused**  **the conversations around ICS.**  Provider: But you. didn’t take steroids, because?  Patient: Because it’s evil and I didn’t need it, clearly. I clearly didn’t need it.  Provider: It’s not evil. It’s not evil.  In another visit, the provider learned that the patient had  decreased twice daily ICS doses to once daily, fearing tolerance:  Patient: Well here’s the thing, I won’t want to be like .immune to it.  Provider: To what?  Patient: To the [brand name ICS]. So I take it once a day. So I think when I start getting worse, I’ll start with twice a day. You see what I’m saying? Provider: ‘Cause you don’t want to build up a tolerance to the [brand name ICS]?  Patient: Right. I don’t want to [.] I don’t take it at night. A lot of times at night I find myself wheezing.  Provider: Okay. Who told you that you would get a tolerance to it?  Patient: Me.  Provider: Okay.  Patient: No one. Is that true?  Provider: No. So you should take it twice a day.  **Other patients associated symptoms such as “fingers or stomach cramps” to side effects of corticosteroids and providers tried to correct some of the misconceptions of negative beliefs. Conversations regarding negative steroid beliefs were very brief**:  Provider: Did they give you steroids?  Patient: Yeah, they gave me the Prednisone.  Provider: Yeah.  Patient: And that stuff, I think I’m having side effects from that.  Provider: From back then?  Patient: Yeah, ‘cause my body cramps and like I still .my body is still . my muscles still will cramp up.  Provider: Hum.  Patient: Like I might go to bed and turn and my stomach muscles are just. they’ll just grab. Sometimes it’s in my feet. Sometimes my fingers cramp up and I never had it until I took those.  Provider: Prednisone doesn’t usually do things like that, but I’m wondering if there are other reasons . | Unequivocal |
|  | IM use for asthma | Provider: So do you do some of these things sometimes, when your asthma’s acting up?  Patient: Yeah, I turn the fan on. I sleep with the fan on. If there’s a breeze blowing through the window, I don’t need the fan. Then I’m okay, but it does help. And my mom used to always  tell me that coffee helped [control asthma symptoms].  Provider: Yeah, there’s something in coffee that works similar to, have you ever heard of theophylline? It’s a really old asthma medicine.  Another patient also described the use of the fan to help with asthma symptom relief:  Provider: So do you use fans in your house?  Patient: I have a ceiling fan.  Provider: Does that make your breathing worse?  Patient: Oh, I love my ceiling fan.  Provider: Okay.  Patient: And I have a standup fan.  Provider: You use the ceiling fan at night?  Patient: Uh-huh, all the time. | Unequivocal |
|  | Decision-making | **Providers discussed patient self-management decision-making to gain a better understanding of the thought processes involved in selecting prescription medicine or culturally-relevant IM for asthma. An exemplar follows**.  Provider: How often are you getting asthma attacks?  Patient: Attacks? Not necessarily.  Provider: So you are preventing a full [attack]?  Patient: I would lay there and think, yeah, maybe it will pass. And then I took a bath and maybe the steam and everything would work. And I had to use my nebulizer.  This example illustrates the thought processes that the participants engage in when faced with acute asthma symptoms and how they prioritize their treatment decisions at the time of the  acute symptoms. For this participant, laying down and waiting for the symptoms to pass was the first choice, followed by taking a bath in hope that the steam will help. When the participant  realized that the symptoms were not improving, she decided to use the nebulizer. In another visit, the provider discussed and reinforced the management of asthma medications to better control symptoms. The provider discussed with the patient how to decide on which medication to use and their benefits(as needed versus continuous use):  Provider: Right. Okay. So if taking a deep breath bothers you, or you cough more, or you feel short of breath, right.  Patient:(Interrupting) Right.  Provider: So you got some medicines to take that could help you.  Patient:(Interrupting) Right.  Provider: Only one of them is as needed.  Patient: I don’t know, which one, that purple one?  Provider: Not the purple one. That’s not as needed.  Patient: Oh.  Provider: The other, albuterol.  Patient: Albuterol  Provider: Puffer, that’s the as needed one.  Patient: Okay.  Provider: But you don’t use that too much?  Patient: No.  Provider: No? Okay, well that’s good. But how about the [brand name ICS]?  Patient: I use it when I’m. I can’t sleep ‘cause of coughing, it’s like something’s tickling my throat and I can’t get it out.  Provider: So that’s not how it was prescribed, right?  Patient: No.  Provider: No [Laughing]. Okay, one puff two times a day, remember?  Patient: Oh, two times a day, okay.  Provider: Yeah, remember, one puff two times a day?  Patient: I remember now, one puff two times a day.  Provider: Okay. That medicine doesn’t work as needed. It’s got to be in your system for a couple days before it even works for you. | Unequivocal |
|  | Healthy lifestyles | **Healthy lifestyles Providers also discussed conventional non-pharmacologic approaches to improved asthma control, including diet, weight loss, exercise, and smoking cessation. Discussions were tailored to patients’ needs, situation, and their ability to modify certain behaviors like smoking**:  Provider: So you always tell me you feel short of breath, right?  Patient: Right.  Provider: Okay. You’re also still smoking, right?  Patient: Yeah.  Provider: How much? How many cigs?  Patient: Five cigarettes.  Provider: Five? Oh, that’s good.  Patient: Five cigarettes until I get stressed out.  Provider: And well that’s every day.  Patient: Well I don’t smoke. I’m not a. I’m like a casual smoker, cause I can go all day without smoking a cigarette.  Provider: Good. So how about, you know. the cigarettes are an irritant, the smoke, even if you don’t believe anything about nicotine, okay?  Patient: Right.  Provider: The inhaled smoke and the burning of the paper and the burning of the leaves, no matter what kind of leaves they are.  Patient: Okay. Okay [Laughing].  Provider: Alright?  Patient: Okay.  Provider: It goes down into your lungs and you’re the one who’s telling me you’re short of breath, unless you like feeling that way. | Unequivocal |
| **Themes identified when the provider did not have knowledge**  **of patient’s beliefs** | Asthma self-management | Analysis of these transcripts found that patient-provider communication focused primarily on the ability to manage the disease through a review of prescribed asthma medications and  emergency procedures, such as when to call the office, as well as inquiries about recent symptoms and need for acute care.  **[Example 1]**:  Patient: Well I don’t know what I’m allergic to, but. my pulmonary doctor told me that sometimes if I get postnasal drip.  Provider: Yeah.  Patient: It affects my asthma.  Provider: Right. So what do you do about the postnasal drip when you get it?  Patient:I’m using my [brand name prescription intranasa steroid].  Provider: Okay. Are you using it every day?  Patient: Sometimes. It dries me up.  **[Example 2]:**  Provider: And then how is your breathing, how’s the asthma doing right now?  Patient:It’s doing okay right now. But, like last month, I had a few problems. Like at night time, bedtime, with breathing. But I got on the nebulizer, you know, and I started, you know, taking  my [brand name ICS] twice a day instead of once a day and then it seemed like it kind of, like, disappeared. I just had, like, that cough. I had a problem with, like, the cough thing. So I just got  some cough medicine over-the-counter.  **[Example 3]:**  Provider: Okay. Alright. Now tell me about your asthma.  Patient: This has been acting up.  Provider:(Interrupting) Since last week.  Patient: Yeah, last week. It’s been acting up a little bit, like um…well it. you know what, it’s reoccurring.  Provider: (Interrupting) So I’m just going to go back to last week, so last week you had just finished the steroids that were prescribed before.  Patient: (Interrupting) Yes.  Provider: And you had just finished the antibiotic (inaudible).  Patient: Right. Oh, that cleared up the cold real good.  Provider: Okay. So the steroids and the antibiotic, you felt better after that, right?  Patient: Felt normal after that, yeah.  Provider: And you were still coughing a little bit?  Patient: Yeah, I’m still coughing. I don’t know why.  Provider: Okay.  Patient: I even took the cough syrup you gave me every night.  Provider: Okay.  Patient: And I’m coughing hard-like and then it’s like a dry cough.  Provider: Okay. So you’re coughing at night.  Patient: Yeah. Yeah, I’m still coughing. I don’t know why.  Provider: But what’s coming up?  Patient: Nothing.  Provider: Nothing? It’s clear?  Patient: It’s clear because of the antibiotics.  Provider: So clear, okay.  Patient: It’s just clear, but it’s hard and it lasts like a long time.  Provider: So it’s a hard cough, okay. How many times are you using your rescue inhaler? I’m going to look at your sheet (inaudible).  Patient: My rescue inhaler I used like maybe twice. Sometimes. now I sleep with it.  Provider: Twice in?  Patient: Twice a day.  Provider: Twice a day, okay.  Patient: But I do my treatments, you know, one at night and one at bedtime, you know, one in the morning and one at night. | Unequivocal |
|  | Healthy lifestyles | **Another common theme was healthy lifestyles. Providers addressed these topics as part of chronic disease management that included asthma, diabetes, hypertension, cardiac problems, back**  **pain, and other conditions.**  Provider: I had really wanted you to focus on losing some weight to kind of help with everything. Really, help with the back pain, help with the breathing. And good job! You did that. Because  last time I saw you, you were at 279. And today, when she weighed you . you were 267. So great job! That’s 13 pound [sic]. That’s great. That’s great. Because that’s going to help, that’s going to help with the back pain, that’s going to help with the breathing, you know? So that’s a great job. And, what have you been doing with that, you just been cutting back? A little bit? Not trying to eat as much?  Patient:I’ve kind of lowered my sugar intake with beverages because I had a thing with the beverages. You know like cut down off of that, even cut down on the sugar that I put in my  coffee and stuff like that.  **In another visit, the provider discussed diabetes control with the patient:**  Patient: My sugar went to 300, I had spaghetti this morning and coffee.  Provider: How much spaghetti?  Patient: Serving.  Provider: Yeah, did you take insulin?  Patient: Four.  Provider: Four?  Patient: Four .  Provider: Before, good.  Patient: Yeah and a cup of coffee.  Provider: What was in your coffee?  Patient: Equal. substitute Equal.  Provider: Good, perfect. So you did 90 units before eating?  Patient: Yeah, right when I woke up this morning it was 262, what did I have last night, I think I might have eat some. I eat a cup of fruit and it wasn’t (inaudible cannot hear patient over typing). But I brought my thing in.  Provider: Oh you did, oh good, I want to see it okay. Nice, nice, nice. You had a 103, you had a 167, those are good, 192, your sugars are better than they used to be, by a lot, by a lot. Look at  the dinner ones before 170, 141, 130, 160; that’s perfect. That’s great, that’s really, really great, so whatever you were doing. oh and that was the week of Thanksgiving, so the week of Thanksgiving your sugars were perfect. | Unequivocal |

George M, Arcia A, Chung A, Coleman D, Bruzzese JM. African Americans Want a Focus on Shared Decision-Making in Asthma Adherence Interventions. Patient. 2020 Feb;13 (1):71-81.

| **Finding** | **Sub-findings** | **Illustration from study** | **Evidence** |
| --- | --- | --- | --- |
| **To be Heard and Respected** | To be Heard and Respected | **[Family/Friend Focus group #2]:** “Not understanding like how you feel, like as far as breathing, they [patient] said they can’t catch they breath or something like that and they’re [PCP] not  understanding it and saying ‘It could be your weight’or it could be this or it could be that. You’re not my body, you know?”  **[Mixed Focus group #3]:** “I told her [PCP] ‘I think it’s time for you to call 9-1-1so I can go to the hospital.’ ‘Oh no, you’re OK. Here, I’m going to write you this prescription and you just take these prednisone pills.’ They do what they want to do.”  **[Mixed Focus group #6]:** “Don’t play with my intelligence. Let me know what with him[patient]. Because I have a daughter, likewise who suffers from the asthma. Was hospitalized when she was younger. She’s coming out of it. Just tell me. I’m intelligent enough to know so don’t play with me and say, ‘Well, it’s not that bad’. No, tell me what it is so then I can know what to do and continue to work with you and all.”  **[Patient Focus group #4]:** “I’m going in telling them what I need. They try to tell me what I need. I was like ‘No’. I stop them right [there], ‘I’m telling you what I need. When I go to ER, I need my treatment, I need my prednisone.’”  **[Family/Friend Focus group #2]:** “I was telling them, ‘He’s not ready to go home yet.’  They still discharged home. Probably was home for like 45 minutes. I had to call the ambulance again.”  **[Mixed Focus group #6]:** “I want to be a part of every decision that’s made with  my body and my medication.” | Unequivocal |
| **Wish to Receive Patient‑Centered Care** | Wish to Receive Patient‑Centered Care | **[Mixed Focus group #3]:** “You’ve got actual doctors that do care, but where they at? See, you be searching and searching. You go to this doctor, you think this doctor is all right, and then you  find out this doctor ain’t all right. Then you got to go find another doctor. You just be on a search, just running around trying to find the right doctor”  **[Patient Focus group #1]:** “He came in, he said this, he said that, and he left.”  **[Patient Focus group #1]:** “All doctors aren’t good doctors. All doctors don’t need to be doctors. But the good doctor will go in and say, okay, I know this preventative should be working. Let’s go to the next step and see why it’s not. Then they’ll go into, are you living with pets? Do you have a dog? Are you around people that smoke? And that’s your personal stuff that makes you who you are, the way you live, and some doctors—I know my doctor, let me tell you. She is awesome.”  **[Family/Friends Focus group #5]:** “You come in and say ‘Hello, may I help you?’ I’m not  ordering food! I’m trying to get checked out.”  **[Mixed Focus group #6]:** “I just think medical school should offer a hospitality course. I’m just saying.”  **[Family/Friends Focus group #5]:** “You cannot diagnose me from the chair.” | Unequivocal |
| **Underscore the Risk of Inhaled Corticosteroid Non‑Adherence** | Underscore the Risk of Inhaled Corticosteroid Non‑Adherence | **[Family/Friends Focus group #2]:** “The only way I would get to that point [adherent to ICS] … it’ll have to, have to be real crucial … if they tell me life or death. That I had to take this [ICS]  or I’m going to die … I’m taking an aspirin now, because that was a life thing.”  **[Mixed Focus group #6]:** “She [PCP] said ‘You want to live to see tomorrow?’ And one thing that was good about that—she was like, so, ‘You could go anywhere, and you come across something you’re allergic to’ or something that happens because that actually spikes my asthma.”  **[Patient Focus group #4]:** “For some people, you might need that reality check[ICS non-adherence may result in life-threatening event] because you might not take it as serious.”  **[Family/Friends group #5]:** “They [PCPs] should explain to them [patients] ‘Well, I told you that you need to do this and because you haven’t done this, these are the things that could take place’.” | Unequivocal |

Hannane A, Misane L, Devouassoux G, Colin C, Letrilliart L. Asthma patients' perception on their care pathway: a qualitative study. NPJ Prim Care Respir Med. 2019 Apr 2;29 (1):9.

| **Finding** | **Sub-findings** | **Illustration from study** | **Evidence** |
| --- | --- | --- | --- |
| The stakeholders of Patients | Experience | **P1:** “Everyday, actually, we learn to breathe that way; for us, it is no longer a discomfort.”  **P9:** “I’m not saying that I’m asthmatic but… it occurs periodically”  **P16:** “Anyway, I’m condemned.”  **P5:** “I see the doctor when I really have to, because [otherwise] it’s a waste of time for everyone.”  **P26:** “I saw some great professors, but nobody really knew how to cure this asthma.” | Unequivocal |
|  | Role | **P4**: “I am the first to be involved [in the management of asthma]!.” | Unequivocal |
|  | Difficulty | **P14**: “Unfortunately, I don’t know how to use them well [the inhalers] because I was not well  trained.” | Unequivocal |
| **Patient relationships with healthcare professionals** | Various types | **[Paternalistic approach]**  **P1:** “In fact, the pulmonologist, she was clear: “ <if you want to breathe better, that’s it. »A little closed on that, there was not much discussion to have.”  **P12:** “In fact, I don’t discuss it. When I am prescribed something, well, I take it.”  **P23**: “[what I would have liked during follow-up is that my GP] to have told me: Well, OK, you’re asthmatic, that’s how it’s going to be. [Here are] all the steps you will go through to check that everything is fine. Rather that than she takes care of me.”  **[Shared decision-making ]**  **P5:** “I like that doctors leave to the patient, [..] the appropriation of the disease and the treatment.”  **P20:** “I continue Airomir[salbutamol]. But later maybe I’ll stop… It depends… I will see the GP so that he explains how to do so.”  **P8:** “I have the advantage to have a homeopathic doctor, so it’s true that we have discussed from time to time to use homeopathy without ever really removing the long-term treatment that reassures me.” | Unequivocal |
|  | Communication | **[Failure]**  **P2**: “But I have not been explained things so much. But me, I need to understand.”  “I have experience of other specialist physicians and I do not find that they know how to explain things and to listen to their patients.”  **P22:** “Can asthma be controlled? Is it possible not to have it any more?”  **[Unsaid]**  **P14:** I have never dared to ask [how to use treatments], by shyness.” | Unequivocal |
|  | Dissatisfaction | **[Physicians]**  **P2:** “The regular physician had no blinkers; this is not the case for all [other physicians], I think.”  **[Disease control]**  **P16:** “I will be really satisfied the day when I will be told: “That’s it! We have found a cure. ”  **[Lack of recognition]**  **P1**: “I expect that she [my GP] takes my asthma seriously, because a substitute physician did not care whether I felt bad or not.” | Credible |
| **Interprofessional collaboration** | Experience | **[GP–specialist partnership ]**  **P12**: “When I went to the pulmonologist, it was on a doctor’s recommendation, so they should have communicated together.”  **P16**: “And then after a while, she [the pulmonologist] sends me back to my GB. Because, for her, I was not really a serious case, so… I think she actually only keeps serious cases.”  **P22**: “It would be good in the management of the medical follow-up that indeed there is a real communication between physicians. Is it possible and is it done, I don’t know. But that would be good.”  **P5:** “For me, it does not matter if it returns or it does not return [the communication between physicians]. In fact, it’s me who takes care of my health. So as long as I’m informed … Let’s say that I am the person responsible.”  **[CAM practitioners exclusion]**  **P8:** “The osteopath, if he has something to say, he will generally walk on eggshells because… there are very few physicians able to hear it. It’s very compartmentalized.”  **P2:** “I expect to be able to speak about that kind of treatment [CAM], which is not regarded as such by most of the medical profession, but which for me seems more than enough.” | Unequivocal |
|  | Benefits | **P24**: “Group work is to be favored, because it allows to have several views.”  **P5**: “I imagine that each time all test results are sent to the GP. That’s the principle.”  **P25:** “We have a patient interpretation of what the doctor has told us, and when we have to forward the information, sometimes we do not forward everything or we have forgotten part of it or we have misunderstood. And the fact that they directly communicate actually avoids these unintentional omissions and oversights.” | Unequivocal |
|  | Limitations | **P20**: “I don’t know. I think they do not really have time. Or they do not take time to do it.”  **P20**: “I have never been asked who was my pulmonologist or who was my GP; so no, they do not  communicate.”  **P28**: “A client record that would be accessible to all healthcare professionals, so that each professional can follow the records.” | Unequivocal |

Hoskins G, Williams B, Abhyankar P, Donnan P, Duncan E, Pinnock H, van der Pol M, Rauchhaus P, Taylor A, Sheikh A. Achieving Good Outcomes for Asthma Living (GOAL): mixed methods feasibility and pilot cluster randomised controlled trial of a practical intervention for eliciting, setting and achieving goals for adults with asthma. Trials. 2016 Dec 8;17 (1):584.

| **Finding** | **Sub-findings** | **Illustration from study** | **Evidence** |
| --- | --- | --- | --- |
| **Coherence: Meaning and sense making by participants** | Coherence: Meaning and sense making by participants | **After some initial uncertainty, many were able to set goals that ranged from everyday changes to more major challenges. The relevance of asthma to the goals was not always clear.**  **[Patient 901]:**”….it was quite clear and the way it then broke down so you actually really had to think about what it is that's limiting ….. it did actually make you think much more deeply about how you went about these things and therefore what were the key things that were holding you back.”  **[Patient: 103]:**”Yeah, it was easy to understand. The hard bit was to try and find an answer! ”  **[Patient 1002]:**”Oh it was good. It was about what I do, about my lifestyle. So, yes, it was ….pertaining to my life and what asthma had done to it. ….it just came out, just flowed out.”  **[Patient 901]:**”I thought it was very interesting, it was very difficult to work out what your goals were and then to think how asthma might affect them. Like, for instance, I've got ‘get fit’ there; I don't care about being fit, being fit is to enable me to do lots of other things, so I was getting confused with what was a goal, what did I really want to do and what were the mechanisms for getting there, which I think later on it pulled out, but on the first page I was getting very confused with those.” | Unequivocal |
| **Cognitive participation: Commitment and engagement by participants** | Cognitive participation: Commitment and engagement by participants | **Few patients volunteered for the study, perhaps reflecting unfamiliarity with the concept of goal setting, though 15 of the 18 patients participants remained committed throughout.**  **[Patient 101]:** “I have just finished university myself and had to do a research project and I'm more than happy to participate in anything that might make a difference to someone in the future.”  **[Patient 103]:** “I don’t mind taking part…I know when I had my pacemaker fitted I was asked a load of questions and I agreed…No, I think if I’m being helped then I return the help in some way … and I think that’s what one should do.” | Unequivocal |
| **Collective action: The work participants do to make the intervention function** | Collective action: The work participants do to make the intervention function | **Patients generally attempted to complete the pre-consultation goal setting exercise, but opinions about whether this was a useful task ranged from ‘insufficiently motivating’ ‘useful clarification’, ‘already clear about my goals’ ‘not sure I have/want goals’.**  **[Patient 107 ]:** “…… it gave me an opportunity to sit down and think things out a bit more clearly …… it made me prioritise much more……. I had a lot more aims and goals before I narrowed it down……” | Unequivocal |
| **Reflexive monitoring: Participants reflect on or appraise the intervention** | Reflexive monitoring: Participants reflect on or appraise the intervention | **The goal-focussed review was experienced as being more holistic, person-centred and partnership-based**  **[Patient 1002 ]:**”…it brought to life what…nobody else has ever asked. it …opened my eyes to my asthma and now I think I’m in control of it rather than asthma being in control of me.”  **[Patient 1201]:**”…basically what this has done is…reminded me that I don’t pay close enough attention to matching being good to myself, being kind to myself, with my conditions. And that I actually have to actively look after myself, not take it for granted, be sensible about using the medication appropriately, monitoring how I’m doing …[it] focused me on that.”  **[Patient 101 ]:**”I think it changed what we’d done in our asthma appointments ….because it gave it more of a focus…which is quite important.”  **[Patient 901]:**”…I thought it was very useful, it was like having a life coach on the NHS! It felt much more like a team, felt much more that she knows who I am and therefore we’re working together on my health rather than me doing what I'm told and being monitored.” | Unequivocal |
| **Implementation** | Implementation | **[Patient 901]:**”I suppose it’s difficult with studies but I suppose if it had been brought up rather than on quite a long letter, which it was wasn’t it, a reasonably long letter…and then there's forms to sign and there's a whole lot of barriers, and because it’s a study that's why it happens, but if it had been brought up perhaps when I’d gone in to see the nurse as a start off section, then that might have made people more responsive cause they didn't have to trawl their way through all the…words.” | Unequivocal |

Kopnina H, Haafkens J. Necessary alternatives: patients' views of asthma treatment. Patient Prefer Adherence. 2010 Jun 24;4:207-17

| **Finding** | **Sub-findings** | **Illustration from study** | **Evidence** |
| --- | --- | --- | --- |
| **Perception of illness**  **and patient identity** | Perception of illness  and patient identity | **[patient]**While one of the subjects described her symptoms as “acute” rather than chronic, she noted that the acute periods “sometimes do come back,” although she could not detect any specific patterns, such as environmental stimuli or triggers.  **[patient]**The majority of the subjects in the sample (n = 15) acknowledged that they “had asthma” and “were asthmatic”, while four subjects stated only that they “had asthma” and preferred not to “stick the sickness label” on themselves, as one of the subjects described it.  **[patient]**Most of the subjects (n = 17) agreed with the doctor’s diagnosis of asthma, although two subjects referred to the diagnosis made by the alternative practitioner (Chinese or Ayurvedic practitioner), as a “disease of the lungs” or an “imbalance”.  **[patient]S**even subjects experienced their asthma as something they should not discuss in public or at work due to the possible stigma of “being sick” or, as one patient put it, a “sign that you’re growing old”. However, all subjects except for two felt that it is “something that has to be dealt with” and “cannot be left untreated”. One patient remarked, self-deprecatingly, “Well, I just hope it will go  away”.  **[patient]**During the focus group session, the theme of negative stereotyping was expanded, with four of seven participants During the focus group session, the theme of negative stereotyping was expanded, with four of seven participants | Unequivocal |
| **Encounters with medical**  **practitioners** | Encounters with medical  practitioners | **[patient]**Experiences with medical practitioners varied greatly, from being confronted with a “very knowledgeable and attentive physician” to a “self-assured, know-little, so-called specialist”.  **[patient]**The patient reflected that it would only be a good compromise for the doctor herself, since by allowing the use of alternative medicine in a “complementary” way, the doctor made  sure that the patient stayed on “safe and proven” allopathic medicine. According to the patient, the doctor tried to “trick her”, refused to take her concerns seriously, and “completely  discounted the possibility of the effectiveness of alternative medication when taken on its own”.  **[patient]**The patient then mentioned her age of 29 years and that if she took her medication continuously as prescribed, by the age of 50 she “might be dead”.To this, the doctor, according to the patient, became “really upset” and talked to her “like she was a small, stubborn  Girl ”, speaking very slowly and distinctly, indicating that the patient ‘s concerns were “irrational”. The patient went for a second and third opinion and while she encountered  “more open-minded specialists”, she felt that her fears were not taken seriously.  **[patient]**a male patient with severe long-standing asthma (untreated for at least five years) revealed that his encounter with a doctor resulted in him “walking out of the doctor’s office” because the doctor “blamed (him) for not coming in earlier”, for “continuing to smoke while  knowing it is bad”, and for “not taking her (the doctor’s)opinion seriously”. The patient felt insulted by the doctor’s “arrogant attitude” and “complete lack of understanding”.the second doctor consulted by the same patient was”­well-informed” and “respectful” and suggested ­treatment in combination with lifestyle changes (quitting smoking). While the patient followed the recommendation and ­managed to quit smoking, he became concerned about the side effects of the prescribed medication and contacted the same doctor again with questions about alternatives. The doctor actually suggested that the patient should consider alternative medicine,  while warning him that “to the extent of his knowledge”, commonly prescribed medications “work best”. The doctor suggested, however, that he did not recommend alternative medications himself, and that if the patient chose alternative methods, he “will need to keep himself very well informed”and would be choosing CAM treatment “at his own risk”.  **[patient]**One patient in the group felt that “none of the doctors” she has consulted  had any respect for her concerns. This was echoed by two other women in the group, who considered that the doctors might have attributed their concerns to “female whims” or”hysteria”. This opinion was offset by the only male in the group, who said that his experience with doctors was just as humiliating and had nothing to do with gender. | Unequivocal |
| **Printed and online information** | Printed and online information | **[patient]**First, I was overwhelmed by the amount of data (on the Internet). Then, I was even more overwhelmed by the amount of conflicting data. And the advice people give (online) … some say ‘use it’, others say ‘no, never, it’s dangerous!’ … from what I could gather, it was clear that there is no agreed-upon opinion …  **[patient]**With most of them indicating that they encountered confusing data, some having “problems with understanding the (medical) jargon” and having trouble deciding which sources are “most reliable”. However, most of them indicated that on the basis of the information they found, they were able to make “an informed choice”. | Unequivocal |
| **Supplementary information**  **from patients** | Supplementary information  from patients | **[patient]**Ten indicated that the controversy around the safety of medicines containing budesonide and/or formoterol has been “particularly disturbing”.  **[patient]**As one of the subjects eloquently put it, referring to the others participating in the  study, “but then of course, we’re all skeptical asthmatics”. Another said “Well, that’s what you get with selection bias”.  **[patient]**One subject brought in the prescribing information with underlined passages from the warnings: “A 28-week, placebo-controlled US study comparing the safety of salmeterol with placebo, each added to usual asthma therapy, showed an increase in asthma-related deaths  in patients receiving salmeterol (13/13,176 in patients treated with salmeterol vs 3/13,179 in patients treated with placebo; relative risk 4.37, 95% confidence interval 1.25, 15.34). The  increased risk of asthma-related death may represent a class effect of the long-acting beta-2  -adrenergic agonists, including formoterol. No study adequate to determine whether the  rate of asthma-related death is increased with Symbicort has been conducted”.  **[patient]**The opinion of an overwhelming number (n = 17) of the subjects was that alternative medicines seemed “less threatening”, and that there were “no apparent risks”. While the data were also conflicting and patchy, most subjects felt that “once you’ve found the right source”. (ie, a source they could trust) it was “easy to navigate” and”find information”. | Credible |
| **Encounters with subjects’**  **social groups** | Encounters with subjects’  social groups | **[patient]**Eight subjects reported receiving  advice from other asthmatics, notably through Asthma Fonds.  Fellow asthmatics constituted a group whose “opinions were  Valued” or whose advice was sought as a complement to  other sources of information.  **[patient]**During the discussion, participants stated that while conventional asthma medications were geared towards relieving the symptoms of asthma, preventing their exacerbation, and preventing asthma attacks, they were not presented as “curing” asthma. All seven discussion participants stated their belief that medical practitioners (one patient included the “pharmaceutical industry” and “insurance companies”) insist that asthma is “chronic” and “incurable”by definition. The subjects reported the experiences of other asthma sufferers (friends, family members, or members of online forums), as well as the views of CAM practitioners (including TCM or Ayurveda) who maintain that asthma is not necessarily “incurable”. All seven participants mentioned that they “knew people” in their immediate social circle who were “cured of asthma” or at least no longer had asthma symptoms. When the moderator asked about how long these contacts had been symptom-free, and whether the symptoms had disappeared altogether, the responses were inconclusive.  **[patient]**Another important aspect of the positive experiences reported by the group using CAM was the belief that “CAM has no serious side effects” and that it is “safe to use”. When  the moderator asked subjects to elaborate on the evidence for this belief, the participants stated that “people they knew” had never reported any negative effects from TCM, Ayurvedic,  or homeopathic treatments. The only reported side effect of the use of certain herbs in TCM treatment was “excessive urination” which participants laughed at and did not find  threatening (“Not in comparison with heart attacks”, one of the participants said). | Unequivocal |

Lee, Deborah L, Hammond, John W, Finkel, Kelsey, Gardner, Donna D, Nelson, Belinda, Baptist, Alan P. An electronic shared decision-making app to improve asthma outcomes: a randomized controlled trial 2023 Jun 15: S2213-2198

| **Finding** | **Sub-findings** | **Illustration from study** | **Evidence** |
| --- | --- | --- | --- |
| **The ACTION app is an insightful communication tool about asthma** | The ACTION app is an insightful communication tool about asthma | [The App] slows me down, it makes me think about asthma.  It made me more aware of things I might need to bring up. Like okay, right now it’s allergy season. I’m  having to use my inhaler more.  It opened my eyes to maybe what the doctor might have been looking for that I may not have visualized. | Unequivocal |
| **The ACTION app and efficiency in the office** | The ACTION app and efficiency in the office | I didn’t think it took a lot longer. I mean, you know, she did read some of my comments and then comment  on them. And I would have asked those questions anyway. So, I thought it was possibly even shorter because she was already aware of my questions.  It gives you another tool to use. It just makes the visits more efficient.  It gives you a tool for having a relationship with your asthma specialists or your doctor | Unequivocal |

Melton C, Graff C, Holmes GN, Brown L, Bailey J. Health literacy and asthma management among African-American adults: an interpretative phenomenological analysis. J Asthma. 2014 Sep;51 (7):703-13.

| **Finding** | **Sub-findings** | **Illustration from study** | **Evidence** |
| --- | --- | --- | --- |
| **Information desired versus information received** | Information desired versus information received | **[Patients’ opinions about asthma education classes]**  Participant 1:If you got asthma,why not have a class and let people come there and get educated about asthma you know?  Participant 2 :No they never sent me to an educational class, but that probably would be good for someone like me that has the determination that they decide that I'm not gonna use that pump.’ Ok so a person like me should have already been in a class. It should have been something set out. You need to go to this class and see what happen to people when they don't use that pump every day.( **Social support**)  Participant 2 : What is asthma? What medications that they have on them arket for asthma. Uh, what uh triggers. What can trigger asthma? And what you need to do once you realize that you're having a asthma attack because I'm telling you, a lot of people don't know they having’a asthma attack.  Participant 1 who had previously attended an asthma education class suggested changes to the content saying:”In 2004, they didn’t really say how serious (asthma was). They let us know it (asthma) could get serious whereas everybody should know it’s a serious thing ... I mean people die have died from having asthma attack. It could get that bad.”  **[Other suggestions of patients to understand asthma]**  **Participant 2** said:‘‘I think it should be a pamphlet in every asthma doctor’ office; that if you goin’ to specialist, they need to have to give you so that you can read up on it ... and um,  I think they need to have in that pamphlet some foods that you need to avoid uh, when you have asthma.’’  **[Articipants with adequate print-related health literacy were more proactive than participants with low print-related health literacy in obtaining the information that they needed]**  **High health literacy**  **Participant 3** said:‘‘I have become computer literate. So I go down and look on the computer about asthma. And with me having it from 95 up until now, I uh, didn’t really have a source of information unless I got sick ... but now if there’s something new that I need to know, I keep in touch with what asthma is on the computer.’’  **Participant 2** said: ‘‘When I really learned about asthma,I learned it from going into the computer and pullin’ stuff out.’’ Although both participants relied heavily on computer  information, neither of them knew how to determine if the information was from a reliable source.  Participant 2 reported:‘‘If I’m reading something, and it’s some of the side effects I’m having, I believe it. And if it’s not, then I go back and ask my doctor.’’ When asked how she determined if the information she found on the computer was true.  **Participant 3** reported:‘‘Well to tell you the truth, I don’t cause it’s so much going on on the computer. And with me being the age I am, I am thankful that I can go in and put it on the uh on the website, and type in asthma uh asthma related, and they will go to the source.’’  **Low health literacy**  **Participant 4**:”I try to learn by reading what’s inside of the package when I open it. You have to be a mad scientist to really understand. But some of it, I kind of you know, you know, get the idea what they’re saying, you know... look like they writing to the doctor.” | Unequivocal |
| **Trial and error** | Trial and error | **Participant 3**:”I see that it’s a trial and error which you can’t really afford to have errors.”  **Participant 2** :‘‘Um most of the time, I feel like I’m in control. But when you have that bad asthma attack, no you’re not in control anymore. Asthma can kill you if you don’t manage it right.’’  **[Unlike the participants with adequate literacy, participants with low health literacy did not give a definitive yes about being in control of their asthma.]**  **Participant 1** said, ‘‘I mean I do what I’m supposed to do, and it’s been working pretty  good. When it [asthma] wants to cut up, it’s gonna’ cut up, and there’s no way I can uh stop it.’’ | Unequivocal |
| **Expectations of the patient–provider relationship** | Expectations of the patient–provider relationship | **[All participants expected their provider to listen to them]**  **Participant 2** reported, ‘‘I would just tell him [doctor]; I just want you to listen to me about  what’s going on with my body instead of writing while I’m talking to you. Look at me. Then I know that you listening to me.’’ Participant 4 said, ‘‘I didn’t feel like they were really interested. They were just talking. This is what they supposed to do, just talk. It wasn’t really concern.  **[Hope providers treat them as a whole person]**  **Participant 4**: ‘‘I just want my doctor to recognize who I am... and they say ‘‘well let’s see how you doing’’ you know.’’  **Participant 1** reported, ‘‘She [participant’s doctor] know pretty much what’s going on with me period. She knows with my body.’’  **[Hope providers to recognize their contributions]**  **Participant 2** said, ‘‘They’re in charge. I do what they say, but we have a good relationship. And I don’t care how long it takes. He knows he has other patients, but I will talk to him.’’  Participant 1: ‘‘If they [doctors] don’t agree with me, they’ll listen to me or whatever ... they’re going to run some tests to confirm what they think or to rule out what I think.’’  **Participant 2**:‘‘You gonna talk to me or you gonna put me on out, and I’m gonna go find me another doctor.’’  **Participant 3**:‘‘Except the last time I called to make an appointment, and then the receptionist was really awful. Well the heck with that. So I went to another doctor. They were good.’’  **[Responsibility to telling their providers all of their symptoms]**  **Participant 3**: ‘‘I’m gonna tell them everything.’’  **Participant 2**: ‘‘It is my responsibility to tell the doctor the truth.’’  **Participants with adequate print-related health literacy were more engaged as patients than participants with low health literacy. They believed it was important to bring information to their providers.**  **Participant 2** :‘‘I think today, they really look for you to go into the computer and come back and say something.’’  **Participant 3**:I know they know more than I do, but I share this information with them. What I have seen, and uh I tell them, ‘‘You know she gave me flower, and she gave me this, and she gave me that, but I find one of them to be very helpful.’’  **Participant 3** said, ‘‘I’m tellin’ them what’s going on with  me cause I know my body.’’ Participant 2 said, ‘‘It’s my  health. It’s not theirs.“Participant 2 said, ‘‘You got to have some uh uh focus  on what you want to happen to you with this asthma other  than waitin’ on your doctor, takin’ your hand, walkin’ us.’’  **[A good relationship with their providers was necessary for taking care of their asthma]**  **Participant 2**:‘‘You have to build rapport with your doctor. It come from building.’’ Participant 3 said, ‘‘The best thing to do is to have good communication with your doctor.’’  Participant 1 :‘‘I’m comfortable to the point where I don’t have a problem taking my medicines. If I’m not comfortable with my doctor, I’m not going to take the medicine.’’  **[Participants discussed several factors that impacted their relationships with their providers]**  **[mistrust]Participant 1**: ‘‘.. . My actual feeling about doctors are that they are in with the pharmacies .. . and it may not be something that you really need, but they got a contract or whatever.’’  **[race]Participant 4**:‘‘You hear how others they have done people in the past. You know, specially our people, you know. And uh, it make you wary. That’s the reason a lot of Blacks are wary about doctors.”This patient also recalled an experience where she felt the provider did not want to touch her because she was African-American. She reported:‘‘I have went to the doctor and he say ‘You got asthma?’Mmhmm that’s right. Uh pull that coat and then he got a ink pen and did something like he didn’t want to touch me .. . Maybe it’s a phobia or maybe you don’t like brown skin mmhmm.’’  **Participant 3**:‘‘I think when I first got pregnant and went to the doctor I was really timid ... you still had some of these prejudice white folks.’’ It is important to note that the participant whispered when she said ‘‘white folks’’ and would often touch and point to her skin instead of saying ‘‘black.’’  **[comfort]**a participant also felt discriminated against for being overweight, and she felt that providers have looked at her like she was “just disgusting”and attributed her asthma to her being  overweight.  **Participant 4** reported that discrimination is not always overt saying, “Yeah, yeah, suppose to it’s a lot of things they do behind closed doors, and what nobody there to witness, you know. You know, they say I never done that. I never said that, but you know deep down inside.”  **Both participants with adequate print-related health literacy discussed ways to overcome mistrust and discrimination.**  **Participant 2** emphasized the importance of building rapport with her doctor saying :‘‘You shouldn’t just have to build a rapport. It should be one when you get there, but in this society people look at the way you dress, your mannerisms. They look at your hair. They look at your eyes. They look at your everything, and that’s what they judge you by. They look at you don’t have no money. They look at your insurance. They look at everything. So you have to build rapport with your doctor.’’  **Participant 3** used information to combat negative experiences saying:‘‘All you wanna do is learn how to take care of yourself without asking anyone to help and I got to the point where  I was real smart .. . so I just started to researching a lot of stuff and that built up my confidence ...’’ | Unequivocal |

Mowrer JL, Tapp H, Ludden T, Kuhn L, Taylor Y, Courtlandt C, Alkhazraji T, Reeves K, Steuerwald M, Andrew M, Dulin M. Patients' and providers' perceptions of asthma and asthma care: a qualitative study. J Asthma. 2015;52 (9):949-56.

| **Finding** | **Sub-findings** | **Illustration from study** | **Evidence** |
| --- | --- | --- | --- |
| **Cost/Economic Barriers/Process** | Cost of prescriptions/lack of knowledge of insurance  rules | **[Provider]**: The aero chambers, some insurance companies cover them, a lot don’t. They are cost-prohibitive for us to just buy them because we can’t bill for them because the insurance won’t pay for them that way either. It’s figuring out creative ways for things that we know are good yet we are caught in the middle. We run into that big-time with inhaled medications. ð But if you have asthma, or chronic lung disease, and you need an inhaled medication, forget it.It’s terrible. You can’t find an inhaler control medicine for less than 90 bucks a month. That is the generic.  **[Provider]**: With everybody I’ve sat with, I give them the this is what’s out there, this is what your insurance will cover, this is what I’m willing to use but I really prefer this one over that one. But usually, from my experience, cost dictates.  **[Patient]**: I’ve been self-employed the last 4 years. I went through lay-offs. When I had to pay for Advair without a copay – I was livid. This is almost $400 and I need this to live? This is a really bad problem. So I got off of it and almost died. | Unequivocal |
|  | Medication choices | **Medication choice was an area of discussion, providers often had difficulty with the thought of giving choices to the patient and most patients did not seem to feel they had choices. Providers felt limited to the drugs covered by insurance carriers. As mentioned in the cost section, providers saw these limitations as barriers to offering choice of medication to patients.**  **[Provider on choice]**: Not a lot. I like to use generic medicines and not spend a whole lot of money. Let’s start with the most reasonably priced, most effective. There are not a lot of choices.  **[Patient]**: I would prefer having a choice.  **[SDM Patient]**: ‘I was the one that told them that the other medicine wasn’t working for me so, and they helped me find another medicine that would help me. Auxiliary and alternative choices (alternative therapies, breathing exercises, exercise, relaxation) | Unequivocal |
|  | Auxiliary and alternative choices (alternative therapies,  breathing exercises, exercise, relaxation) | **Most patients verbalized interest in alternative therapies, but a few patients actually practiced these therapies. Patients shared stories of herbal medicines, breathing techniques,**  **relaxation techniques and even exercises that helped them to improve their asthma and lung function. One of the positive consequences of these focus groups was that they provided shared educational time for stories and useful tips between patients and providers.**  **[Patient]:** I tried taking a supplement I’d heard about, some grape seed extract, aloe; it seemed to help. But when cats got around, after you’ve hit the albuterol twice you start to think that it isn’t going to work.  **[Provider]:** That is different for everyone and it is important to explore that. Yoga can be helpful; I’m a proponent of that, deep breathing. Your body is very different in how you react to those triggers. You’ve got to have that rescue. I’d be all for trying the remedies like eucalyptus, massage, acupuncture. I wouldn’t rely specifically on that especially in an emergency situation. | Unequivocal |
| **Self-Governance/Adherence.** | Asthma action plan | **[Patient]**: And once I showed my sister and them how to us the asthma action plan, they haven’t been hospitalized as much.  **[Nurse]:** It is a good idea to have an asthma action plan when you are in school in case your inhaler isn’t working for you. Everyone needs to know, not just the nurse.  **[Patien**t]: I don’t think there was anything as far as a plan. | Unequivocal |
|  | Goal setting | **[Patient]:** my goal is to be able to walk more and lose more weight, I’ve started a little bit, I’ve lost five pounds and um‘so far it’s just such a struggle, ‘cause you know, anyway my asthma this year has been trying to get me down and my body is- I’m wore out, I’m tired. Patient: I have to take care of family, so this is my goal, this is what I’m going to do. And I’ve got to find a good time of the day to walk because it is so humid. You know, if there’s a breeze blowing I’m outside because I enjoy the breeze, but I have to, again, limit myself so it’s just a little bit at a  time and going. You know something to work for.  **[Patient]:** Yep. What he did in my case, he, we set the goals,he actually said to me, we’re going to get you breathing easier, feeling better and that’s, so... and this is how we’re going to do it. And we just talked about it. And I went along those lines with him too. There’s everything, you have to keep a good attitude that has a lot to do with it. | Unequivocal |
|  | Lack of identity; not wanting to acknowledge disease | **[Patient]:** I have a question, is asthma like a disease?  Nurse: My goal is to get the students to take on the responsibility of understanding that they have a disease. |  |
|  | Triggers | **[Patient 1]**: I can walk around the corner to my daughter’s house which is no further than here to my car out there and by the time I get over there I’m puffing and panting. And if it’s real, real hot, it’s like if it’s real hot, it sucks the air right out of you.  **[Patient 2]**: Yeah, I become a prisoner in my own house, I feel like.  **[Patient 3]**: Yeah, but if it’s real cold, it seems like the cold sucks the air too.  **[Patient]:** I noticed that synthetic carpet, cats, dogs, lint, all the stuff like that triggers it.  **[Patient]:** And, I’m not really sure what really triggers my asthma, but I know there are a lot of triggers for me because dust I know is definitely one and that’s my biggest one. grass and it’s a lot of stuff outside, so one of the things that I’m supposed to do, but don’t do, is making everybody take off their shoes before they enter my house. I don’t even do it so, that’s one of my problems, but I’m pretty new to asthma, so I’m still trying to pretty much learn and feel my way through the whole ordeal. | Unequivocal |
| **Education** | Controller versus rescue inhaler | Conflicting views were presented regarding controller and rescue medications. Despite providers feeling that they were clear about the differences between these two types of medications, many patients did not know the difference between their controller and rescue inhaler. Some patients  used them interchangeably and others only used the one that seemed to work for them at the time.  **[Patient]**:I was like supposed to take so many puffs a day, and I over use it. | Unequivocal |
|  | Options | **[Provider]:** You know with our new EMR now, we can go and write a prescription and it will give a general idea whether its green, white, or red and so we can give them choices but some of those choices are limited based on formulary coverage.  **[SDM Provider]:** I think there’s different styles in medicine but what I’ve heard from other patients regarding our practices, is that it may not be formal shared decision making every time but we’re in a demographic that we’re the physicians. So, when you do give them a choice a lot of the times the choice is well you’re the doctor so you tell me what to do and so we start to learn our patients like who wants to be involved in that decision making. I always thinks it’s like a cut off at like somewhere 55–60 where, all the people 60 and above, they’re here for me to make the decision for them because that’s their train of thought, that’s their culture.  **[Patient]**: I feel like it’s best for you just to tell me what to do, cause I had no idea what asthma was. I just had it like maybe ð two or three years and I didn’t know and he told me, and that’s what I went with and, I trust him to tell me what to do. | Unequivocal |
|  | Techniques | **[Provider]:** Yea, maintenance therapy is a very hard concept to get over and then I just think most people don’t know how to use the inhaler either, so I try to go out of my way on explaining to them how, you know, how and I’ll ask them well you show me you know kind of an imaginary  inhaler and how you do it, and they go ‘spoosh spoosh’ and it’s like no that’s not how you have to use it. And I go through a full, uh.  **[SDM Patient]**: I had to change you know because I was using it the wrong way. But then when I went there and they taught me how to use it, I started to feel much better later on.  **[SDM Patient]**: I think it was a good experience because they taught me how to use my spacer. I had a spacer for my asthma, but I never used it before. They taught me how to use it so that it can help me and prevent me from having asthma attacks and stuff. It was a good visit for me.  **For the theme of Self-Governance/Adherence and Cost, we heard comments similar to this last patient’s comments around making medications last,**  No, it’s been a while. But one of my meds I cut in half because a 30 days supply is $75. There was no generic for that one. It was pill form. It is important for doctors to really understand that just because a pers. | Unequivocal |

Newcomb PA, McGrath KW, Covington JK, Lazarus SC, Janson SL. Barriers to patient-clinician collaboration in asthma management: the patient experience. J Asthma. 2010 Mar;47 (2):192-7.

| **Finding** | **Sub-findings** | **Illustration from study** | **Evidence** |
| --- | --- | --- | --- |
| **Personal Constraints** | Comorbidities | **[Patient]:** Celia divulged that she had diabetes and breast cancer. She said she had been seen in various clinics, including general medicine,30 times during the study year but only reported talking about asthma in a clinic visit once during an episode of acute symptoms. | Unequivocal |
|  | Health Beliefs | **[Patient]:**Linda reported “doing a ‘boot camp type training program’ and said the trainer will get her in shape so she doesn’t need any albuterol.” Kris “doesn’t want to take any more potent ICS (inhaled corticosteroids) (in spite of high peak flow variability) out of fear of weight gain.” | Unequivocal |
|  | Travel | **[Patient]:**Camille “just returned from a vacation where she did not take her prescribed ICS inhaler with her,” and then went to Europe where she “got sick with chest flu, congestion, and asthma.” A few months later Camille stayed in another state where her asthma symptoms worsened again. | Credible |
|  | Social Constraints | **[Patient]:**Judy was “stressed because of domestic violence issues with her boyfriend who “took or hid her ICS prior to her coming today.”Because of housing difficulties, Charlotte was living with friends  in another city where “she is sharing a home that has cats and her asthma has been worse.” | Credible |
| **Communication Failures** | Confusion or Lack of Comprehension Regarding S | **[Patient]:**Jane “is confused about her medicines and the meaning of her peak flow readings.” When asked why she thought her peak flow readings had improved, Jane replied “because I have asthma.”  **[Patient]:**She was seeing three physicians for her asthma and remained confused about most issues for the entire study period, although no signs of dementia or problems processing information were otherwise evident. | Unequivocal |
|  | Direct Communication with Clinicians | **[Patient]:**Ellen stated that “when she first saw her primary care provider (PCP) six years ago and he discovered that she had asthma since childhood, he dismissed discussion of the disease saying she must know all about that (asthma management) already and moved on,”and she did not ask for further guidance.  **[Patient]:**Daniel reported that he had “used Primatene mist for several years—using it 3–5×/day he says. By 10 am today he says he had taken 3–4 pfs. He says he thinks about asthma several times  a day—he says much of the time ‘every breath hurts’.” Nevertheless, Daniel did not mention his asthma symptoms to his PCP for 6 months. After reporting his symptoms to his PCP, Daniel continued to have asthma symptoms during the following months, including missing 10 days of work. He did not call his PCP or any health care provider. | Unequivoca |
| **Medication Issues** | Monitoring Adherence | **[Patient]:** Virginia, like many other patients, was chronically inconsistent in her use of preventive medication in the face of continuing and persistent symptoms. Without assessing or documenting Virginia’s adherence, her PCP increased her dose of the combination  ICS/salmeterol to the maximum level. | Credible |
|  | Access to Medications. | **[Patient]:** Leonard called the pharmacy to refill his fluticasone 2 puffs twice a day. He received fluticasone HFA labeled 2 puffs daily. He continued to take 2 puffs twice daily as “he believed they had used a much older script for renewal reference.” To Leonard this inconsistency did not seem worth discussing with either the pharmacist or the practitioner. However, shortly thereafter, he developed symptoms and noticed he was out of albuterol as well as fluticasone. He went back to the pharmacy and “they say that although he hasn’t used all his refills, the prescription for albuterol has expired and could not be refilled.” He was given 2 puffs of albuterol from a canister the pharmacist kept behind the counter and told that it would take up to a week to obtain his renewal because the clinic “doesn’t call back promptly.” | Credible |
|  | Inhaler Device Problems. | **[Patient]:**Patrick reported feeling better “because he now knows how to inhale medicine correctly.” Ellen, a normal size adult, was inappropriately using a pediatric mask and spacer with her inhaler. | Unequivocal |
| **Health Care System Barriers** | Clinic Bureaucracy | **[Patient]:**Sylvia “was to get allergy shots if insurance paid for them—the clinic was to call back with information regarding insurance approval, but hasn’t.” The clinic still had not called with this information 12 months later, and the patient never had an allergy consultation. | Unequivocal |
|  | Care Discontinuity | **[Patient]**: When Betty brought her medications to the first study visit, she had budesonide, triamcinolone, and a combination inhaler with fluticasone and salmeterol. She did not use any of them regularly during the entire study period. | Credible |

Norful AA, Bilazarian A, Chung A, George M. Real-world Drivers Behind Communication, Medication Adherence, and Shared Decision Making In Minority Adults with Asthma. J Prim Care Community Health. 2020 Jan-Dec;11:2150132720967806.

| **Finding** | **Sub-findings** | **Illustration from study** | **Evidence** |
| --- | --- | --- | --- |
| **ICS misuse and lack of knowledge** | ICS misuse and lack of knowledge | “I’ve been taking my Albuterol 200 times per day and there is no improvement”  “I didn’t realize [ICS use] was mandatory. I thought I just take it when I’m short of breath”  “I only take [ICS] when my body says I’m going to fall over and I can’t breathe.”  “I didn’t know that it is prescribed for twice a day. I just use it when I am short of breath. Other times I go through an inhaler a day.” Similarly, another patient said, “I only take it when my body says I’m going to fall over, and I can’t breathe.”  “I was taking my albuterol up to 8 times a day and nothing was helping until I ended up at the hospital.”  “I don’t usually take my [brand name ICS] until I take the albuterol a few times and see if it slows down [my symptoms]. If not, then I take it.”  Some patients were unfamiliar with how to recognize worsening asthma symptoms. “I just keep coughing. I’m always catching a cold.”  “The exacerbation made me insecure [about asthma management] . . . an eye-opening experience,” | Unequivocal |
| **External influences yielding personal misconceptions** | External influences yielding personal misconceptions | “What I hear on TV is really scary. I rather not take my inhaler than get those illnesses.”  “What are they doing to us? I asked Google and YouTube and then watched a video all about it [side effects].”  “[ICS brand name] is only prescribed by companies to make money.”  “The more you take something you get immune to it. That’s why they kept upping my asthma medication.”  Other patients were concerned that ICS use weakened their health. “I think I started getting pneumonia from [brand ICS]. Every time I take it, I get sick.”  “I see all these medicines on TV. This causes this and that one causes gangrene. I rather go through what I have than risk that.”  “That’s what you hear on the news all the time, you [provider] write a prescription, you get paid.” | Unequivocal |
| **Patient-provider communication to**  **individualize plan of care** | Patient-provider communication to  individualize plan of care | **[Patient]:** “Scared of the side effects she heard on tv.”  **[Physician]**: “I understand where you are coming from. I will tell you that the side effects of the inhalers are minimal, and the risk of uncontrolled asthma is a lot worse.”  **[Patient]:** “I’m not giving up my pets, but I didn’t know I can do things to help not get so sick from being around them.”  **[Patient]:** “It’s much easier for me for jump on the bus and go across the city to that location than find a ride to an office downtown.”  **[Patient]:** “To be able to play with the kids outside without getting short of breath. I need to keep up with them.”  **[Patient]:** “My daughter takes me to all my appointments. If she can’t go, I can’t go.” | Credible |

Tapp H, Kuhn L, Alkhazraji T, Steuerwald M, Ludden T, Wilson S, Mowrer L, Mohanan S, Dulin MF. Adapting community based participatory research (CBPR) methods to the implementation of an asthma shared decision making intervention in ambulatory practices. J Asthma. 2014 May;51 (4):380-90.

| **Finding** | **Sub-findings** | **Illustration from study** | **Evidence** |
| --- | --- | --- | --- |
| **Education** | Education | **[patient]:**”You freely can ask questions and they answer them and they show you things and they tell you about the breathing and the mucus building up in your lungs and you know it helped me…”  **[patient]:**”I had a spacer for my asthma, but I never used it before but they taught me how to use it so [the medication] can help me more…to prevent having the asthma attacks .. so, it was a good visit for me.”  **[patient]:**When I went there they told me that I was not using the right medication at the right time.… because I was using it the wrong way. But then when I went there and they taught me how to use it, I started to feel much better later on.”  **[The SDM process]:**  **[patient]:**”I feel great… I’m doing a lot better than I was before.…all the medicine that wasn’t helping me…it was just a waste of time. I feel good being a part of my decision of my medication.”  **[patient]:**”When I moved here, I was on a lot of asthma medicines [list of medications]. We came to a lot of decisions over my medication.” | Unequivocal |

Tapp H, Derkowski D, Calvert M, Welch M, Spencer S. Patient perspectives on engagement in shared decision-making for asthma care. Fam Pract. 2017 Jun 1;34 (3):353-357.

| **Finding** | **Sub-findings** | **Illustration from study** | **Evidence** |
| --- | --- | --- | --- |
| **Lived experience patients** | Lived experience patients | **[A health care professional with Asthma]:**I have been a patient with asthma for over 20 years and am grateful to be very well controlled. I have a vested interest in helping people with asthma. I hope to be an inspiration, advocate, and role model to others with asthma and show that an active lifestyle is definitely possible. Volunteering with researchers is a positive experience. I have been involved for more than five years now in many aspects of the project. I have attended four national meetings, including a patient engagement in research conference, and have engaged with other patient partners to share our experiences and learn from others.  **[Patient thoughts]:** I was diagnosed with asthma in my early 20s. I am a very well-controlled asthma patient and I am able to lead a very active lifestyle, including running several marathons.  Specific examples of patient input: this patient was instrumental in pre-award idea development and present throughout research project. Recommended that patient survey should be shortened to one question. Suggested changes to simplify wording on toolkit. Commented on progress of all aspects of project. | Unequivocal |
| **Caregiver advocates** | Caregiver advocates | **[Patient thoughts]:** ‘With all that I have gone through with caring for my children with asthma, it was an easy choice to engage with the team. I have become more engaged in raising asthma awareness. Each year, on the anniversary of my daughter’s birthday, I ask people to post an inspiring story with [a dedicated] hashtag in memory of my daughter who passed away from asthma’.  Specific examples of patient input: training in quantitative analysis by research team. Analysis of patient focus groups looking for themes and subthemes. Member of other advocacy groups such as PCORI Evidence to Action Network, Tobacco Free Mecklenburg and Mecklenburg County Asthma Coalitions (MCAC), developed video of patient engagement role for PCORI and gave input into posts on the MCAC website and social media pages. Suggested setup of Instagram account and short videos on Periscope. Shares information on personal Instagram, Facebook and Twitter accounts. Along with having brochures or pamphlets in patient waiting rooms, suggested making brochures available in Mecklenburg County recreation centres. Wrote and published book on experiences of having children with severe asthma. | Unequivocal |
| **Research participants** | Research participants | **[Patient thoughts]:**  I appreciate being a part of the phone call. I am able to better understand the complexities of implementation, and as a patient, I can help guide the practices in thinking about how patient schedules are set up and to take into account the patient’s point of view and the staff’s needs when implementing visit schedules.  Specific examples of patient input: gave input into qualitative themes chosen by the research team to ensure they were patient centred. Monitored calls and commented on the use of facilitators and spread of intervention. Commented on the major themes that emerged from the call. Emphasized the need to address school calendar, winter flu season and spring allergy season in asthma visit scheduling. | Unequivocal |
| **Patient advisory board** | Patient advisory board | **[Patient thoughts]:**  Comments given: the shared decision-making tool is important for us to better understand our treatment plan and be involved in the decision with the provider. We appreciate the study will help understand how facilitators can affect implementation. This study also allowed us give input on another shared decision-making initiative that helps elderly patients decide to discontinue medications.  Specific examples of patient input ‘around dissemination strategies’: find smiley, approachable people for dissemination who are trusted by both patients and medical community and totally committed to sit at tables and network at community events. Repetition of the message is important. Magnets and bracelets can be useful as a signal of common support. Brochures should be in the exam room as well as waiting rooms. Patients would like to take them away for distribution to friends and family. A trifold brochure was felt to be most effective printed material. Patients do want study results as long as they are simply stated. Make sure the magic word ‘asthma’ is first, large and bold on the brochure. For example, ‘Do you have asthma?’ Be careful of acronyms. Have a central place to explain abbreviations on documents. One patient suggested the brochure be attached to other discharge material or attached to pre-visit material such as the asthma control survey in medical office visits. | Credit |

Young HN, Havican SN, Griesbach S, Thorpe JM, Chewning BA, Sorkness CA. Patient and phaRmacist telephonic encounters (PARTE) in an underserved rural patient population with asthma: results of a pilot study. Telemed J E Health. 2012 Jul-Aug;18 (6):427-33..

| **Finding** | **Sub-findings** | **Illustration from study** | **Evidence** |
| --- | --- | --- | --- |
| **Positive and very helpful** | Positive and very helpful | **[Patient]**: ‘‘I think that that was a very good experience. She was very helpful.’’  **[Patient]**: ‘‘I thought my experience was very good. I felt comfortable talking with her about my problems or how I was doing with my asthma.’’  **[Patient]**: ‘‘She was very conscious of my needs and very friendly.’’ | Unequivocal |
| **Improve**  **self-management** | Improve  self-management | **[Patient]**: ‘‘I think just understanding things better and finding easier ways for me to take what I need and still make sure I’m doing the right thing to manage it.’’  **[Patient]**: ‘‘It’s better now. There are things that I didn’t realize before that I do now. So, you know like about shaking it up and rinsing it out if I haven’t used it in a while and things like that. I definitely learned some things from it.’’  **[Patient]**: ‘‘Well, it really helped out a lot because I learned some better ways to rinse my inhaler, how to use, how many seconds to hold my breath. It’s really helped with my quality of breathing tremendously.’’ | Unequivocal |
| **Questions and**  **immediate feedback** | Questions and  immediate feedback | **[Patient]**: ‘‘Very informative and she let me ask questions and she took the time to answer so that I could understand. She would ask me if I understood what she was explaining and it was really nice.’’  **[Patient]**: ‘‘I like having that immediate feedback if I had any questions or comment about my asthma. She was there to answer it right away and if she didn’t have an answer, she would find one and get back to me as soon as possible.’’  **[Patient]**: ‘‘Just the fact that they were open to looking at different things for me to take because I am like  the person who does not want to be on the wrong medication or be like guinea pig. That we went  the one direction I wanted to go.’’ | Unequivocal |
| **Time** | Time | **[Patient]**: ‘I don’t know. Sometimes you know you have to do things, usually I told her so it was okay really, it worked out okay.’’  **[Patient]**: ‘‘Just that it was time consuming but you know that’s okay that was no big deal.’’ | Unequivocal |
