# Supplementary material for: The experience of shared decision‐making for people with asthma: A systematic review and metasynthesis of qualitative studies
Source: Health Expect. 2024 Apr 13;27(2):e14039. doi: 10.1111/hex.14039 (PMC11015866; doi:10.1111/hex.14039)
Supplement: Supplementary file 5 — Supporting information. [file HEX-27-e14039-s006.docx]

**Appendix 5: Results of meta-synthesis**

| **Findings** | **Items** | **Category** | **Synthesized findings** |
| --- | --- | --- | --- |
| **Synthesized findings 1: Adult asthma patients’ capability** | | | |
| Don’t really know about their choice(U) | **49** | **Attitude toward SDM** | Capability is defined as an individual's cognitive and physical capacity to engage in activities, encompassing the necessary knowledge and skills. It is important to recognize that the abilities of adults with asthma have a profound impact on participation in SDM programs and participant experience. Patients' attitude towards SDM (include decision aid), ability to acquire asthma-related knowledge and understanding ability are important factors affecting the ability of adult asthmatic patients to participate in SDM. |
| Don’t interest about SDM(U) |  |  |  |
| It's important to share decision with my doctor(U) |  |  |  |
| The decision should be made by doctors(U) |  |  |  |
| Information, doctors’ attitude, Continuity of care, assertiveness of patients are important to make decision(C) |  |  |  |
| Nurses should take part in SDM(U) |  |  |  |
| The severity of the disease affects my decision-making power(U) |  |  |  |
| The choice provided by doctor is good(U) |  |  |  |
| You don't know what your decision-making power is(U) |  |  |  |
| The implementation of SDM should be candid(U) |  |  |  |
| Physicians should be trained in SDM to provide better decision making（U） |  |  |  |
| Commend the way doctors communicate about SDM(U) |  |  |  |
| Recognition of SDM(U) |  |  |  |
| Having a decision discussion with my doctor is reassuring(U) |  |  |  |
| Favour the inclusion of multidisciplinary teams(U) |  |  |  |
| Teamwork can prevent forgetting things(U) |  |  |  |
| Willing to participate in SDM(U) |  |  |  |
| SDM makes me facous(U) |  |  |  |
| Being correct is great(U) |  |  |  |
| Reluctant about decision making(U) |  |  |  |
| I want to take part in SDM and know the risks(U) |  |  |  |
| I can accept anything(U) |  |  |  |
| I wish they can agree my opinion(U) |  |  |  |
| I like communicate about the decisions(U) |  |  |  |
| Communication can give a more holistic picture of health throught the pregnancy(U) |  |  |  |
| They support my decision(U) |  |  |  |
| Glad to join my medicine decision(U) |  |  |  |
| The SDM visits is of great helpful(U) |  |  |  |
| The CDSS is an interesting wool(U) |  |  |  |
| The CDSS is helpful(U) |  |  |  |
| The CDSS is vision friendly(C) |  |  |  |
| The CDSS is not the best because it’s just a paper(U) |  |  |  |
| The CDSS is good but I prefer to communicate with my specialist(U) |  |  |  |
| Decision aid is helpful to rank my priorities(U) |  |  |  |
| The decision aid is too detailed(C) |  |  |  |
| I dislike the text-based tool(U) |  |  |  |
| Visual thing is always helpful(U) |  |  |  |
| It can help to faufe how much medication I can inhale(U) |  |  |  |
| Decision aid is too long(U) |  |  |  |
| One page is great(U) |  |  |  |
| An email reminder will be good(C) |  |  |  |
| The decision is prepare for whom?(U) |  |  |  |
| I like teamwork for health management(C) |  |  |  |
| Nurses are better than decisions aid(U) |  |  |  |
| It opened my eyes(U) |  |  |  |
| It connected me and specialists(U) |  |  |  |
| Brosure should put in exam room and waiting room(C) |  |  |  |
| I prefer to spend more time on one on one take(U) |  |  |  |
| SDM is positive and very helpful |  |  |  |
| Knowledge about asthma is helpful(U) | **66** | **Asthma knowledge acquiring, understanding and processing** |  |
| I want more information to make decision in SDM to make decision by myself as well as my provider(U) |  |  |  |
| Hope nurses explain things in a better way(U) |  |  |  |
| Specialist give me the cortisone without a second thought(U) |  |  |  |
| Information is too detailed to read(C) |  |  |  |
| It is worthy to know from a nurse practioner(U) |  |  |  |
| The information from nurse practioner and my doctor confuse me(U) |  |  |  |
| It’s good to know I can continue to take medicine(U) |  |  |  |
| I don’t like find information online(U) |  |  |  |
| Don’t believe I got asthma but the internet prove it maybe true(C) |  |  |  |
| Everyone has their own opinion online(U) |  |  |  |
| Internet information overwhelmed me(U) |  |  |  |
| Problems with understanding the (medical) jargon on line(U) |  |  |  |
| Don’t have reliable information source except check online(U) |  |  |  |
| I will check online when I need asthma information(U) |  |  |  |
| Easy to manipulate and get information online(U) |  |  |  |
| The information from TV is scary(U) |  |  |  |
| Side effects about ICS is scary in TV(C) |  |  |  |
| Check on google first, if it still confused, the trying to ask doctor(U) |  |  |  |
| Information from mother or grandmother is helpful(U) |  |  |  |
| Turn fan on to control asthma(U) |  |  |  |
| People with asthma form a community(C) |  |  |  |
| Information from social circle that somebody were “cured of asthma”(C) |  |  |  |
| Group discussion abpuot their experience(U) |  |  |  |
| Not paying attention to the leaflet(U) |  |  |  |
| Digital person hates paper(U) |  |  |  |
| The piece can tell me medication information(U) |  |  |  |
| Don't understand the instructions(U) |  |  |  |
| Scientific studies make me anxiety(U) |  |  |  |
| Be brave enough to speak up about health issues that are not being noticed by your provider(U) |  |  |  |
| Do what I was asked(U) |  |  |  |
| I finally realized using albuterol can be damage(U) |  |  |  |
| Regular go to hospital to check out(U) |  |  |  |
| Unauthorized changes in drug use(U) |  |  |  |
| Ask doctor if I can stop Airmomir(C) |  |  |  |
| Think about what hold me back(U) |  |  |  |
| Easy to understand, hard to try(U) |  |  |  |
| Make my mind more clear(U) |  |  |  |
| Only trust the information what I get through, or I will ask doctor(U) |  |  |  |
| I did what I can do(U) |  |  |  |
| Be willing to communicate with doctor(U) |  |  |  |
| Tell the doctor everything(U) |  |  |  |
| Tell doctor the medicine may not work(U) |  |  |  |
| Fight for families(C) |  |  |  |
| Keep good mood with asthma(U) |  |  |  |
| Knowledge about triger(U) |  |  |  |
| Doing the right things to manage it(U) |  |  |  |
| Don’t know how to use reliever(U) |  |  |  |
| Don’t know much about asthma(C) |  |  |  |
| Control asthma is impossible(U) |  |  |  |
| Doctor knows better than me(U) |  |  |  |
| Patient with long term experience can control asthma better(C) |  |  |  |
| Lack of knowledge(C) |  |  |  |
| Not taking the medication as prescribed(U) |  |  |  |
| Common to forget doses of ICS(U) |  |  |  |
| Fear of ICS(U) |  |  |  |
| Don’t take medicine to control asthma but take a bath(U) |  |  |  |
| Do not use ICS as prescribed(U) |  |  |  |
| Sometimes use intranasal steroid(U) |  |  |  |
| ICS non-adherence may result in life-threatening event(U) |  |  |  |
| Dared to ask how to use treatments by shyness(U) |  |  |  |
| Don’t know how to start an action(U) |  |  |  |
| Making asthma action plan is good to control(U) |  |  |  |
| Don’t know the trigger about asthma(U) |  |  |  |
| Don’t call PCP to tell symptoms(U) |  |  |  |
| **Synthesized findings 2：Opportunities of the adult asthma patients with SDM experience** | | | |
| I want to know how many people will die from asthma(U) | **15** | **Offering choices tailored to the needs of the patients** | Opportunity is defined as all factors external to the individual that enable or promote behavior. For asthma patients, it is important to provide them with opportunities to participate in SDM. For asthma patients, it is important to provide them with opportunities to participate in SDM. Which means engaging in thorough discussions to understand the pros and cons of various decisions as a prerequisite for decision-making. This includes offering choices tailored to the needs of the patients and institutional problems in the medical system. |
| Know the side-effect of each medication is necessary to choose(U) |  |  |  |
| I want to know everything instead of being taken care(U) |  |  |  |
| They don’t know how to explain and listen(U) |  |  |  |
| I need to understand(U) |  |  |  |
| I don’t really know how decision-aid working(U) |  |  |  |
| Specialist don’t know how to explain and listen(U) |  |  |  |
| No body tell me how to use inhalers(U) |  |  |  |
| Don’t discuss asthma but take medicine(U) |  |  |  |
| Don’t discuss asthma but take medicine(U) |  |  |  |
| Knowledge support is needed(U) |  |  |  |
| Feel welcomed by others in communication(U) |  |  |  |
| Doctor always there to help me(U) |  |  |  |
| Talk to me or run me out(U) |  |  |  |
| Nobody really knows asthma(U) |  |  |  |
| Sick leave is not enough(U) | **27** | **Institutional problems in the medical system** |  |
| Work hard even sick(U) |  |  |  |
| Stop work makes me reduced(U) |  |  |  |
| Cost is a valid criteria for anybody's decision-making(U) |  |  |  |
| Planning vacation is not given(C) |  |  |  |
| Seeing doctor is waste of time(U) |  |  |  |
| Advair is expensive to pay(U) |  |  |  |
| Cut my meds half because it is expensive(U) |  |  |  |
| Housing does not provide a good environment(U) |  |  |  |
| I can’t go to my appointments if my daughter can’t go(U) |  |  |  |
| It’s not easy to get to clinic(C) |  |  |  |
| Can I afford this medication?(U) |  |  |  |
| I wish that healthcare centres make educational programs regularly(U) |  |  |  |
| Accidentally been diagnosed(U) |  |  |  |
| No concrete diagnosis(U) |  |  |  |
| Treat me like an animal(U) |  |  |  |
| Health professionals lack of SDM knowledge(C) |  |  |  |
| Use of lay terms and simple language(U) |  |  |  |
| Language problem(U) |  |  |  |
| Not much discussion(U) |  |  |  |
| Interruption of care(C) |  |  |  |
| Most of the medical profession don’t support CAM(C) |  |  |  |
| Many people don’t know they have asthma(U) |  |  |  |
| Clinic not called with information (U) |  |  |  |
| Not use any of medicine regularly during the entire study period(U) |  |  |  |
| Doctor relied on me to ask(U) |  |  |  |
| Decision aid can involve in the decision with the provider(U) |  |  |  |
| **Synthesized findings 3: Motivation of the adult asthma patients with SDM experience** | | | |
| Always go to hospital for nebulizer makes me fears and anxiety(U) | **76** | **Emotions: pleasant vs unpleasant experiences,** | Motivation is defined as the cognitive processes within the brain that encompass all incentives and guiding factors behind behavior, encompassing not only goals and conscious decision-making. Factors inherent to the patients themselves may impede their engagement in SDM. In the case of asthma patients, their motivation to participate in SDM is primarily influenced by their own emotions (pleasant vs unpleasant experience) and Self-efficiency. |
| It’s embarrassing me to use it in front of others(U) |  |  |  |
| I’m worry about emergency(U) |  |  |  |
| Happy about what doctors did(U) |  |  |  |
| The doctors trying to get me out(U) |  |  |  |
| I have faith in the doctor(U) |  |  |  |
| I am happy to take all treatments provided by doctor(C) |  |  |  |
| Go to a doctor is a form of worship(C) |  |  |  |
| I wouldn’t expect GP to be a specialist(U) |  |  |  |
| GP has got a limited knowledge(U) |  |  |  |
| Training GP to understand what I mean(U) |  |  |  |
| All doctors aren’t good doctors(U) |  |  |  |
| Just say, don’t listen(U) |  |  |  |
| A little closed about discussion(U) |  |  |  |
| Can asthma be controlled(U) |  |  |  |
| I hope GP takes me seriously(U) |  |  |  |
| Pulmonologist should take continouse care(U) |  |  |  |
| Pulmonologist only keeps serious cases(U) |  |  |  |
| GP should be sent all teat results(U) |  |  |  |
| Experiences with medical practitioners varied greatly(C) |  |  |  |
| Doctors trying to cheat her(C) |  |  |  |
| A doctor blamed him(C) |  |  |  |
| A doctor respect me and suggest me good(C) |  |  |  |
| Doctors attributed their concerns to “female whims” or “hysteria”(C) |  |  |  |
| Hope doctor can be listening instead of wrighting(U) |  |  |  |
| They are not really care about me(U) |  |  |  |
| Just want my doctor recognize who I am(U) |  |  |  |
| Doctor knows pretty much about me(U) |  |  |  |
| Doctors will do some test and show me if they don’t agree with me(U) |  |  |  |
| Doctors know more than me so I will share, and they will help(U) |  |  |  |
| You have to build rapport with your doctor(U) |  |  |  |
| I’m not going to take the medicine if I feel bad about my doctors(U) |  |  |  |
| Doctors are in with pharmacies and got a contract(U) |  |  |  |
| They don’t want to touch because I am a black(U) |  |  |  |
| Being discriminated to be overwight(C) |  |  |  |
| PCP refuse to discuss so I choose not ask(U) |  |  |  |
| Nurse line got tired about my calling(U) |  |  |  |
| I don’t understand the jargon(U) |  |  |  |
| My doctors always listen to me instead of typing(U) |  |  |  |
| They always there to listen when communication even they don’t agree(U) |  |  |  |
| I prefer do what they ask(U) |  |  |  |
| I don’t know my symptoms(U) |  |  |  |
| Now I know how to use it(U) |  |  |  |
| Doctor is informative and patient to communicate with me(U) |  |  |  |
| They were open to looking at different things for me(U) |  |  |  |
| I just want to be left alone(U) |  |  |  |
| I’m not saying I’m asthmatic(U) |  |  |  |
| Preferred not to “stick the sickness label”(C) |  |  |  |
| referred to the diagnosis as a “disease of the lungs” or an “imbalance”(C) |  |  |  |
| Stigma of “being sick”(U) |  |  |  |
| Change a doctor if I was not satisfied with the doctor(U) |  |  |  |
| Communication is not official so it is a long process(U) |  |  |  |
| Commonly held pre-existing beliefs about each therapy(U) |  |  |  |
| ICS caused weight and didn’t solve problem(C) |  |  |  |
| Prednisone is not really good(U) |  |  |  |
| ICS is evil(U) |  |  |  |
| Encounters with medical practitioners and not understand my fear(U) |  |  |  |
| Prescribing information with underlined passages(U) |  |  |  |
| Asthma-related death is increased with Symbicort has been conducted |  |  |  |
| It’s a trial you can’t afford(U) |  |  |  |
| Receptionist was really awful(U) |  |  |  |
| Auxiliary and alternative choices(U) |  |  |  |
| Over use inhaler(U) |  |  |  |
| Bad health beliefs about albuterol(U) |  |  |  |
| ICS misuse and lack of knowledge(U) |  |  |  |
| External influence (ICS) yielding personal misconceptions(U) |  |  |  |
| Pregnancy complications(U) |  |  |  |
| Impact on lactation(U) |  |  |  |
| Effects of not taking the medication(U) |  |  |  |
| Alternative treatments(U) |  |  |  |
| Exhibits empathy(U) |  |  |  |
| Awareness of medications(U) |  |  |  |
| I know myself so I don’t make asthma plan(U) | **16** | **Different lifestyle** |  |
| I need to think about the medicine which doctors doesn’t concern(U) |  |  |  |
| It’s my body and my life(U) |  |  |  |
| My autonomy eventually makes me don’t have any choice(U0 |  |  |  |
| I can’t have budgies but I still did(U) |  |  |  |
| To be a chef is harmful to my body but I insist to be(U) |  |  |  |
| Maintaining valued roles(U) |  |  |  |
| I am doing what I love which can’t take it away except I die(U) |  |  |  |
| Searching for normality(U) |  |  |  |
| I’ve still got as normal a life as I can(U) |  |  |  |
| Priorities for comparisons between therapeutic options(U) |  |  |  |
| Healthy lifestyle(U) |  |  |  |
| Coherence: Meaning and sense making by participants(U) |  |  |  |
| Expectation of the patient-provider relationship(U) |  |  |  |
| Travel(U) |  |  |  |
| Know more and it makes you feel more responsible for your life(U) | **9** | **Self-effciency** |  |
| Take medicine were perceived as enablers to control life(U) |  |  |  |
| Happy about healthy lifestyle(U) |  |  |  |
| Learn to breathe everyday is no longer a discomfort(U) |  |  |  |
| I am the first to be involved(U) |  |  |  |
| Reflexive monitoring brought to life(U) |  |  |  |
| Help me focused me on that(U) |  |  |  |
| I’m in control(U) |  |  |  |
| Lived experience patients(U) |  |  |  |
