# Supplementary material for: The experience of shared decision‐making for people with asthma: A systematic review and metasynthesis of qualitative studies
Source: Health Expect. 2024 Apr 13;27(2):e14039. doi: 10.1111/hex.14039 (PMC11015866; doi:10.1111/hex.14039)
Supplement: Supplementary file 6 — Supporting information. [file HEX-27-e14039-s003.docx]

**Appendix Ⅵ：ConQual summary of findings**

| **Systematic review title:** Shared decision-making for people with asthma as perceived by patients: A systematic review and meta-synthesis of qualitative studies  **Population:** Adult asthmatics with shared decision-making experience  **Phenomena of interest:** Perspectives and experiences of asthmatic patients participating in shared decision-making decision making  **Context:** The context include home, clinic, community, internet, telephone | | | | |
| --- | --- | --- | --- | --- |
| **Synthesized finding** | **Type of research** | **Dependability** | **Credibility** | **ConQual score** |
| **Synthesized findings 1: Adult asthma patients’ capability:**  Capability is defined as an individual's cognitive and physical capacity to engage in activities, encompassing the necessary knowledge and skills. It is important to recognize that the abilities of adults with asthma have a profound impact on participation in SDM programs and participant experience. Patients' attitude towards SDM (include decision aid), ability to acquire asthma-related knowledge and understanding ability are important factors affecting the ability of adult asthmatic patients to participate in SDM. | Qualitative | Downgrade  1 level* | Downgrade  1 level** | Moderate |
| **Synthesized findings 2: Opportunities of the adult asthma patients with SDM experience：**  Opportunity is defined as all factors external to the individual that enable or promote behavior. For asthma patients, it is important to provide them with opportunities to participate in SDM. Which means engaging in thorough discussions to understand the pros and cons of various decisions as a prerequisite for decision-making. This includes offering choices tailored to the needs of the patients and institutional problems in the medical system. | Qualitative | Downgrade  1 level* | Downgrade  1 level** | Low |
| **Synthesized findings 3: Motivation of the adult asthma patients with SDM experience**  Motivation is defined as the cognitive processes within the brain that encompass all incentives and guiding factors behind behavior, encompassing not only goals and conscious decision-making. Factors inherent to the patients themselves may impede their engagement in SDM. In the case of asthma patients, their motivation to participate in SDM is primarily influenced by their own emotions (pleasant vs unpleasant experiences) and Self-efficiency. | Qualitative | Downgrade 1 level* | No change | Moderate |

*Downgraded one level due to common dependability issues across the included primary studies (the majority of studies did not present a statement locating the researcher culturally or theoretically, and there was no acknowledgment of their influence on the research).

**Downgraded one level to a mix of unequivocal and credible findings.
