# Supplementary material for: The experience of shared decision‐making for people with asthma: A systematic review and metasynthesis of qualitative studies
Source: Health Expect. 2024 Apr 13;27(2):e14039. doi: 10.1111/hex.14039 (PMC11015866; doi:10.1111/hex.14039)
Supplement: Supplementary file 7 — Supporting information. [file HEX-27-e14039-s001.docx]

**Supplemental Files**

**Appendix I:** Detailed database description and search strategies.

**Appendix II:** Reasons for exclusion of 17 publications in the full-text screening process. The 17 publications excluded at the full-text screening stage were mainly due to not meeting the inclusion criteria, such as no perspectives on shared decision making, not adult asthma patient. Other reasons for exclusion included not qualitive studies or qualitative themes that could not be extracted.

**Appendix** **III**: Basic characteristics of included studies. The final documents included 19 studies (13 qualitative studies and 6 mixed methods studies). The studies were conducted in different countries, including the United States, Canada, Australia, and Europe. The studies focused on various aspects of the topic of interest, such as the frustrations encountered by y front-line fighters in the fight against the epidemic, their experiences, and recommendations for the future construction and management of elderly institutions based on their experiences.

**Appendix IV:** Results of included documents and interviewer perceptions. The final 19 included studies consist of 250 findings related to the topics of interest.

**Appendix** **V**: Integration of study results, appendix 5 consolidates the 250 findings in appendix 4 into three main categories and assesses the evaluable of credibility to each one

**Appendix VI:** Appendix VI provides a summary of the ConQual (confidence in qualitative evidence) assessment, where the ConQual score reflects confidence in the qualitative evidence for each outcome based on factors such as study design, data adequacy, and consistency of study results.

**Table 1:** Table 1 provides a quality assessment of the studies included in this review.

**Table 2：**Table 2 provides the extracted information about the 19 extracted studies, the extracted information includes author and year, methodology, methods, platform and length of interview, Country, population, patient gender, aim of the selected studies, place of data collection, and result theme.
